# Study protocol for a multicentre longitudinal mixed methods study to explore the Outcomes of ChildrEn and fAmilies in the first year after paediatric Intensive Care: the OCEANIC study

**DOI:** 10.1136/bmjopen-2020-038974

**Published:** 2020-05-17

**Authors:** Joseph C Manning, Jos M. Latour, Martha A.Q. Curley, Elizabeth S. Draper, Tahseen Jilani, Philip R Quinlan, R. Scott Watson, Janet E. Rennick, Gillian Colville, Neethi Pinto, Asam Latif, Emma Popejoy, Jane Coad, for the OCEANIC Study Investigators

**Affiliations:** 1 Children and Young People Health Research, School of Health Sciences, University of Nottingham, Nottingham, Nottinghamshire, UK; 2 Nottingham Children’s Hospital, Nottingham University Hospitals NHS Trust, Nottingham, Nottinghamshire, UK; 3 Health Data Research UK, University of Nottingham, Nottingham, Nottinghamshire, UK; 4 School of Nursing and Midwifery, University of Plymouth, Plymouth, UK; 5 Nursing Department, Hunan Children’s Hospital, Changsha, Hunan, China; 6 Department of Family and Community Health, School of Nursing, University of Pennsylvania, Philadelphia, Pennsylvania, USA; 7 Anesthesia and Critical Care Medicine, Perelman School of Medicine, University of Pennsylvania, Philadelphia, Pennsylvania, USA; 8 The Research Institute, Children's Hospital of Philadelphia, Philadelphia, PA, USA; 9 Department of Health Sciences, University of Leicester, Leicester, Leicestershire, UK; 10 Advanced Data Analysis Centre, University of Nottingham, Nottingham, Nottinghamshire, UK; 11 Division of Pediatric Critical Care Medicine, Department of Pediatrics, University of Washington, Seattle, Washington, USA; 12 Centre for Child Health, Behaviour, and Development, Seattle Children's Research Institute, Seattle, Washington, USA; 13 Ingram School of Nursing, McGill University Faculty of Medicine, Montreal, Quebec, Canada; 14 Centre for Outcomes Research & Evaluation, Research Institute of the McGill University Health Centre, Montreal, Quebec, Canada; 15 Paediatric Psychology Service, St Georges University Hospitals NHS Foundation Trust, London, UK; 16 Population Health Research Institute, University of London St George’s, London, UK; 17 Section of Pediatric Critical Care, Department of Pediatrics, University of Chicago, Chicago, Illinois, USA; 18 School of Health Sciences, University of Nottingham, Nottingham, Nottinghamshire, UK

**Keywords:** paediatric intensive & critical care, statistics & research methods, qualitative research

## Abstract

**Introduction:**

Annually in the UK, 20 000 children become very ill or injured and need specialist care within a paediatric intensive care unit (PICU). Most children survive. However, some children and their families may experience problems after they have left the PICU including physical, functional and/or emotional problems. It is unknown which children and families experience such problems, when these occur or what causes them. The aim of this mixed-method longitudinal cohort study is to understand the physical, functional, emotional and social impact of children surviving PICU (aged: 1 month–17 years), their parents and siblings, during the first year after a PICU admission.

**Methods and analysis:**

A quantitative study involving 300 child survivors of PICU; 300 parents; and 150–300 siblings will collect data (using self-completion questionnaires) at baseline, PICU discharge, 1, 3, 6 and 12 months post-PICU discharge. Questionnaires will comprise validated and reliable instruments. Demographic data, PICU admission and treatment data, health-related quality of life, functional status, strengths and difficulties behaviour and post-traumatic stress symptoms will be collected from the child. Parent and sibling data will be collected on the impact of paediatric health conditions on the family’s functioning capabilities, levels of anxiety and social impact of the child’s PICU admission. Data will be analysed using descriptive and inferential statistics. Concurrently, an embedded qualitative study involving semistructured interviews with 24 enrolled families at 3 months and 9 months post-PICU discharge will be undertaken. Framework analysis will be used to analyse the qualitative data.

**Ethics and dissemination:**

The study has received ethical approval from the National Health Services Research Ethics Committee (Ref: 19/WM/0290) and full governance clearance. This will be the first UK study to comprehensively investigate physical, functional, emotional and social consequences of PICU survival in the first-year postdischarge.

Clinical Trials Registration Number: ISRCTN28072812 [Pre-results]

**Strengths and limitations of this study**

- The Outcomes of ChildrEn and fAmilies in the first year after paediatric Intensive Care (OCEANIC) study will be the first multisite, comprehensive study conducted in the UK to investigate the physical, functional, emotional and social consequences of paediatric intensive care unit (PICU) survival in the first-year postdischarge.
- Our longitudinal study design will allow us to look at changes over time in the same patient/family, providing insights into the temporal sequence of changes that may occur as a result of childhood critical illness/injury.
- The qualitative study (interviews with children, parents and siblings) will be analysed in conjunction with quantitative data allowing a fuller understanding of physical, functional, emotional and social consequences of being on PICU and any outstanding needs.
- The primary limitation of this study is loss to follow-up and missing data points that would challenge the internal validity of reported results from the OCEANIC study.

## Introduction

In the UK annually, approximately 20 000 children (aged 0–18 years) experience a critical illness, requiring paediatric intensive care unit (PICU) treatment and care.^1^ Despite increasing demand on paediatric critical care services, PICU survival has increased substantially over the past three decades, rendering mortality alone an insufficient metric for outcomes assessment post-PICU discharge.^2^ Over 96% of children admitted to PICU survive.^1^ However, the decline in mortality has been accompanied by a concomitant increase in morbidity.^3^ Evidence is building which portrays a cohort of PICU survivors who are physically deconditioned, cognitively impaired and emotionally distraught. The emotional and social health of the PICU survivor’s parents and siblings may also be affected.^4 5^

Two systematic reviews reported that approximately 25% of critically ill children exhibited negative psychological and behavioural responses within the first-year postdischarge.^6 7^ Similar themes were identified in a systematic review of qualitative studies examining the psychosocial impact of PICU hospitalisation on children,^8^ lending support to the importance in identifying children suffering from psychological sequelae. Given that psychological well-being is shaped by multiple factors, alterations in the child’s sense of self and interpersonal relationships, as well as ongoing worries and fears about hospitalisation, have the potential to affect recovery during the early postdischarge period, and during critical periods of growth and development. Health-related quality of life studies identify deterioration in the emotional well-being of 20%–30% of children up to 1-year post-PICU discharge,^6 7^ suggesting a sustained effect.

The impact of a child’s critical illness on family members may be profound as they, too, can experience psychosocial sequelae.^5 9^ Family members’ responses may, in turn, influence the outcomes of child survivors following paediatric critical illness. Furthermore, there is evidence that critical illness impacts a family’s social functioning in relation to reintegration with peers, the child and family’s social capital and the economic impact of unemployment on families when a caregiver has to relinquish work responsibilities to care for a child.^10^ However, the interplay among the child, their parent and siblings’ outcomes, caregiver roles and family needs, and how these change over time are largely absent in the literature.

Globally^11–13^ and in the UK,^14 15^ researchers, clinicians and patients and their families have recognised understanding and supporting adult survivors of intensive care is both a research and clinical priority. Both patient and public consultation, and a national survey, conducted with the PICU community (including children, their families, service providers and commissioners) confirms that understanding and optimising the outcomes of children and their families are also a research priority for childhood survivors of PICU.^16 17^

## Methods and analysis

### Study purpose and objectives

The purpose of the Outcomes of ChildrEn and fAmilies in the first year after paediatric Intensive Care (OCEANIC) study is to explore child PICU survivors’ health outcomes and family impact over 1-year post-PICU discharge.

OCEANIC has four specific objectives:

1. To describe the physical, cognitive, emotional and social health outcomes and trajectory of recovery in children post-PICU discharge.
2. To determine the baseline and PICU factors associated with impaired outcomes.
3. To explore the longitudinal emotional and social health outcomes of parents and siblings.
4. To ascertain the care and support needs of children and their parents and siblings.

#### Theoretical framework

**Figure 1:**
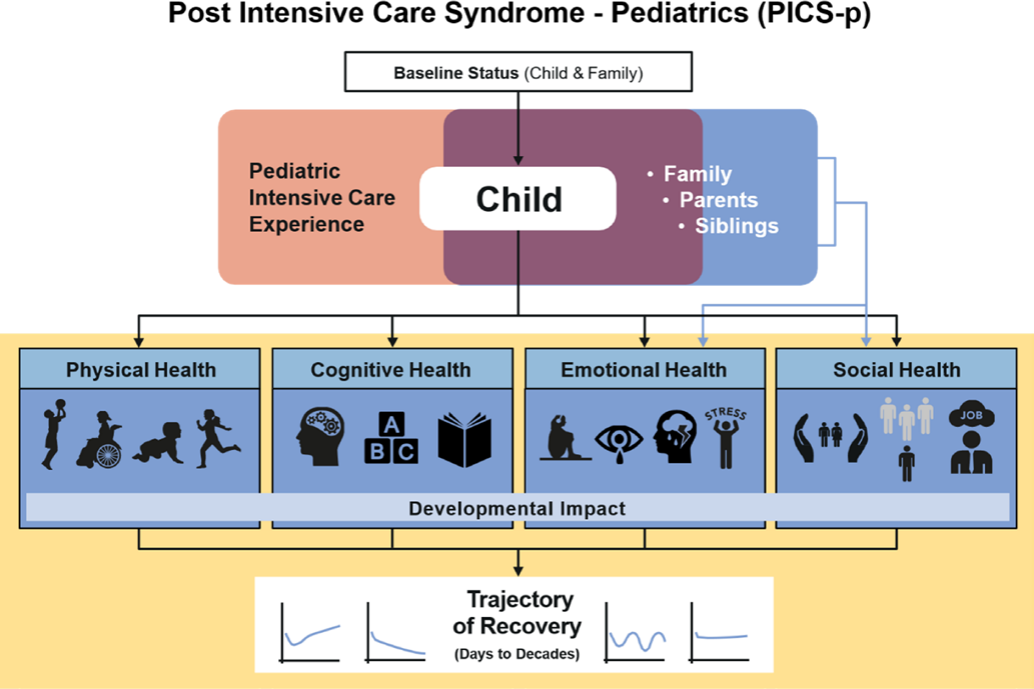
Postintensive care syndrome in paediatrics (PICS-p) framework.^19^

Based on a state-of-the-science review of postdischarge outcomes in paediatric critical care,^18^ a conceptual framework describing the constellation of potential physical, cognitive, emotional and social health effects that may be uniquely experienced by children and families who survive paediatric critical illness has been proposed (figure 1).^19^ This framework incorporates the importance of pre-existing health status, sociodemographic data, physiological maturation and psychosocial development on the trajectory of health recovery over a child’s lifetime. Additionally, the framework recognises that the interdependence of the child and family is central to understanding the long-term multidimensional sequelae of paediatric critical illness. This framework provides a roadmap for understanding longitudinal outcomes; the proposed study will organise data collection using this framework.

**Figure 2:**
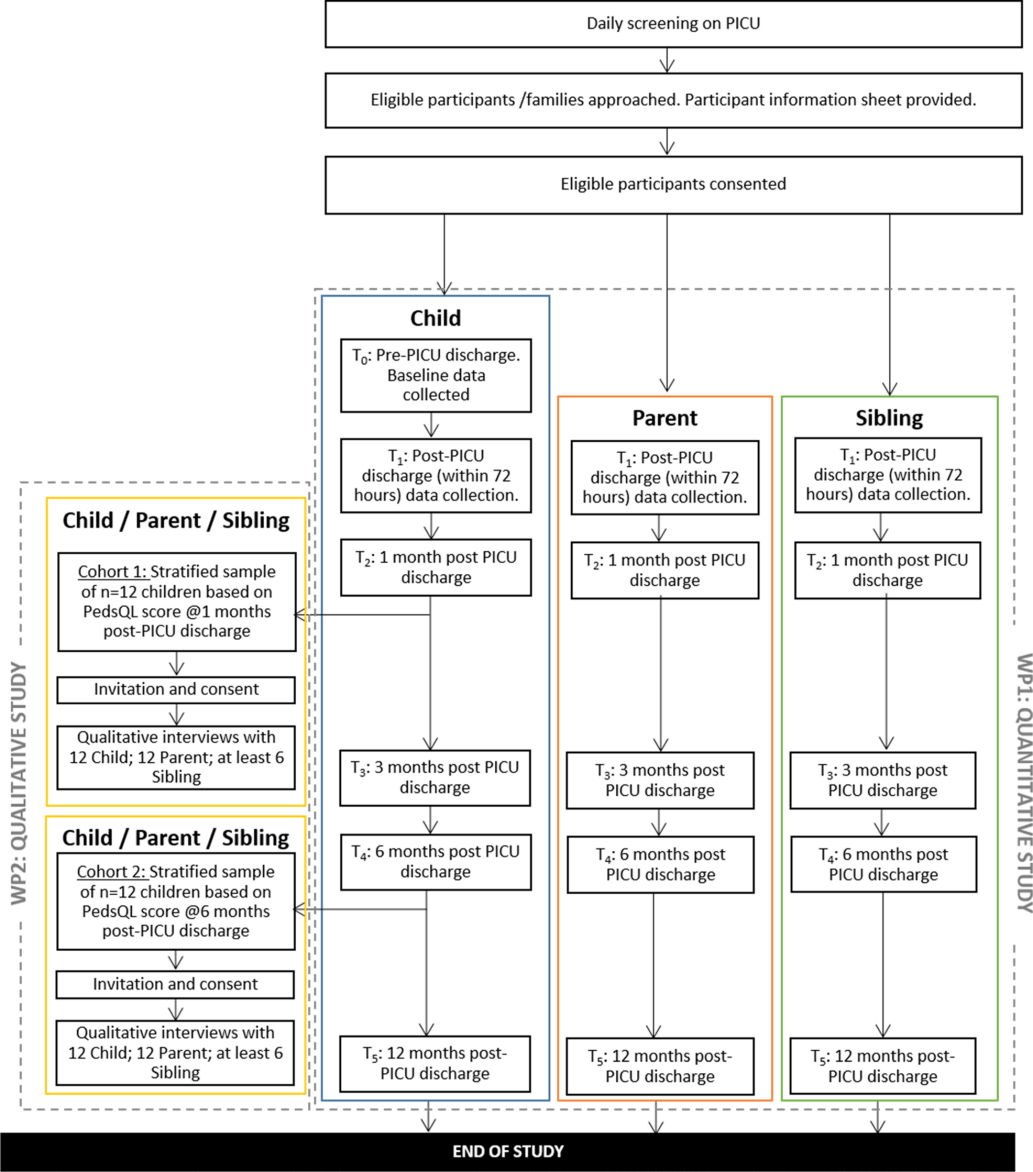
Overview of linked work packages of the Outcomes of ChildrEn and fAmilies in the first year after paediatric Intensive Care study. PedsQL, Pediatric Quality of Life Inventory; PICU, paediatric intensive care unit.

This embedded mixed-method study involves two linked work packages (overview presented in figure 2). The first work package will be a quantitative study involving 300 child survivors of critical illness, 300 parents and 150–300 siblings. The second work package will be a qualitative interview study of 2 cohorts of 12 families, at 3 and 9 months post-PICU discharge. Mixing will occur through the sampling and selection of participants for the embedded qualitative study from those enrolled in the quantitative study, as well as in the framework analysis.

### Quantitative study

Data regarding the PICU admission of each child participant will be downloaded from the Paediatric Intensive Care Audit Network (PICANet) database, a secure and confidential high-quality clinical database of paediatric intensive care activity in the UK and Ireland. Data extracted will include: demographic and socioeconomic data, pre-PICU health status and acute illness data (PICU admission and discharge diagnoses; comorbidities; operations and invasive procedures performed; type of admission (planned/unplanned); PICU and hospital length of stay (LOS), duration of mechanical ventilation, high-frequency oscillatory ventilation, extracorporeal membrane oxygenation, renal replacement therapy and vasopressor/inotropic support; sedative medications and days of exposure). Outcome data will also be collected from each child (or proxy), their parent and sibling (if appropriate) prospectively over the first-year post-PICU discharge.

### Study measures

Currently, there are no standardised or agreed set of outcome measures for research with the PICU patient population. Therefore, the outcome measures used in this study were selected for their validity, reliability, ease of use, availability in electronic versions and previous use with the population under investigation. Furthermore, the focus and selection of these measures were informed by the postintensive care syndrome in paediatrics framework, contemporary literature and consultation with patients, public and PICU clinicians. In line with feedback from patient and public involvement (PPI) consultations, outcomes will be collected at six time points: baseline status (pre-PICU discharge); at PICU discharge; 1, 3, 6 and 12 months post-PICU discharge. The outcomes measured and time points are outlined in table 1.

**Table 1:** Data collection measures and time points in which data are collected for child paediatric intensive care unit (PICU) survivor, parent/legal guardian and sibling

	Version	Items/time required	T_0_: baseline (retrospective)	T_1_: PICU discharge	Post-PICU discharge			
					T_2_: 1 month	T_3_: 3 months	T_4_: 6 months	T_5_: 12 months
Section 1: child–survivor measures								
1. Pediatric Quality of Life Inventory (PedsQL) Infant Scales Version 4.0—Acute (aged: 1–23 months)	Infant 1–12 months	36 items/<7 min	X	X	X	X	X	X
	Infant 13–23 months	45 items/<10 min						
OR								
2. PedsQL Generic Core Scales Version 4.0—Acute (aged: 2 years+)	Toddlers	21 items/<5 min						
	Young Child	23 items/<5 min						
	Child	23 items/<5 min						
	Teen	23 items/<5 min						
3. PedsQL Multidimensional Fatigue Scale Version 3.0—Acute		18 items/5 min	X	X	X	X	X	X
4. PedsQL Pediatric Pain Questionnaire		1 item/<1 min		X	X	X	X	X
5. Functional Status Scale		6 items/5 min	X	X	X	X	X	X
6. Paediatric Overall Performance Category and Paediatric Cerebral Performance Category		2 items/5 min	X	X	X	X	X	X
7. Strengths and Difficulties Questionnaire (SDQ)		25 items/4 min		X	X	X	X	X
8. Child Impact of Events Scale		8 items/4 min				X	X	X
9. Children’s Hope Scale (CHS)		6 items/3 min		X	X	X	X	X
Max. total number of measures:			4	7	7	8	8	8
*Note bene (NB) for qualitative study a sample of child survivors will take part in one semistructured interview lasting approximately 30–60 min at either 1–3 months or 6–9 months post discharge*								
Section 2: parent/legal guardian measures								
1. PedsQL Family Impact Module Version 2.0		36 items/5 min		X	X	X	X	X
2. State-Trait Anxiety Inventory (Y-6 item)		6 items/2 min		X	X	X	X	X
3. Patient Health Questionnaire-4		4 items/2 min		X	X	X	X	X
4. PTSD Checklist−5		17 items/5 min				X	X	X
Total number of measures:			–	3	3	4	4	4
*NB for* *qualitative study a sample of parents will take part in one semistructured interview lasting approximately 30–60 min at either 3 months or 9 months post discharge*								
Section 3: sibling measures								
1. PedsQL Version 4.0 Generic Core Scales		23 items/4 min		X	X	X	X	X
2. SDQ		25 items/4 min		X	X	X	X	X
3. Multidimensional Assessment of Caring Activities (YC18)		18 items/2–4 min		X	X	X	X	X
4. Positive and Negative Outcomes of Caring (YC20)		20 items/2–4 min		X	X	X	X	X
5. CHS		6 items/3 min		X	X	X	X	X
Total number of measures:			–	5	5	5	5	5
*NB for qualitative study a sample of siblings will take part in one semistructured interview lasting approximately 30–60 min at either 3 months or 9 months post discharge*								

PTSD, post-traumatic stress disorder.

Data collection measures, versions and report format according to age and study participant (child PICU survivor, parent/legal guardian or sibling) are reported in table 2. A brief overview of the measures is provided in online supplementary file 1.

10.1136/bmjopen-2020-038974.supp1 Supplementary data

**Table 2:** Data collection measures, versions and report format according to age and study participant (child paediatric intensive care unit (PICU) survivor, parent/legal guardian or sibling)

Measure/version (reported by)		1–12 months	13–23 months	2–4 years	5–7 years	8–10 years	11–12 years	13–17 years
Section 1: child PICU survivor								
		PICU survivor participant age						
1. Pediatric Quality of Life Inventory (PedsQL) Infant Scales Version 4.0—Acute								
Infants 1–12 months	(Parent reported)	X						
Infants 13–24 months	(Parent reported)		X					
2. PedsQL Generic Core Scales Version 4.0—Acute								
Toddlers	(Parent reported)			X				
Young child	(Child or parent reported)				X			
Child	(Child or parent reported)					X	X	
Teen	(Child or parent reported)							X
3. PedsQL Multidimensional Fatigue Scale Version 3.0—Acute								
Toddlers	(Parent reported)			X				
Young child	(Child or parent reported)				X			
Child	(Child or parent reported)					X	X	
Teen	(Child or parent reported)							X
4. PedsQL Pediatric Pain Questionnaire								
Young child	(Child or parent reported)				X			
Child	(Child or parent Reported)					X	X	
Teen	(Child or parent reported)							X
5. Functional Status Scale	(Parent reported)	X	X	X	X	X	X	X
6. Paediatric Cerebral Performance Category and Paediatric Overall Performance Category	(Parent reported)	X	X	X	X	X	X	X
7. Strengths and Difficulties Questionnaire (SDQ)								
2–4 years old	(Parent reported)			X				
4–17 years old	(Parent reported)				X	X		
11–17 years old	(Child reported)						X	X
8. Child Impact of Events Scale	(Child reported)					X	X	X
9. Children’s Hope Scale (CHS)	(Child reported)					X	X	X
Section 2: parent/legal guardian								
						Parent/legal guardian		
1. PedsQL Family Impact Module Version 2.0—Acute				(Parent reported)		X		
2. State-Trait Anxiety Inventory (Y-6 item)				(Parent reported)		X		
3. Patient Health Questionnaire-4				(Parent reported)		X		
4. PTSD Checklist (PCL)−5				(Parent reported)		X		
Section 3: sibling								
Sibling participant age		
1. PedsQL Generic Core Scales Version 4.0—Acute								
Child				(Child reported)		X	X	
Teen				(Child reported)				X
2. SDQ								
4–17 years old				(Parent reported)		X		
11–17 years old				(Child reported)		X	X
3. Multidimensional Assessment of Caring Activities (YC18)				(Child reported)		X	X	X
4. Positive and Negative Outcomes of Caring (YC20)				(Child reported)		X	X	X
5. CHS				(Child reported)		X	X	X

PTSD, post-traumatic stress disorder.

Child related measures include:

- Pediatric Quality of Life Inventory (PedsQL 4.0) Generic Core Scales (2–17 years) and Infant Scales (1–23 months)—Acute Version.^3 20–29^
- PedsQL Multidimensional Fatigue Scale (2–17 years)—Acute Version.^30^
- PedsQL Pediatric Pain Questionnaire (5–17 years).
- Functional Status Scale (1 month–17 years).^31–33^
- Paediatric Cerebral Performance Category and the Paediatric Overall Performance Category (1 month–17 years).^34–37^
- Strengths and Difficulties Questionnaire (SDQ; 2–17 years).^38 39^
- Child Revised Impact of Events Scale (7–17 years).^40–42^
- Children’s Hope Scale (CHS; 8–17 years).^43^

Parent-related measures:

- PedsQL Family Impact Module Version 2.0.^44^
- State-Trait Anxiety Inventory 6.^45^
- Patient Health Questionnaire-4.^46^
- The Post-Traumatic Stress Disorder Checklist (PCL-5) for Diagnostic and Statistical Manual of Mental Disorders-5 (DSM-V).^47–49^

Sibling-related measures:

- PedsQL 4.0 Generic Core Scales (2–17 years).^3 20–29^
- CHS (8–17 years).^43^
- Multidimensional Assessment of Caring Activities (YC18; 8–17 years).^50 51^
- Positive and Negative Outcomes of Caring (YC20; 8–17 years).^51^

### Qualitative study

The second work package will be a qualitative study involving semistructured interviews with 24 families, split between 3 and 9 months post-PICU discharge. As advocated in the child health literature, a pragmatic and participant-centred approach (based on choice, participation and flexibility) to collecting qualitative data will be employed. Interviews will be conducted with children, parents/legal guardians and siblings either collectively or separately. Interviews will take place at the participants’ preferred time and method (eg, face to face, telephone). The use of multiple sources of data will provide contextualised, converging and emerging lines of inquiry.

### Sample and recruitment

#### Setting

Participants will be recruited from at least five PICUs across England chosen to include variation in unit size, case mix, geographical location and patient demographic.

#### Eligibility criteria

Participants for this study include: (1) PICU child survivors, (2) parents/legal guardians and (3) siblings.

1. PICU child survivor:(a) Aged 1 month (and ≥44 weeks corrected gestational age) to 17 years at the point of PICU admission; (b) will be discharged from the PICU in next 48 hours; (c) PICU total LOS≥72 hours at point of discharge in which the patient received PICU therapies for organ dysfunction; (d) at least one parent/legal guardian (≥18 years of age or considered emancipated) living with the potential subject.
2. Parent:(a) Parent or legal guardian; (b) cohabits with the child.
3. Siblings:(a) Aged ≥8 years (at baseline); (b) is a sibling of the children PICU survivor; (c) cohabits with the child PICU survivor for at least 50% of the time; (d) can independently self-report.

### Sample

#### Sample size

Quantitative study: we anticipate enrolling 300 children (and their families) from 5 PICUs in equal proportions (60 per centre) over a 6-month period. Based on previous PICU studies,^52 53^ we conservatively estimate a 20% attrition rate over 1 year. Thus, we anticipate having 1-year outcomes for 240 patients at the end of the study. With 240 participants, we will have high power to detect small/moderate correlations between early PedsQL measurements (to assess the trajectory of recovery) and other baseline and PICU factors with 1-year PedsQL summary scores. Using a two-sided 0.05 level test, we have 80% power to detect correlations of 0.18 or larger in magnitude. With 240 participants, we will also have high power to detect moderate differences when comparing two groups using a t-test (eg, comparison of PedsQL summary scores by gender or diagnosis category). In addition, many of the analyses will involve multiple linear regression modelling to adjust for baseline factors or confounding variables. With 240 participants, there is high power for the assessment of modest covariate effects with linear regression. Thus, we anticipate having high power for assessing correlations or linear regression effects as well as for comparing groups with our expected 1-year sample size.

Qualitative study: a stratified sample of up to 24 families (which may include the child, parent and sibling, with a maximum of 72 participants in total) will be enrolled into the qualitative interviews. This sample size will capture diverse perspectives around support needs and is expected to achieve data saturation in the qualitative analysis.^54^

#### Sampling technique

Quantitative study sampling technique: a consecutive sampling strategy will be employed.^55^ Each site will screen daily over a 12-month period and invite all eligible children to participate in the study. Data from screening logs, including refusal to participate and admission numbers at each site, will be collected and used to contextualise the reporting of the analysis. In order to recruit a sample that is representative of the PICU populous, a sampling frame based on age and diagnosis reported from PICANet data^1^ will be used. This frame will be used to guide the recruitment of participants recruited into the study and is outlined in table 3.

**Table 3:** Proposed sampling frame for paediatric intensive care unit survivor participant recruitment

	Diagnosis				
Age (years)	Cardiovascular (28.1%)	Neurological (10.7%)	Respiratory (29.2%)	Other* (32%)	Total
0 (55%)	47	19	48	53	167
1–5 (25.2%)	21	8	23	25	77
6–10 (9.7%)	8	3	8	9	28
≥11 (10.3%)	8	3	8	10	28
Total	84	33	87	63	300

*Including: blood/lymphatic; body wall and cavities; endocrine/metabolic; trauma; oncology; musculoskeletal; multisystem; infection; gastrointestinal.

Qualitative study sampling technique: two cohorts of 12 families (including the child, parent and a sibling) will be selected using a stratified sampling approach based on the child’s PedsQL score at 1-month post-PICU discharge and 6-month post-PICU discharge. Stratification using previously reported norms for PedsQL as well as variation in relation to geographical locality, PICU presenting condition, age and ethnicity will be sought.

### Study procedures

#### Quantitative study

Over a 6-month period, each site will screen daily the children admitted to PICU and invite all eligible children to participate in the study. Site investigators (or their designated nominee) who are part of the PICU clinical care team will determine eligibility.

In line with feedback from PPI work in the development of this study, each participant (aged ≥5 years) will be provided with a single £15 gift voucher as a token of appreciation for participating in the study. Vouchers will be provided to all participants on the completion of the study data collection period (T6—12 month’s post-PICU discharge).

#### Qualitative study

**Figure 3:**
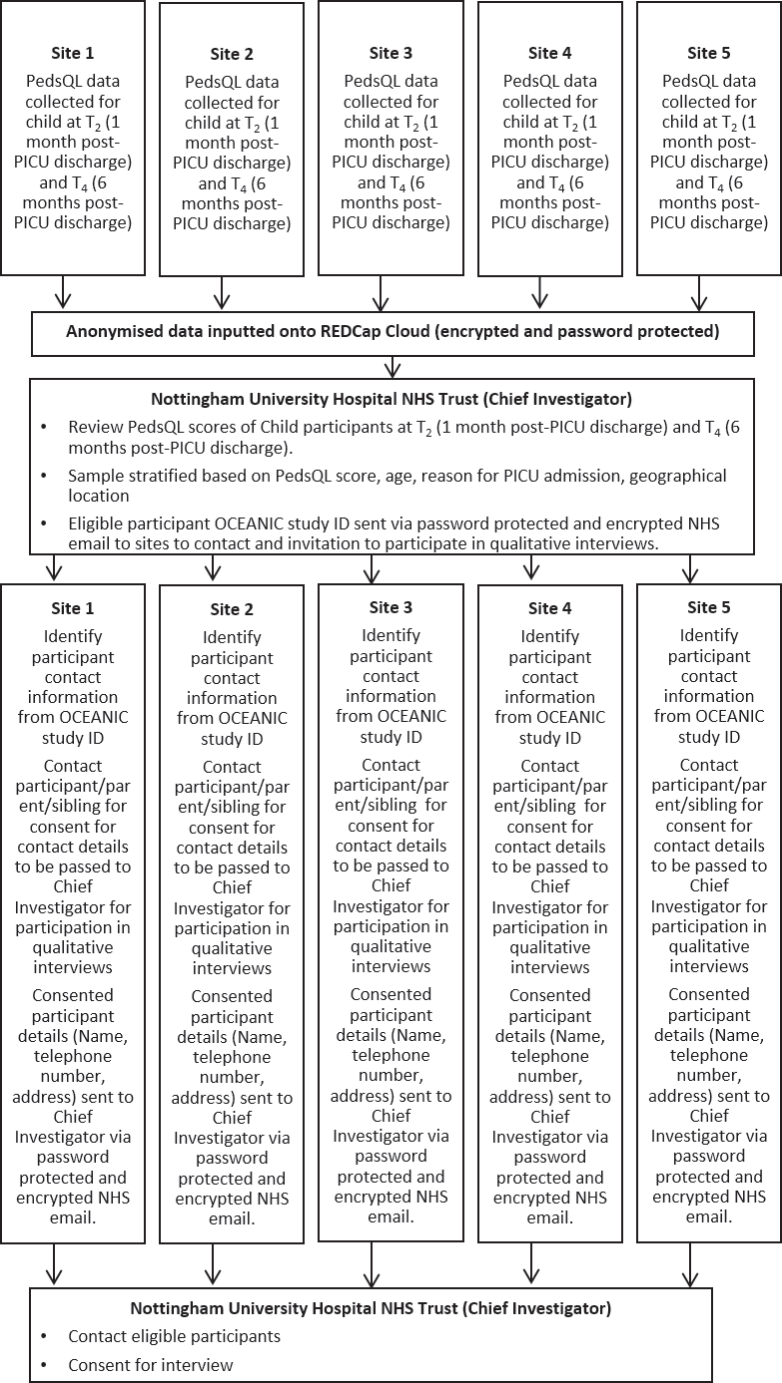
Identification (ID) and recruitment of participants for Qualitative Study. NHS, National Health Service; OCEANIC, Outcomes of ChildrEn and fAmilies in the first year after paediatric Intensive Care; PedsQL, Pediatric Quality of Life Inventory; PICU, paediatric intensive care unit.

For the qualitative study, participants will be identified from PedsQL scores of the child participant at 1-month post-PICU discharge and 6-month post-PICU discharge. The identification and recruitment process are summarised in figure 3 and will follow a systematic process:

1. Child participant PedsQL scores will be collected and submitted by sites onto REDCap Cloud.
2. The chief investigator will review the scores and stratify the sample based whether the PedsQL score is within 1, 2 or >2 SD from the published norms, selecting at least 4 children for each group at 1-month post-PICU discharge and 6-month post-PICU discharge. To maximise diversity in families (child, parent and sibling) interviewed, where possible participants will be selected based on geographical locality, PICU presenting condition, age and ethnicity.
3. The study ID of potential participants will be sent to sites, who will then contact the family directly, requesting consent to receive contact from the chief investigator/study researcher.
4. The chief investigator/study researcher will contact families that have agreed to being contacted, to consent for qualitative interviews and to arrange suitable date, time and location.

### Analyses

#### Quantitative study data analysis

Descriptive statistics will be presented for demographic information, and medical history. All child, parent and sibling-related measures will be calculated, including means, SD, medians and IQRs for continuous variables and frequency counts and percentages for categorical variables. Data will be examined for normality, outliers and systematic missing data. Transformations will be undertaken as needed.

Analyses related to specific objectives include the following:

*Objective 1: To describe the physical, cognitive, emotional and social health outcomes and trajectory of recovery in children post-PICU discharge*. The primary aim is to explore child PICU survivors’ health outcomes and trajectory of recovery over the first-year post-PICU discharge. PICU survivors’ health outcomes will be compared with published population means from the general and chronically ill populations using t-tests or Mann-Whitney test as appropriate. For the longitudinal data, correlations will be assessed between time points using Spearman correlations and a linear mixed regression model with random subject effects will be used to analyse trajectories over time. In case of lack of normality, the non-parametric longitudinal approach will be implemented.

*Objective 2: To determine the baseline and PICU factors associated with impaired outcomes*. To identify factors associated with impaired health outcomes among PICU survivors, correlation analyses followed by principle component analysis (PCA) will be applied to identify covariates for the regression modelling. For categorised recovery over 1-year post-PICU discharge, mixed effect logistic regression will be applied. Variables will be entered using backward stepwise approach to control for collinearity. Model performance will be assessed using sensitivity, specificity, positive predicted value, negative predicted value and Area Under Curve Reciever Operating Characteristics (AUCROC) values. Bootstrapping through K-fold approach will be applied to ensure better modelling.

*Objective 3: To explore the longitudinal emotional and social health outcomes of parents and siblings*. Parent and sibling emotional- and social health outcomes will be compared with published means using t-tests or Mann-Whitney test as appropriate. PICU survivor and sibling PedsQL summary scores and SDQ scores will also be compared using paired t-tests or Wilcoxon Signed Rank test.

Graphical analyses will be performed to display the trajectories of health outcomes over time in our populations of critically ill children. Multiple linear and logistic regression methods will be used to explore the effects of primary diagnosis (eg, respiratory, cardiovascular), PICU LOS category, and site, to predict outcomes. We will explore whether adjustment for sex, race/ethnicity or site affects study inferences through the use of mixed effects and generalised estimating equations models. Finally, we will also explore the use of classification and regression trees with recursive partitioning, PCA, factor analysis and machine learning methods to help describe subgroups of patients with similar trajectories of outcome.

#### Qualitative study data analysis

Audio recorded interview data will be transcribed verbatim with all participant identifiable information removed. Transcription will be conducted by a service approved by Nottingham University Hospitals National Health Service (NHS) Trust Research and Innovation Department. Confidentiality agreements will be completed. Transcripts will be imported into NVivo V.12, for sorting, coding and categorising of the data.

Qualitative data will be analysed using the adapted five-stage framework analysis process to achieve *objective 4*; identification of the care and support needs of children, their parents and siblings. The five stages of framework analysis comprise (1) familiarisation with the data through reading full transcripts; (2) development of a theoretical framework through identification of recurring and important themes; (3) indexing and pilot charting; (4) summarising data in an analytical framework; and (5) synthesising data by mapping and interpreting.^56^ Stages 1–4 will be conducted separately for respondent type (children, parents or siblings) to enable specific care and support needs to be identified and summarised. Stage 5 will then allow for data to be compared and contrasted across the respondent groups (child, parent, sibling), child’s PedsQL score (<1, 2, or >2 SD from published norms), and time points (1–3 months or 6–9 months post-PICU discharge).

### Patient and Public Involvement

Underpinned by the best principles of National Institute for Health Research (NIHR) INVOLVE (https://www.invo.org.uk/), children, young people (CYP) and families have been integral to the development of this study. In 2017, the chief investigator (Dr Manning) and co-investigator (Professor Latour) organised the UK’s first symposium on aftercare and rehabilitation following PICU and engaged with over 60 PICU clinicians, an ex-PICU patient and family members. Feedback identified that: a prospective longitudinal cohort study to further understand the outcomes for CYP and their families post-PICU was needed; and the collection of data at multiple time points over the first year would have value for CYP and their families, health professionals and research to direct the development of future interventions.

Further PPI has been undertaken with 11 parents (seven mothers and four fathers), 4 siblings (aged 9–13 years) and 3 CYP PICU survivors (aged 11–17 years) from the East and West Midlands. Participants’ varied in ethnicity and family composition, and reasons for admissions to different PICUs. The proposed study was regarded as addressing an important topic. Respondents main concerns included: the potential to trigger negative reactions from participation; the collection of information pertaining to the preintensive care unit state and the difficulty of considering their own emotional well-being when their focus is on their child’s survival. Suggestions to address these included: certificates and vouchers to thank participants, flexibility in the method of data collection, linking up with existing support services to build reminders and removing reference to scores within the survey/s. Making the purpose of the research more visible through study website and social media would help parents’ make decisions about participating and keeping updated with the study.

As part of this study, we will continue to have meaningful advice and input from PPI. An advisory group has been assembled consisting of a young person that has been critically ill, parents and carers of children that have experienced critical illness/injury, and a sibling of a critical illness survivor. It is proposed that this group will have at least 6 monthly meetings to ensure they have continued and active involvement in: the management of the research; developing participant information resources; contributing to the study report and dissemination of research findings.

## Ethics and dissemination

### Ethics

This research includes recruitment of seriously ill children on a PICU and a parent and sibling. It concerns a challenging topic requiring great skill and sensitivity in data collection. The study is being carried out by an experienced research team with clinical and research expertise in children and young people who are seriously ill. Research staff will have also received one-to-one protocol training with the CI. We will ensure the first approach is from a member of the child’s usual care team, and is sensitive to the situation and status of the child.

PPI is central to this project and in ensuring that it remains grounded in the experiences of patients. The associated participant facing materials will be carefully developed (with age specific information sheets and consent/assent forms) and these will be reviewed by a PPI panel. The information sheets clearly state that discussing the experience of serious illness may be distressing, and we will ask participants to consider carefully how they feel about this prospect before deciding to take part.

#### Consent/assent

Eligible participants will be given at least 24 hours to consider whether they wish to participate in the study. It will be made clear to the parents that they will be free to withdraw their consent for their own and/or their child’s participation in the study at any time without this having any impact on their child’s care. The majority of children will be sedated and on a ventilator at recruitment, therefore will be unable to provide informed consent/assent.

For those children unable to provide consent/assent at the time of enrolment into the study, consent will be obtained from their parent/legal guardian. Efforts will be made to then consent/assent the child once they are able to (eg, have the cognitive capacity) by the site teams. In the unlikely event that a child does not wish to participate (and the parent has consented for the child), the child’s wishes will be upheld and the parent/sibling will be withdrawn from the study.

#### Interviews

We recognise that the discussing/recollecting a potentially difficult experience (the PICU admission) and any ongoing health and care needs may be upsetting for survivors/parents/families.^10^ Therefore, all interviews will be conducted by the chief investigator or the OCEANIC Research Fellow, who both have previous experience of conducting interviews with children and families on sensitive issues. Interviews will be semistructured over 30–60 min with appropriate breaks if necessary. Interviews will allow participants to explore any issues in depth, which in itself may provide opportunity for issues, feelings and emotions to be discussed. This will be facilitated by creative/child centred data collection techniques that are sensitive to exploring potentially emotive events, in a constructive manner. Families will be given the choice whether they would like to have the interview separately (child, parent and sibling) or collectively.

It will be made clear to participants at the outset that the interview can be stopped at any time should they wish. Furthermore, if the child participant, their parent/legal guardian, or sibling becomes visibly upset during the interview, the investigator will:

1. Invite the parent/legal guardian (if present) to console the child/sibling, (if not already doing so).
2. Offer to temporarily stop or terminate the visit.
3. Respect the decision made by the participant to stop/carry on the interview.

All visits with children (<16 year olds) will be conducted with the parent/legal guardian present. In cases where it is not possible for parents to be present or the child specifically requests for them not to be present, a second investigator from the study team will be present. All the study investigators have an enhanced Disclosure and Barring Service check. All investigators conducting the qualitative interviews are registered with Nursing and Midwifery Council (UK, first level) and are therefore bound by codes of professional conduct and have a professional obligation to share information with other agencies (ie, social services), if an interview participant discloses information that relates to safeguarding or child protection.

#### Ethical review

The West Midlands—The Black Country NHS Research Ethics Committee has reviewed the study protocol and provided favourable opinion (Ref: 19/WM/0290). The Health Research Authority has also approved the protocol (IRAS: 269642). This study has been externally peer reviewed and awarded funding through a competitive process through the National Institute for Health Research (NIHR) (ICA-CL-2018-04-ST2-009). The study has been registered in International Standard Randomised Controlled Trials Number 28072812.

### Dissemination

Despite advances to the evidence base, a comprehensive understanding of PICU morbidity among survivors after PICU discharge remains limited. Historically, studies have focused on specific populations and/or diseases (such as prematurity, congenital heart disease, long-stay patients) rather than on issues experienced by the post-PICU discharge population as a whole.^32 57–63^ Moreover, these studies to date have examined variable outcomes (such as functional status, health-related qualify of life, psychological well-being, adaptive behaviours) at a single time point,^32 57–63^ with few studies considering the patient’s pre-PICU status. Collectively, this heterogeneity in scope severely limits understanding of morbidities experienced by children who survive critical illness, and their trajectories.^27^

While there is a definite need to understand the long-term outcome trajectories of children and families, the scope and purpose of this research are to address this critical gap by being the first study to provide a comprehensive and contemporary understanding of the outcomes of children and families in the first-year post-PICU admission. This will allow for health deficits across a spectrum of domains to be identified. It will provide a better understanding of those at risk of morbidity post-PICU admission, when this manifests, its natural history and any factors that could be modified to improve outcomes. Novel and contemporary insights into the outcomes of children and their family will be established through the study findings, which has been recognised as global priority area for PICU research. Moreover, this study will enhance understanding of the health outcomes of under-researched groups within the PICU populous including those very young children (<2 years), as well as those with communication/developmental impairments. Collectively, characterisation of the longitudinal recovery of children, their parents and siblings post-PICU discharge will allow interventions to be identified to prevent or mitigate morbidity and therefore have the potential to optimise the outcomes and lives of children and their families. Findings will impact on the delivery and configuration of current services, as well as having the potential to inform the development of new models of care that improve the quality of services for patients and families.

The dissemination strategy will be multifaceted to ensure findings are reported in a timely and relevant manner to key stakeholders that include patients and the public, healthcare professionals, commissioners and policy-makers, and academics. Findings will be reported within a funder report (accessible through the NIHR Academy website), professional journals and in high-quality peer-reviewed, open-access journals. In addition, members of the PPI advisory group will assist in composing a summary which will be distributed to national parent support groups and charities. Key findings will also be posted on institutional websites and social media.

## Supplementary Material

Reviewer comments

Author's manuscript
