# Supplementary material for: Study protocol for a multicentre longitudinal mixed methods study to explore the Outcomes of ChildrEn and fAmilies in the first year after paediatric Intensive Care: the OCEANIC study
Source: BMJ Open. 2020 May 17;10(5):e038974. doi: 10.1136/bmjopen-2020-038974 (PMC7239532; doi:10.1136/bmjopen-2020-038974)
Supplement: Supplementary data [file bmjopen-2020-038974supp001.pdf]

The OCEANIC study – Summary of measures used

Child related measures

PedsQL™ 4.0 (Pediatric Quality of Life Inventory) Generic Core Scales *(2-17 years)* and Infant Scales *(1-23 months)* – Acute Version measures HRQOL in children and adolescents aged 1 month to 17 years old. Both sets of instruments have good validity and reliability, have been widely used, [1-8] and can be completed in 5-7 minutes.[8-11] The instruments can discriminate between healthy children and those with a wide range of acute and chronic health conditions.

PedsQL™ Multi-dimensional Fatigue Scale *(2-17 years)* – Acute Version [12] is an 18-item scale that encompasses three domains: (1) General Fatigue, (2) Sleep/Rest Fatigue and (3) Cognitive Fatigue. The Multidimensional Fatigue Scale comprises parallel child self-report and parent proxy-report formats. Items for each of the forms are essentially identical, differing in developmentally appropriate language, or first or third person tense.

PedsQL™ Pediatric Pain Questionnaire *(5-17 years)* is a generic symptom-specific instrument to measure pain in patients with acute and chronic health conditions. We will use question #1 and #2, which asks participants capable of self-reporting to identify a point on a 100 mm line that best shows the worst pain the child experienced 'now' and 'in the past week'. Anchors include "no hurting, no discomfort, or no pain" and "hurting a whole lot, very uncomfortable, severe pain". A parent report version will be used for child participants that are unable to self-report.

Functional Status Scale (FSS) *(1 month-17 years)* is a valid and reliable assessment method to quantify functional status.[13 14] The FSS includes 6 domains: mental status, sensory functioning, communication, motor function, feeding, and respiratory. The FSS is amenable to studies of this nature due to ease of administration, granularity, and objectivity of assessment compared to other available methods and has been used in other outcome studies.[13 15]

Pediatric Cerebral Performance Category (PCPC) and the **Pediatric Overall Performance Category (POPC)** ***(1 month – 17 years)*** quantify short-term cognitive impairments and functional morbidity.[16 17] The POPC scale is dependent on the PCPC scale, as the PCPC status is included in POPC. Scores range from 1 to 6 for both scales with 1: good, 2: mild disability, and 6: brain death. Studies of patients with scores of 1–4 at PICU discharge, hospital discharge,

and one- and six-month follow-up show association with the Stanford Binet Intelligence Quotient, Bayley scales, and Vineland Adaptive Behaviour Scale. [16-19]

Strengths and Difficulties Questionnaire (SDQ) ***(2-17 years)*** [20 21] is a behavioural screening questionnaire used to evaluate emotional symptoms, conduct problems, hyperactivity/inattention, peer relationship problems, and prosocial behaviour. The SDQ quantifies low, medium, and high risk of emotional, behavioural, hyperactivity concentration disorders, or any disorder.

Child Revised Impact of Events Scale (CRIES-8) ***(7-17 years)*** is an eight-item screen for post-traumatic stress symptoms in children aged between 7 and 18 years, with established reliability and validity.[22] It has been used previously in the PICU population.[23] A cut-off score of 17 or greater has been found to classify correctly over 80% of children with a diagnosis of post-traumatic stress disorder.[24]

Children's Hope Scale (CHS) ***(8-17 years)*** [25] is a brief, six-item self-report measure of children's perceptions that their goals can be met. It has been validated for use in children and young people aged 8-17 years consisting of both healthy populations and children with a range of physical illnesses. Internal consistency estimates (alpha) for the samples ranged from 0.72 to 0.86. Test-retest reliability estimates (over a one-month interval) ranged from 0.71 to 0.73 [25].

Parent related measures

PedsQL™ Family Impact Module (FIM) Version 2.0 measures the impact of pediatric health conditions on family functioning [26]. It is completed by the parents and includes eight dimensions (physical functioning; emotional functioning; social functioning; cognitive functioning; communication; worry; daily activities; family relationships).

State-Trait Anxiety Inventory 6 (STAI-6) [27] is a self-reported questionnaire that assesses symptoms of anxiety. It is a short version of the Spielberger State Anxiety Scale (SSA), with a cut-off point at 1 SD above the mean to indicate clinically relevant symptoms[27].

Patient Health Questionnaire-4 (PHQ-4) [28] is a 4 item inventory rated on a 4 point Likert-type scale. Its purpose is to allow for very brief and accurate measurement of depression and anxiety. PHQ–4 scores are strongly associated with decrements in multiple domains of

functional impairment; the anxiety and depression subscales make unique overall contributions to the PHQ–4, both in terms of factorial and criterion validity; and perhaps most importantly: the results indicate that anxiety has a substantial independent effect on functioning, and even more so when comorbid with depression.

The Post Traumatic Stress Disorder (PTSD) Checklist for DSM-5 (PCL-5) is a 20-item self-report measure that assesses the presence and severity of PTSD symptoms [29]. Items on the PCL-5 correspond with DSM-5 criteria for PTSD. The PCL-5 can be used to quantify and monitor symptoms over time, to screen individuals for PTSD, and to assist in making a provisional or temporary diagnosis of PTSD. The PCL-5 is a psychometrically sound measure of DSM-5 PTSD. It is valid and reliable, useful in quantifying PTSD symptom severity, and sensitive to change over time [30 31].

Sibling related measures

The **PedsQL™ 4.0, SDQ** and **Children's Hope Scale** (as outlined in the Child related measures section above) will also be administered to sibling participants.

Multidimensional Assessment of Caring Activities (MACA-YC18) ***(8-17 years)*** [32] is an 18-item self-report measure that can be used to provide an index of the total amount of caring activity undertaken by the young person, as well as six subscale scores for domestic tasks, household management, personal care, emotional care, sibling care, and financial/practical care. The MACA-YC18 was designed as a short, easy to use, psychometric measure able to provide an index of the extent of caring activities that the young person is currently engaged in. Higher scores indicate greater levels of caring activity.[32 33]

Positive and Negative Outcomes of Caring (PANOC-YC20) ***(8-17 years)*** [32] a self-report measure that can be used to provide an index of positive and negative outcomes of caring. The PANOC-YC20 consists of two 10-item subscales: (1) positive responses, and (2) negative responses, which collectively assess the subjective cognitive and emotional impact of caring in young people with higher scores indicating greater positive and negative responses, respectively.

1. Pollack MM, Holubkov R, Funai T, et al. Pediatric intensive care outcomes: development of new morbidities during pediatric critical care. *Pediatr Crit Care Med* 2014;15(9):821-7. doi: 10.1097/PCC.0000000000000250 [published Online First: 2014/09/17]
2. Seid M, Limbers CA, Driscoll KA, et al. Reliability, validity, and responsiveness of the pediatric quality of life inventory (PedsQL) generic core scales and asthma symptoms scale in vulnerable children with asthma. *J Asthma* 2010;47(2):170-7. doi: 10.3109/02770900903533966 [published Online First: 2010/02/23]
3. Seid M, Varni JW, Gidwani P, et al. Problem-solving skills training for vulnerable families of children with persistent asthma: report of a randomized trial on health-related quality of life outcomes. *J Pediatr Psychol* 2010;35(10):1133-43. doi: 10.1093/jpepsy/jsp133 [published Online First: 2010/01/12]
4. Varni JW, Seid M, Knight TS, et al. The PedsQL 4.0 Generic Core Scales: sensitivity, responsiveness, and impact on clinical decision-making. *J Behav Med* 2002;25(2):175-93. doi: 10.1023/a:1014836921812 [published Online First: 2002/04/30]
5. Varni JW, Seid M, Smith Knight T, et al. The PedsQL in pediatric rheumatology: reliability, validity, and responsiveness of the Pediatric Quality of Life Inventory Generic Core Scales and Rheumatology Module. *Arthritis Rheum* 2002;46(3):714-25. doi: 10.1002/art.10095 [published Online First: 2002/03/29]
6. Mistry RD, Stevens MW, Gorelick MH. Health-related quality of life for pediatric emergency department febrile illnesses: an evaluation of the Pediatric Quality of Life Inventory 4.0 Generic Core Scales. *Health Qual Life Outcomes* 2009;7:5. doi: 10.1186/1477-7525-7-5 [published Online First: 2009/01/31]
7. Brandow AM, Brousseau DC, Pajewski NM, et al. Vaso-occlusive painful events in sickle cell disease: impact on child well-being. *Pediatr Blood Cancer* 2010;54(1):92-7. doi: 10.1002/pbc.22222 [published Online First: 2009/08/05]
8. Aspesberro F, Fesinmeyer MD, Zhou C, et al. Construct Validity and Responsiveness of the Pediatric Quality of Life Inventory 4.0 Generic Core Scales and Infant Scales in the PICU. *Pediatr Crit Care Med* 2016;17(6):e272-9. doi: 10.1097/PCC.0000000000000727 [published Online First: 2016/06/05]
9. Ong C, Lee JH, Leow MK, et al. Functional Outcomes and Physical Impairments in Pediatric Critical Care Survivors: A Scoping Review. *Pediatr Crit Care Med* 2016;17(5):e247-59. doi: 10.1097/PCC.0000000000000706 [published Online First: 2016/04/01]
10. Janssens L, Gorter JW, Ketelaar M, et al. Health-related quality-of-life measures for long-term follow-up in children after major trauma. *Quality of Life Research* 2008;17(5):701-13. doi: 10.1007/s11136-008-9339-0 [published Online First: 2008/04/26]
11. Eiser C, Morse R. The measurement of quality of life in children: past and future perspectives. *Journal of Developmental & Behavioral Pediatrics* 2001;22(4):248-56. [published Online First: 2001/09/04]
12. Varni JW, Burwinkle TM, Katz ER, et al. The PedsQL in pediatric cancer: reliability and validity of the Pediatric Quality of Life Inventory Generic Core Scales, Multidimensional Fatigue Scale, and Cancer Module. *Cancer* 2002;94(7):2090-106. doi: 10.1002/cncr.10428 [published Online First: 2002/04/05]
13. Aspesberro F, Mangione-Smith R, Zimmerman JJ. Health-related quality of life following pediatric critical illness. *Intensive Care Med* 2015;41(7):1235-46. doi: 10.1007/s00134-015-3780-7 [published Online First: 2015/04/09]
14. Pollack MM, Holubkov R, Glass P, et al. Functional Status Scale: new pediatric outcome measure. *Pediatrics* 2009;124(1):e18-28. doi: 10.1542/peds.2008-1987 [published Online First: 2009/07/01]

15. Pollack MM, Holubkov R, Funai T, et al. Simultaneous Prediction of New Morbidity, Mortality, and Survival Without New Morbidity From Pediatric Intensive Care: A New Paradigm for Outcomes Assessment. *Crit Care Med* 2015;43(8):1699-709. doi: 10.1097/CCM.0000000000001081 [published Online First: 2015/05/20]
16. Fiser DH, Long N, Roberson PK, et al. Relationship of pediatric overall performance category and pediatric cerebral performance category scores at pediatric intensive care unit discharge with outcome measures collected at hospital discharge and 1-and 6-month follow-up assessments. *Critical care medicine* 2000;28(7):2616-20.
17. Fiser DH, Tilford JM, Roberson PK. Relationship of illness severity and length of stay to functional outcomes in the pediatric intensive care unit: a multi-institutional study. *Critical care medicine* 2000;28(4):1173-79. [published Online First: 2000/05/16]
18. Fiser DH. Assessing the outcome of pediatric intensive care. *The Journal of pediatrics* 1992;121(1):68-74. [published Online First: 1992/07/01]
19. Fuchs S, Lewis RJ. Tools for the measurement of outcome after minor head injury in children: summary from the Ambulatory Pediatric Association/EMSC Outcomes Research Conference. *Academic emergency medicine* 2003;10(4):368-75. [published Online First: 2003/04/03]
20. Goodman R. Psychometric properties of the strengths and difficulties questionnaire. *Journal of the American Academy of Child & Adolescent Psychiatry* 2001;40(11):1337-45. doi: 10.1097/00004583-200111000-00015 [published Online First: 2001/11/09]
21. Goodman R. The Strengths and Difficulties Questionnaire: A Research Note. *Journal of Child Psychology and Psychiatry* 1997;38(5):581-86. doi: 10.1111/j.1469-7610.1997.tb01545.x [published Online First: 1997/07/01]
22. Yule W. Anxiety, depression and post-traumatic stress in childhood. . In: Sclare I, ed. Child psychology portfolio. Windsor, UK: NFER-Nelson 1997:35-38.
23. Colville G, Pierce C. Patterns of post-traumatic stress symptoms in families after paediatric intensive care. *Intensive Care Med* 2012;38(9):1523-31. doi: 10.1007/s00134-012-2612-2 [published Online First: 2012/06/19]
24. Perrin S, Meiser-Stedman R, Smith P. The Children's Revised Impact of Event Scale (CRIES): Validity as a screening instrument for PTSD. *Behavioural and Cognitive Psychotherapy* 2005;33(4):487-98.
25. Snyder CR, Hoza B, Pelham WE, et al. The Development and Validation of the Children's Hope Scale. *Journal of Pediatric Psychology* 1997;22(3):399-421. doi: 10.1093/jpepsy/22.3.399
26. Varni JW, Sherman SA, Burwinkle TM, et al. The PedsQL Family Impact Module: preliminary reliability and validity. *Health Qual Life Outcomes* 2004;2:55. doi: 10.1186/1477-7525-2-55 [published Online First: 2004/09/29]
27. Marteau TM, Bekker H. The development of a six-item short-form of the state scale of the Spielberger State—Trait Anxiety Inventory (STAI). *British Journal of Clinical Psychology* 1992;31(3):301-06. doi: 10.1111/j.2044-8260.1992.tb00997.x
28. Kroenke K, Spitzer RL, Williams JB, et al. An ultra-brief screening scale for anxiety and depression: the PHQ–4. *Psychosomatics* 2009;50(6):613-21.
29. Weathers FW, Litz BT, Keane TM, et al. The PTSD checklist for DSM-5 (PCL-5). US: Department of Veteran Affairs: National Center for PTSD 2013.
30. Blevins CA, Weathers FW, Davis MT, et al. The posttraumatic stress disorder checklist for DSM-5 (PCL-5): Development and initial psychometric evaluation. *Journal of traumatic stress* 2015;28(6):489-98. doi: 10.1002/jts.22059 [published Online First: 2015/11/26]

31. Bovin MJ, Marx BP, Weathers FW, et al. Psychometric properties of the PTSD Checklist for Diagnostic and Statistical Manual of Mental Disorders–Fifth Edition (PCL-5) in veterans. *Psychological Assessment* 2016;28(11):1379.
32. Joseph S, Becker F, Becker S. Manual for measures of caring activities and outcomes for children and young people. Nottingham: The University of Nottingham 2009.
33. Joseph S, Becker S, Becker F, et al. Assessment of caring and its effects in young people: development of the Multidimensional Assessment of Caring Activities Checklist (MACA-YC18) and the Positive and Negative Outcomes of Caring Questionnaire (PANOC-YC20) for young carers. *Child: care, health and development* 2009;35(4):510-20. doi: 10.1111/j.1365-2214.2009.00959.x [published Online First: 2009/03/27]
