# Supplementary material for: Study protocol for a multicentre longitudinal mixed methods study to explore the Outcomes of ChildrEn and fAmilies in the first year after paediatric Intensive Care: the OCEANIC study
Source: BMJ Open. 2020 May 17;10(5):e038974. doi: 10.1136/bmjopen-2020-038974 (PMC7239532; doi:10.1136/bmjopen-2020-038974)
Supplement: Author's manuscript [file bmjopen-2020-038974.draft_revisions.pdf]

BMJ Open

BMJ Open is committed to open peer review. As part of this commitment we make the peer review history of every article we publish publicly available.

When an article is published we post the peer reviewers' comments and the authors' responses online. We also post the versions of the paper that were used during peer review. These are the versions that the peer review comments apply to.

The versions of the paper that follow are the versions that were submitted during the peer review process. They are not the versions of record or the final published versions. They should not be cited or distributed as the published version of this manuscript.

BMJ Open is an open access journal and the full, final, typeset and author-corrected version of record of the manuscript is available on our site with no access controls, subscription charges or pay-per-view fees (http://bmjopen.bmj.com).

If you have any questions on BMJ Open's open peer review process please email
info.bmjopen@bmj.com

BMJ Open

A study protocol for a multi-centre longitudinal mixed methods study to explore the Outcomes of ChildrEn and fAmilies in the first year after paediatric Intensive Care: The OCEANIC Study

Journal:	*BMJ Open*
Manuscript ID	bmjopen-2020-038974
Article Type:	Protocol
Date Submitted by the Author:	30-Mar-2020
Complete List of Authors:	Manning, Joseph; University of Nottingham, Children and Young People Health Research, School of Health Sciences; Nottingham University Hospitals NHS Trust, Nottingham Children's Hospital<br>Latour, Jos; University of Plymouth School of Nursing and Midwifery; Hunan Children's Hospital, Nursing Department<br>Curley, Martha ; University of Pennsylvania, Department of Family and Community Health, School of Nursing ; University of Pennsylvania Perelman School of Medicine, Anesthesia and Critical Care Medicine<br>Draper, Elizabeth; University of Leicester, Department of Health Sciences<br>Jilani, Tahseen; University of Nottingham, Advanced Data Analysis Centre (ADAC); University of Nottingham, Health Data Research UK<br>Quinlan, Philip; University of Nottingham, Advanced Data Analysis Centre (ADAC); University of Nottingham, Health Data Research UK<br>Watson, R; University of Washington, Division of Pediatric Critical Care Medicine, Department of Pediatrics; Seattle Children's Research Institute, Centre for Child Health, Behaviour, and Development<br>Rennick, Janet; McGill University Faculty of Medicine, Ingram School of Nursing; Research Institute of the McGill University Health Centre, Centre for Outcomes Research & Evaluation<br>Colville, Gillian; St Georges University Hospitals NHS Foundation Trust, Paediatric Psychology Service; University of London St George's, Population Health Research Institute<br>Pinto, Neethi; University of Chicago, Section of Pediatric Critical Care, Department of Pediatrics<br>Latif, Asam; University of Nottingham, School of Health Sciences<br>Popejoy, Emma; University of Nottingham, Children and Young People Health Research, School of Health Sciences; Nottingham University Hospitals NHS Trust, Nottingham Children's Hospital<br>Coad, Jane; University of Nottingham, Children and Young People Health Research, School of Health Sciences
Keywords:	Paediatric intensive & critical care < ANAESTHETICS, STATISTICS & RESEARCH METHODS, QUALITATIVE RESEARCH

For peer review only - http://bmjopen.bmj.com/site/about/guidelines.xhtml

I, the Submitting Author has the right to grant and does grant on behalf of all authors of the Work (as defined in the below author licence), an exclusive licence and/or a non-exclusive licence for contributions from authors who are: i) UK Crown employees; ii) where BMJ has agreed a CC-BY licence shall apply, and/or iii) in accordance with the terms applicable for US Federal Government officers or employees acting as part of their official duties; on a worldwide, perpetual, irrevocable, royalty-free basis to BMJ Publishing Group Ltd ("BMJ") its licensees and where the relevant Journal is co-owned by BMJ to the co-owners of the Journal, to publish the Work in this journal and any other BMJ products and to exploit all rights, as set out in our licence.

The Submitting Author accepts and understands that any supply made under these terms is made by BMJ to the Submitting Author unless you are acting as an employee on behalf of your employer or a postgraduate student of an affiliated institution which is paying any applicable article publishing charge ("APC") for Open Access articles. Where the Submitting Author wishes to make the Work available on an Open Access basis (and intends to pay the relevant APC), the terms of reuse of such Open Access shall be governed by a Creative Commons licence – details of these licences and which Creative Commons licence will apply to this Work are set out in our licence referred to above.

Other than as permitted in any relevant BMJ Author's Self Archiving Policies, I confirm this Work has not been accepted for publication elsewhere, is not being considered for publication elsewhere and does not duplicate material already published. I confirm all authors consent to publication of this Work and authorise the granting of this licence.

For peer review only

A study protocol for a multi-centre longitudinal mixed methods study to explore the Outcomes of ChildrEn and fAmilies in the first year after paediatric Intensive Care: The OCEANIC Study

Authors and Affiliations

Joseph C. Manning, RN, PhD [1,2,10], Jos M. Latour, RN, PhD [3,4], Martha A.Q. Curley, RN, PhD[5,6,7], Elizabeth S. Draper, PhD[8], Tahseen Jilani, PhD[9,10], Philip R. Quinlan, PhD[9,10], R. Scott Watson, MD, MPH[11,12], Janet E. Rennick, RN, PhD[13,14,15], Gillian Colville, MPhil, CPsychol[16,17], Neethi Pinto, MD, MS[18], Asam Latif, MGPhC, PhD[2], Emma Popejoy, RN, PhD[1,2], Jane Coad, RN, PhD[2], *for the OCEANIC Study Investigators*

[1]Nottingham Children's Hospital, Nottingham University Hospitals NHS Trust, Nottingham, UK.

[2]Children and Young People Health Research, School of Health Sciences, Faculty of Medicine and Health Sciences, University of Nottingham, Nottingham, UK.

[3]School of Nursing and Midwifery, Faculty of Health, University of Plymouth, UK.

[4]Nursing Department, Hunan Children's Hospital, Changsha, People's Republic of China.

[5]Department of Family and Community Health, School of Nursing, University of Pennsylvania, Philadelphia, PA, USA

[6]Anesthesia and Critical Care Medicine, Perelman School of Medicine, University of Pennsylvania, Philadelphia, PA, USA

[7]The Research Institute, Children's Hospital of Philadelphia, Philadelphia, PA, USA

[8]Department of Health Sciences, College of Life Sciences, University of Leicester, UK.

[9]Advanced Data Analysis Centre, The University of Nottingham, Nottingham, UK.

[10]Health Data Research UK, The University of Nottingham, Nottingham, UK.

[11]Division of Pediatric Critical Care Medicine, Department of Pediatrics, University of Washington, Seattle, WA, USA.

For peer review only - http://bmjopen.bmj.com/site/about/guidelines.xhtml

[12]Centre for Child Health, Behaviour, and Development, Seattle Children's Research Institute, Seattle, WA, USA

[13]Ingram School of Nursing, Faculty of Medicine, McGill University, Montreal, Quebec, Canada

[14]The Montreal Children's Hospital, McGill University Health Centre, Montreal, Quebec, Canada

[15]Centre for Outcomes Research & Evaluation, Research Institute of the McGill University Health Centre, Montreal, Quebec, Canada

[16]Paediatric Psychology Service, St George's University Hospitals NHS Foundation Trust, London, UK.

[17]Population Health Research Institute, St George's University of London, London, UK.

[18]Section of Pediatric Critical Care, Department of Pediatrics, University of Chicago, Chicago, IL, USA.

Corresponding Author:

Dr Joseph C. Manning, Clinical Associate Professor / NIHR HEE ICA Clinical Lecturer, SC3084, C-floor, East Block, QMC Campus, Derby Road, Nottingham. NG7 2UH. joseph.manning@nottingham.ac.uk.

Clinical Trials Registration Number: ISRCTN 28072812

Keywords: Pediatric Intensive Care Unit, Post Intensive Care Syndrome-pediatrics, Child, Parent, Sibling, Outcome

Running Header: The OCEANIC Study Protocol

Word Count: 4353 words

For peer review only - http://bmjopen.bmj.com/site/about/guidelines.xhtml

For peer review only

ABSTRACT

Introduction: Annually in the UK 20,000 children become very ill or injured and need specialist care within a Paediatric Intensive Care Unit (PICU). Most children survive. However, some children and their families may experience problems after they have left the PICU including physical, functional, and/or emotional problems. It is unknown which children and families experience such problems, when these occur or what causes them. The aim of this mixed-method longitudinal cohort study is to understand the physical, functional, emotional and social impact of children surviving PICU (aged: 1 month-17 years), their parents and siblings, during the first-year after a PICU admission.

Methods and analysis: A quantitative study involving 300 child survivors of PICU; 300 parents; and 150-300 siblings will collect data (using self-completion questionnaires) at baseline, PICU discharge, 1, 3, 6 and 12 months post-PICU discharge. Questionnaires will comprise of validated and reliable instruments. Demographic data, PICU admission and treatment data, health related quality of life, functional status, strengths and difficulties behaviour and post-traumatic stress symptoms will be collected from the child. Parent and sibling data will be collected on the impact of paediatric health conditions on the family's functioning capabilities, levels of anxiety and social impact of the child's PICU admission. Data will be analysed using descriptive and inferential statistics. Concurrently, an embedded qualitative study involving semi-structured interviews with 24 enrolled families at 3 months and 9 months post-PICU discharge will be undertaken. Framework analysis will be used to analyse the qualitative data.

Ethics and dissemination: The study has received ethical approval from the National Health Services Research Ethics Committee [Ref: 19/WM/0290] and full governance clearance. This will be the first UK study to comprehensively investigate physical, functional, emotional and social consequences of PICU survival in the first year post-discharge.

For peer review only

For peer review only - http://bmjopen.bmj.com/site/about/guidelines.xhtml

Article Summary

Strengths and limitations of this study

- The OCEANIC study will be the first multisite, comprehensive study conducted in the UK to investigate the physical, functional, emotional and social consequences of PICU survival in the first year post-discharge.
- Our longitudinal study design will allow us to look at changes over time in the same patient/family, providing insights into the temporal sequence of changes that may occur as a result of childhood critical illness/injury.
- The qualitative study (interviews with children, parents and siblings) will be analysed in conjunction with quantitative data allowing a fuller understanding of physical, functional, emotional and social consequences of being on PICU and any outstanding needs.
- The primary limitation of this study is loss to follow-up and missing data points that would challenge the internal validity of reported results from The OCEANIC study.

For peer review only

For peer review only - http://bmjopen.bmj.com/site/about/guidelines.xhtml

INTRODUCTION

In the United Kingdom (UK) annually, approximately 20,000 children (aged 0-18 years) experience a critical illness, requiring paediatric intensive care unit (PICU) treatment and care.[1] Despite increasing demand on paediatric critical care services, PICU survival has increased substantially over the past three decades, rendering mortality alone an insufficient metric for outcomes assessment post-PICU discharge.[2] Over 96% of children admitted to PICU survive.[1] However, the decline in mortality has been accompanied by a concomitant increase in morbidity.[3] Evidence is building which portrays a cohort of PICU survivors who are physically deconditioned, cognitively impaired, and emotionally distraught. The emotional and social health of the PICU survivor's parents and siblings may also be affected.[4, 5]

Two systematic reviews reported that approximately 25% of critically ill children exhibited negative psychological and behavioural responses within the first-year post-discharge.[6, 7] Similar themes were identified in a systematic review of qualitative studies examining the psychosocial impact of PICU hospitalization on children,[8] lending support to the importance in identifying children suffering from psychological sequelae. Given that psychological well-being is shaped by multiple factors, alterations in the child's sense of self and interpersonal relationships, as well as ongoing worries and fears about hospitalization, have the potential to affect recovery during the early post-discharge period, and during critical periods of growth and development. Health related quality of life (HRQOL) studies identify deterioration in the emotional well-being of 20-30% of children up to 1-year post-PICU discharge, [6, 7] suggesting a sustained effect.

The impact of a child's critical illness on family members may be profound as they, too, can experience psychosocial sequelae.[5, 9] Family members' responses may, in turn, influence the outcomes of child survivors following paediatric critical illness. Furthermore there is evidence that critical illness impacts a family's social functioning in relation to re-integration with peers; the child and family's social capital; and the economic impact of unemployment on families when a care-giver has to relinquish work responsibilities to care for a child.[10] However, the interplay between the child, their parent and siblings' outcomes, caregiver roles, and family needs, and how these change over time, are largely absent in the literature.

Globally [11-13] and in the UK [14, 15] researchers, clinicians, and patients and their families have recognised understanding and supporting adult survivors of intensive care is both a

For peer review only - http://bmjopen.bmj.com/site/about/guidelines.xhtml

research and clinical priority. Patient and public consultation conducted with the PICU community (including children, their families, service providers and commissioners) confirms that understanding and optimising the outcomes of children and their families is also a research priority for childhood survivors of PICU [16]

METHODS AND ANALYSIS

Study purpose and objectives

The purpose of the OCEANIC study is to explore child PICU survivors' health outcomes and family impact over one-year post-PICU discharge. In order to identify morbidities, when they occur, and whether there are factors that could be modified to improve the health and well-being of PICU survivors and their families.

OCEANIC has four specific objectives:

1. To describe the physical, cognitive, emotional, and social health outcomes and trajectory of recovery in children post-PICU discharge.
2. To determine the baseline and PICU factors associated with impaired outcomes.
3. To explore the longitudinal emotional and social health outcomes of parents and siblings.
4. To ascertain the care and support needs of children and their parents and siblings.

Theoretical Framework

Based upon a state-of-the-science review of post-discharge outcomes in paediatric critical care [17], a conceptual framework describing the constellation of potential physical, cognitive, emotional, and social health effects that may be uniquely experienced by children and families who survive paediatric critical illness has been proposed (**Error! Reference source not found.**)[18]. This framework incorporates the importance of pre-existing health status, sociodemographic data, physiologic maturation, and psychosocial development on the trajectory of health recovery over a child's lifetime. Additionally, the framework recognizes that the interdependence of the child and family is central to understanding the long-term multidimensional sequelae of paediatric critical illness. This framework provides a roadmap

For peer review only - http://bmjopen.bmj.com/site/about/guidelines.xhtml

for understanding longitudinal outcomes; the proposed study will organize data collection using this framework.

This embedded mixed-methods study involves two linked work-packages (overview presented in Figure 2). The first work-package will be a quantitative study involving 300 child survivors of critical illness; 300 parents; and 150-300 siblings. The second work-package will be a qualitative interview study of two cohorts of 12 families, at 3 and 9 months post PICU discharge. Mixing will occur through the sampling and selection of participants for the embedded qualitative study from those enrolled in the quantitative study, as well as in the framework analysis.

Quantitative study

Data regarding the PICU admission of each child participant will be downloaded from the Paediatric Intensive Care Audit Network (PICANet) database, a secure and confidential high quality clinical database of paediatric intensive care activity in the UK and Ireland. Data extracted will include: demographic and socioeconomic data; pre-PICU health status; and acute illness data (PICU admission and discharge diagnoses; co-morbidities; operations and invasive procedures performed; type of admission (planned/unplanned); PICU and hospital length of stay, duration of mechanical ventilation, high frequency oscillatory ventilation, extracorporeal membrane oxygenation, renal replacement therapy, and vasopressor/inotropic support; sedative medications and days of exposure). Outcome data will also be collected from each child (or proxy), their parent, and sibling (if appropriate) prospectively over the first-year post-PICU discharge.

Study measures

Currently there are no standardised or agreed set of outcome measures for research with the PICU patient population. Therefore, the outcome measures used in this study were selected for their validity, reliability, ease of use, availability in electronic versions and previous use with the population under investigation. Furthermore, the focus and selection of these measures was informed by the Post Intensive Care Syndrome in pediatrics (PICS-p) framework, contemporary literature, and consultation with patients, public, and PICU clinicians. In line with feedback from patient and public involvement (PPI) consultations, outcomes will be

For peer review only - http://bmjopen.bmj.com/site/about/guidelines.xhtml
For peer review only

collected at six time-points: Baseline status (pre-PICU discharge); at PICU discharge; 1, 3, 6 and 12 months post-PICU discharge. The outcomes measured and time points are outlined in Table 1.

Data collection measures, versions, and report format according to age and study participant (child PICU survivor, parent/legal guardian or sibling) are reported in Table 2. A brief overview of the measures is provided in *Supplementary File 1*.

Child related measures include:

- PedsQL™ 4.0 (Pediatric Quality of Life Inventory) Generic Core Scales *(2-17 years)* and Infant Scales *(1-23 months)* – Acute Version [3, 19-28]
- PedsQL™ Multi-dimensional Fatigue Scale *(2-17 years)* – Acute Version [29]
- PedsQL™ Pediatric Pain Questionnaire *(5-17 years)*
- Functional Status Scale (FSS) *(1 month-17 years)* [30-32]
- Pediatric Cerebral Performance Category (PCPC) and the Pediatric Overall Performance Category (POPC) *(1 month – 17 years)* [33-36]
- Strengths and Difficulties Questionnaire (SDQ) *(2-17 years)* [37, 38]
- Child Revised Impact of Events Scale (CRIES-8) *(7-17 years)* [39-41]
- Children's Hope Scale (CHS) *(8-17 years)* [42]

Parent related measures

- PedsQL™ Family Impact Module (FIM) Version 2.0 [43]
- State-Trait Anxiety Inventory 6 (STAI-6) [44]
- Patient Health Questionnaire-4 (PHQ-4) [45]
- The Post Traumatic Stress Disorder (PTSD) Checklist for DSM-5 (PCL-5)[46-48].

Sibling related measures

- PedsQL™ 4.0 (Pediatric Quality of Life Inventory) Generic Core Scales *(2-17 years)* [3, 19-28]
- Children's Hope Scale (CHS) *(8-17 years)* [42]
- Multidimensional Assessment of Caring Activities (MACA-YC18) *(8-17 years)* [49, 50]
- Positive and Negative Outcomes of Caring (PANOC-YC20) *(8-17 years)* [50]

For peer review only - http://bmjopen.bmj.com/site/about/guidelines.xhtml

Qualitative study

The second work-package will be a qualitative study involving semi-structured interviews with 24 families, split between 3 and 9 months post-PICU discharge. As advocated in the child health literature, a pragmatic and participant-centred approach (based on choice, participation, and flexibility) to collecting qualitative data will be employed. Interviews will be conducted with children, parents/legal guardians, and siblings either collectively or separately. Interviews will take place at the participants' preferred time and method (e.g. face-to-face, telephone). The use of multiple sources of data will provide contextualised, converging and emerging lines of inquiry.

Sample and recruitment

Setting

Participants will be recruited from at least five PICUs across England chosen to include variation in unit size, case mix, geographical location, and patient demographic.

Eligibility criteria

Participants for this study include: (1) PICU child survivors, (2) parents/legal guardians and (3) siblings:

1. PICU child survivor: (a) Aged 1 month (and ≥44 weeks corrected gestational age) to 17 years at the point of PICU admission; (b) will be discharged from the PICU in next 48 hours; (c) PICU total length of stay (LOS) ≥ 72 hours at point of discharge in which the patient received PICU therapies for organ dysfunction; (d) At least one parent/legal guardian (≥18 years of age or considered emancipated) living with the potential subject.

2. Parent: (a) parent or legal guardian; (b) cohabits with the child.

3. Siblings: (a) aged ≥ 8 years (at baseline); (b) is a sibling of the children PICU survivor; (c) cohabits with the child PICU survivor for at least 50% of the time; (d) can independently self-report.

Sample

For peer review only - http://bmjopen.bmj.com/site/about/guidelines.xhtml

Sample size

Quantitative study: We anticipate enrolling 300 children (and their families) from five PICUs in equal proportions (60 per centre) over a 6-month period. Based on previous PICU studies [51, 52], we conservatively estimate a 20% attrition rate over one year. Thus, we anticipate having one-year outcomes for 240 patients at the end of the study. With 240 participants, we will have high power to detect small/moderate correlations between early PedsQL™ measurements (to assess the trajectory of recovery) and other baseline and PICU factors with one-year PedsQL™ summary scores. Using a two-sided 0.05 level test, we have 80% power to detect correlations of 0.18 or larger in magnitude. With 240 participants, we will also have high power to detect moderate differences when comparing two groups using a t-test (e.g., comparison of PedsQL™ summary scores by gender or diagnosis category). In addition, many of the analyses will involve multiple linear regression modelling to adjust for baseline factors or confounding variables. With 240 participants, there is high power for the assessment of modest covariate effects with linear regression. Thus, we anticipate having high power for assessing correlations or linear regression effects as well as for comparing groups with our expected one-year sample size.

Qualitative study: A stratified sample of up to 24 families (which may include the child, parent and sibling, with a maximum of 72 participants in total) will be enrolled into the qualitative interviews. This sample size will capture diverse perspectives around support needs and is expected to achieve data saturation in the qualitative analysis [53].

Sampling technique

Quantitative study sampling technique: A consecutive sampling strategy will be employed [54]. Each site will screen daily over a 12-month period and invite all eligible children to participate in the study. Data from screening logs, including refusal to participate and admission numbers at each site, will be collected and used to contextualise the reporting of the analysis. In order to recruit a sample that is representative of the PICU populous, a sampling frame based on age and diagnosis reported from PICANet data [1] will be used. This frame will be used to guide the recruitment of participants recruited into the study and is outlined in Table 3.

For peer review only - http://bmjopen.bmj.com/site/about/guidelines.xhtml

Qualitative study sampling technique: Two cohorts of 12 families (including the child, parent and a sibling) will be selected using a stratified sampling approach based on the child's PedsQL^{TM} score at 1 month post-PICU discharge and 6 months post-PICU discharge. Stratification using previously reported norms for PedsQL^{TM} as well as variation in relation to geographical locality, PICU presenting condition, age and ethnicity will be sought.

Study procedures

Quantitative study

Over a six-month period, each site will screen daily the children admitted to PICU and invite all eligible children to participate in the study. Site investigators (or their designated nominee) who are part of the PICU clinical care team will determine eligibility.

In line with feedback from PPI work in the development of this study, each participant (aged ≥ 5 years) will be provided with a single £15 gift voucher as a token of appreciation for participating in the study. Vouchers will be provided to all participants on the completion of the study data collection period (T6- 12 month's post-PICU discharge).

Qualitative study

For the qualitative study, participants will be identified from PedsQL^{TM} scores of the child participant at 1 month post-PICU discharge and 6 months post-PICU discharge. The identification and recruitment process is summarised in Figure 3 and will follow a systematic process:

1. Child participant PedsQL^{TM} scores will be collected and submitted by sites onto REDCap Cloud.

2. The Chief Investigator will review the scores and stratify the sample based whether the PedsQL^{TM} score is within 1, 2 or >2 standard deviations from the published norms, selecting at least 4 children for each group at 1 month post-PICU discharge and 6 months post-PICU discharge. To maximise diversity in families (child, parent and sibling) interviewed, where possible participants will be selected based on geographical locality, PICU presenting condition, age and ethnicity.

For peer review only - http://bmjopen.bmj.com/site/about/guidelines.xhtml

3. The study ID of potential participants will be sent to sites, who will then contact the family directly, requesting consent to receive contact from the Chief Investigator/study researcher.

4. The Chief Investigator/study researcher will contact families that have agreed to being contacted, to consent for qualitative interviews and to arrange suitable date, time and location.

Analyses

Quantitative study data analysis

Descriptive statistics will be presented for demographic information, and past and current medical history. All child, parent, and sibling-related measures will be calculated, including means, standard deviations, medians, and interquartile ranges for continuous variables and frequency counts and percentages for categorical variables. Data will be examined for normality, outliers, and systematic missing data. Transformations will be undertaken as needed.

Analyses related to specific objectives include the following:

Objective 1: To describe the physical, cognitive, emotional, and social health outcomes and trajectory of recovery in children post-PICU discharge. The primary aim is to explore child PICU survivors' health outcomes and trajectory of recovery over the first year post-PICU discharge. PICU survivors' health outcomes will be compared with published population means from the general and chronically ill populations using t-tests or Mann-Whitney test as appropriate. For the longitudinal data, correlations will be assessed between time points using Spearman correlations and a linear mixed regression model with random subject effects will be used to analyse trajectories over time. In case of lack of normality, the non-parametric longitudinal approach (nparLD) will be implemented.

Objective 2: To determine the baseline and PICU factors associated with impaired outcomes. To identify factors associated with impaired health outcomes among PICU survivors, correlation analyses followed by Principle Component Analysis (PCA) will be applied to identify covariates for the regression modelling. For categorised recovery over one-year post-PICU discharge, mixed effect logistic regression will be applied. Variables will be entered using backward stepwise approach to control for collinearity. Model performance will be

For peer review only - http://bmjopen.bmj.com/site/about/guidelines.xhtml

assessed using sensitivity, specificity, positive predicted value, negative predicted value and AUCROC values. Bootstrapping through K-fold approach will be applied to ensure better modelling.

Objective 3: To explore the longitudinal emotional and social health outcomes of parents and siblings. Parent and sibling emotional- and social health outcomes will be compared to published means using t-tests or Mann-Whitney test as appropriate. PICU survivor and sibling PedsQL™ summary scores and SDQ scores will also be compared using paired t-tests or Wilcoxon Signed Rank test.

Graphical analyses will be performed to display the trajectories of health outcomes over time in our populations of critically ill children. Multiple linear and logistic regression methods will be used to explore the effects of primary diagnosis (e.g., respiratory, cardiovascular), PICU length of stay category, and site, to predict outcomes. We will explore whether adjustment for sex, race/ethnicity, or site affects study inferences through the use of mixed effects and generalized estimating equations models. Finally, we will also explore the use of classification and regression trees with recursive partitioning, principal component analysis, factor analysis, and machine learning methods to help describe subgroups of patients with similar trajectories of outcome.

Qualitative study data analysis

Audio recorded interview data will be transcribed verbatim with all participant identifiable information removed. Transcription will be conducted by a service approved by Nottingham University Hospitals NHS Trust Research and Innovation Department. Confidentiality agreements will be completed. Transcripts will be imported into NVivo 12, for sorting, coding, and categorising of the data.

Qualitative data will be analysed using the adapted five-stage Framework Analysis process to achieve *Objective 4*; identification of the care and support needs of children, their parents and siblings. The five stages of Framework Analysis comprise (1) familiarisation with the data through reading full transcripts; (2) development of a theoretical framework through identification of recurring and important themes; (3) indexing and pilot charting; (4) summarising data in an analytical framework; and (5) synthesising data by mapping and interpreting [55]. Stages 1-4 will be conducted separately for respondent type (children,

For peer review only

parents, or siblings) to enable specific care and support needs to be identified and summarised. Stage 5 will then allow for data to be compared and contrasted across the respondent groups (child, parent, sibling), child's PedsQL™ score (<1, 2, or >2 standard deviations from published norms), and time-points (1-3 months or 6-9 months post-PICU discharge).

Patient & Public Involvement

Underpinned by best principles of INVOLVE, children, young people (CYP) and families have been integral to the development of this study. In 2017, the Chief Investigator and Co-Investigator (Professor Latour) organised the UK's first symposium on aftercare and rehabilitation following PICU and engaged with over 60 PICU clinicians, an ex-PICU patient, and family members. Feedback identified that: a prospective longitudinal cohort study to further understand the outcomes for CYP and their families post-PICU was needed; and the collection of data at multiple time-points over the first year would have value for CYP and their families, health professionals, and research to direct the development of future interventions.

Further PPI has been undertaken with 11 parents (seven mothers and four fathers), four siblings (aged 9-13 years) and three CYP PICU survivors (aged 11-17 years) from the East and West Midlands. Participants' varied in ethnicity and family composition, and reasons for admissions to different PICUs. The proposed study was regarded as addressing an important topic. Respondents main concerns included: the potential to trigger negative reactions from participation; the collection of information pertaining to the pre-ICU state; and the difficulty of considering their own emotional wellbeing when their focus is on their child's survival. Suggestions to address these included: certificates and vouchers to thank participants, flexibility in the method of data collection, linking up with existing support services to build reminders, and removing reference to scores within the survey/s. Making the purpose of the research more visible through study website and social media would help parents' make decisions about participating and keeping updated with the study.

As part of this study we will continue to have meaningful advice and input from PPI. An advisory group has been assembled consisting of a young person that has been critically ill, parents and carers of children that have experienced critical illness/injury, and a sibling of a critical illness survivor. It is proposed that this group will have at least six-monthly meetings to ensure they have continued and active involvement in: the management of the research;

For peer review only - http://bmjopen.bmj.com/site/about/guidelines.xhtml

developing participant information resources; contributing to the study report; and dissemination of research findings.

ETHICS AND DISSEMINATION

Ethics

This research includes recruitment of seriously ill children on a PICU and a parent and sibling. It concerns a challenging topic requiring great skill and sensitivity in data collection. The study is being carried out by an experienced research team with clinical and research expertise in children and young people who are seriously ill. Research staff will have also received one-to-one protocol training with the CI. We will ensure the first approach is from a member of the child's usual care team, and is sensitive to the situation and status of the child.

PPI is central to this project and in ensuring that it remains grounded in the experiences of patients. The associated participant facing materials will be carefully developed (with age specific information sheets and consent/assent forms) and these will be reviewed by a PPI panel. The information sheets clearly state that discussing the experience of serious illness may be distressing, and we will ask participants to consider carefully how they feel about this prospect before deciding to take part.

Consent/assent

Eligible participants will be given at least 24 hours to consider whether they wish to participate in the study. It will be made clear to the parents that they will be free to withdraw their consent for their own and/or their child's participation in the study at any time without this having any impact on their child's care. The majority of children will be sedated and on a ventilator at recruitment, therefore will be unable to provide informed consent/assent.

For those children unable to provide consent/assent at the time of enrolment into the study, consent will be obtained from their parent/legal guardian. Efforts will be made to then consent/assent the child once they are able to (e.g. have the cognitive capacity) by the site teams. In the unlikely event that a child does not wish to participate (and the parent has consented for the child), the child's wishes will be upheld and the parent/sibling will be withdrawn from the study.

For peer review only - http://bmjopen.bmj.com/site/about/guidelines.xhtml

Interviews

We recognise that the discussing/recollecting a potentially difficult experience (the PICU admission) and any ongoing health and care needs may be upsetting for survivors/parents/families [10]. Therefore, all interviews will be conducted by the Chief Investigator or the OCEANIC Research Fellow, who both have previous experience of conducting interviews with children and families on sensitive issues. Interviews will be semi-structured over 30-60 minutes with appropriate breaks if necessary. Interviews will allow participants to explore any issues in-depth, which in itself may provide opportunity for issues, feelings and emotions to be discussed. This will be facilitated by creative/child centred data collection techniques that are sensitive to exploring potentially emotive events, in a constructive manner. Families will be given the choice whether they would like to have the interview separately (child, parent and sibling) or collectively.

It will be made clear to participants at the outset that the interview can be stopped at any time should they wish. Furthermore, if the child participant, their parent/legal guardian, or sibling becomes visibly upset during the interview, the investigator will:

1. Invite the parent/legal guardian (if present) to console the child/sibling, (if not already doing so)

2. Offer to temporarily stop or terminate the visit,

3. Respect the decision made by the participant to stop/carry on the interview.

All visits with children (<16 year olds) will be conducted with the parent/legal guardian present. In cases where it is not possible for parents to be present or the child specifically requests for them not to be present a second investigator from the study team will be present. All the study investigators have an enhanced Disclosure and Barring Service check. All investigators conducting the qualitative interviews are registered with Nursing and Midwifery Council (UK, first level) and are therefore bound by codes of professional conduct and have a professional obligation to share information with other agencies (i.e. social services), if an interview participant discloses information that relates to safeguarding or child protection.

For peer review only - http://bmjopen.bmj.com/site/about/guidelines.xhtml

Ethical review

The West Midlands – The Black Country NHS Research Ethics Committee has reviewed the study protocol and provided favourable opinion [Ref: 19/WM/0290]. The Health Research Authority has also approved the protocol [IRAS: 269642]. This study has been externally peer reviewed and awarded funding through a competitive process through the NIHR [ICA-CL-2018-04-ST2-009]. The study has been registered in International Standard Randomised Controlled Trials Number (ISRCTN) 28072812.

Dissemination

Despite advances to the evidence base, a comprehensive understanding of PICU morbidity among survivors after PICU-discharge remains limited. Historically, studies have focused on specific populations and/or diseases (such as prematurity, congenital heart disease, long-stay patients) rather than on issues experienced by the post-PICU discharge population as a whole.[31, 56-62] Moreover, these studies to date have examined variable outcomes (such as functional status, health-related qualify of life, psychological well-being, adaptive behaviours) at a single time point, [31, 56-62] with few studies considering the patient's pre-PICU status. Collectively, this heterogeneity in scope severely limits understanding of morbidities experienced by children who survive critical illness, and their trajectories.[26]

Whilst there is a definite need to understand the long-term outcome trajectories of children and families, the scope and purpose of this research is to address this critical gap by being the first study to provide a comprehensive and contemporary understanding of the outcomes of children and families in the first-year post-PICU admission. This will allow for health deficits across a spectrum of domains to be identified. It will provide a better understanding of those at risk of morbidity post-PICU admission, when this manifests, its natural history and any factors that could be modified to improve outcomes. Novel and contemporary insights into the outcomes of children and their family will be established through the study findings, which has been recognised as global priority area for PICU research. Moreover, this study will enhance understanding of the health outcomes of under researched groups within the PICU populous including those very young children (<2 years), as well as those with communication/developmental impairments. Collectively, characterization of the longitudinal recovery of children, their parents and siblings post-PICU discharge will allow interventions to be identified to prevent or mitigate morbidity and therefore have the potential to optimise

For peer review only - http://bmjopen.bmj.com/site/about/guidelines.xhtml

the outcomes and lives of children and their families. Findings will impact on the delivery and configuration of current services, as well as having the potential to inform the development of new models of care that improve the quality of services for patients and families.

The dissemination strategy will be multi-faceted to ensure findings are reported in a timely and relevant manner to key stakeholders that include patients and the public, health care professionals, commissioners and policy makers, and academics. Findings will be reported within a funder report (accessible through the NIHR Academy website), professional journals, and in high quality peer-reviewed, open-access journals. In addition, members of the PPI advisory group will assist in composing a summary which will be distributed to national parent support groups and charities. Key findings will also be posted on institutional websites and social media.

For peer review only

For peer review only - http://bmjopen.bmj.com/site/about/guidelines.xhtml

DATA STATEMENT

The datasets generated during the current study are not currently publicly available due to the study being ongoing. However, data will be available from the corresponding author on reasonable request once the study is complete. Furthermore, it is proposed that all data generated or analysed during the study will be included in published article (and their supplementary information files).

ACKNOWLEDGEMENTS

The OCEANIC study was adapted from the PICS-p Study protocol developed by MAQC, RSW, GC, JR, NP and JCM.

We would like to acknowledge Dr Julie Menzies (Nurse Researcher, Birmingham Children's Hospital) in supporting the Patient and Public Involvement activities undertaken as part of the development of the OCEANIC study protocol. Furthermore we would like to acknowledge Francois Arvin-Berod (Data Manager, Research and Innovation, Nottingham University Hospitals NHS Trust) who supported the development of the OCEANIC data capture and management through the REDCap Cloud.

AUTHOR CONTRIBUTIONS

JCM is the chief investigator for the OCEANIC study. JCM, JML, MAQC, ESD, TJ, PRQ, RSW, JER, GC, NP, and JC made a substantial contribution to the conceptualization and design of the study. JCM, AL and EP drafted the first version of the manuscript. All authors critically revised the manuscript for important intellectual content, gave approval of the final version to be published, and agreed to be accountable for all aspects of the work.

CONFLICT OF INTEREST STATEMENT

The authors have no conflicts of interest to disclose.

FUNDING

JCM is recipient of a National Institute for Health Research ICA Clinical Lectureship [ICA-CL-2018-04-ST2-009] which is funding this research study.

For peer review only - http://bmjopen.bmj.com/site/about/guidelines.xhtml

REFERENCES

1. PICANet. Annual Report: Summary Report. Leeds: Universities of Leeds and Leicester; 2017.

2. Namachivayam P, Shann F, Shekerdemian L, Taylor A, van Sloten I, Delzoppo C, et al. Three decades of pediatric intensive care: Who was admitted, what happened in intensive care, and what happened afterward. Pediatr Crit Care Med. 2010;11(5):549-55.

3. Pollack MM, Holubkov R, Funai T, Clark A, Berger JT, Meert K, et al. Pediatric intensive care outcomes: development of new morbidities during pediatric critical care. Pediatr Crit Care Med. 2014;15(9):821-7.

4. Shudy M, de Almeida ML, Ly S, Landon C, Groft S, Jenkins TL, et al. Impact of pediatric critical illness and injury on families: a systematic literature review. Pediatrics. 2006;118(Supplement 3):S203-S18.

5. Abela KM, Wardell D, Rozmus C, LoBiondo-Wood G. Impact of pediatric critical illness and injury on families: An updated systematic review. Journal of Pediatric Nursing. 2020;51:21-31.

6. Rennick JE, Rashotte J. Psychological outcomes in children following pediatric intensive care unit hospitalization: a systematic review of the research. J Child Health Care. 2009;13(2):128-49.

7. Davydow DS, Richardson LP, Zatzick DF, Katon WJ. Psychiatric morbidity in pediatric critical illness survivors: a comprehensive review of the literature. Arch Pediatr Adolesc Med. 2010;164(4):377-85.

8. Manning JC, Hemingway P, Redsell SA. Long-term psychosocial impact reported by childhood critical illness survivors: a systematic review. Nursing in critical care. 2014;19(3):145-56.

9. Nelson LP, Gold JI. Posttraumatic stress disorder in children and their parents following admission to the pediatric intensive care unit: A review. Pediatric Critical Care Medicine. 2012;13(3):338-47.

10. Manning JC, Hemingway P, Redsell SA. Stories of survival: Children's narratives of psychosocial well-being following paediatric critical illness or injury. J Child Health Care. 2017;21(3):236-52.

11. Needham DM, Davidson J, Cohen H, Hopkins RO, Weinert C, Wunsch H, et al. Improving long-term outcomes after discharge from intensive care unit: report from a stakeholders' conference. Crit Care Med. 2012;40(2):502-9.

12. Spragg RG, Bernard GR, Checkley W, Curtis JR, Gajic O, Guyatt G, et al. Beyond mortality: future clinical research in acute lung injury. American journal of respiratory and critical care medicine. 2010;181(10):1121-7.

For peer review only

For peer review only - http://bmjopen.bmj.com/site/about/guidelines.xhtml

13. Turnbull AE, Rabiee A, Davis WE, Nasser MF, Venna VR, Lolitha R, et al. Outcome measurement in ICU survivorship research from 1970-2013: a scoping review of 425 publications. Critical care medicine. 2016;44(7):1267.

14. Reay H, Arulkumaran N, Brett SJ. Priorities for future intensive care research in the UK: results of a James Lind alliance priority setting partnership. Journal of the Intensive Care Society. 2014;15(4):288-96.

15. National Institute for Health and Care Excellence. Rehabilitation after critical illness in adults. Quality Standard [QS158]. London NICE; 2017.

16. Manning JC, Hemingway P, Redsell SA. Survived so what? Identifying priorities for research with children and families post-paediatric intensive care unit. Nursing in Critical Care. 2017;23(2):68-74.

17. Watson RS, Choong K, Colville G, Crow S, Dervan LA, Hopkins RO, et al. Life after Critical Illness in Children—Toward an Understanding of Pediatric Post-intensive Care Syndrome. The Journal of Pediatrics. 2018;198:16-24.

18. Manning JC, Pinto NP, Rennick JE, Colville G, Curley MAQ. Conceptualizing Post Intensive Care Syndrome in Children-The PICS-p Framework. Pediatr Crit Care Med. 2018;19(4):298-300.

19. Seid M, Limbers CA, Driscoll KA, Opipari-Arrigan LA, Gelhard LR, Varni JW. Reliability, validity, and responsiveness of the pediatric quality of life inventory (PedsQL) generic core scales and asthma symptoms scale in vulnerable children with asthma. J Asthma. 2010;47(2):170-7.

20. Seid M, Varni JW, Gidwani P, Gelhard LR, Slymen DJ. Problem-solving skills training for vulnerable families of children with persistent asthma: report of a randomized trial on health-related quality of life outcomes. J Pediatr Psychol. 2010;35(10):1133-43.

21. Varni JW, Seid M, Knight TS, Uzark K, Szer IS. The PedsQL 4.0 Generic Core Scales: sensitivity, responsiveness, and impact on clinical decision-making. J Behav Med. 2002;25(2):175-93.

22. Varni JW, Seid M, Smith Knight T, Burwinkle T, Brown J, Szer IS. The PedsQL in pediatric rheumatology: reliability, validity, and responsiveness of the Pediatric Quality of Life Inventory Generic Core Scales and Rheumatology Module. Arthritis Rheum. 2002;46(3):714-25.

23. Mistry RD, Stevens MW, Gorelick MH. Health-related quality of life for pediatric emergency department febrile illnesses: an evaluation of the Pediatric Quality of Life Inventory 4.0 Generic Core Scales. Health Qual Life Outcomes. 2009;7:5.

24. Brandow AM, Brousseau DC, Pajewski NM, Panepinto JA. Vaso-occlusive painful events in sickle cell disease: impact on child well-being. Pediatr Blood Cancer. 2010;54(1):92-7.

25. Aspesberro F, Fesinmeyer MD, Zhou C, Zimmerman JJ, Mangione-Smith R. Construct Validity and Responsiveness of the Pediatric Quality of Life Inventory 4.0 Generic Core Scales and Infant Scales in the PICU. Pediatr Crit Care Med. 2016;17(6):e272-9.

For peer review only - http://bmjopen.bmj.com/site/about/guidelines.xhtml

26. Ong C, Lee JH, Leow MK, Puthucheary ZA. Functional Outcomes and Physical Impairments in Pediatric Critical Care Survivors: A Scoping Review. Pediatr Crit Care Med. 2016;17(5):e247-59.

27. Janssens L, Gorter JW, Ketelaar M, Kramer WL, Holtslag HR. Health-related quality-of-life measures for long-term follow-up in children after major trauma. Quality of Life Research. 2008;17(5):701-13.

28. Eiser C, Morse R. The measurement of quality of life in children: past and future perspectives. Journal of Developmental & Behavioral Pediatrics. 2001;22(4):248-56.

29. Varni JW, Burwinkle TM, Katz ER, Meeske K, Dickinson P. The PedsQL in pediatric cancer: reliability and validity of the Pediatric Quality of Life Inventory Generic Core Scales, Multidimensional Fatigue Scale, and Cancer Module. Cancer. 2002;94(7):2090-106.

30. Aspesberro F, Mangione-Smith R, Zimmerman JJ. Health-related quality of life following pediatric critical illness. Intensive Care Med. 2015;41(7):1235-46.

31. Pollack MM, Holubkov R, Glass P, Dean JM, Meert KL, Zimmerman J, et al. Functional Status Scale: new pediatric outcome measure. Pediatrics. 2009;124(1):e18-28.

32. Pollack MM, Holubkov R, Funai T, Berger JT, Clark AE, Meert K, et al. Simultaneous Prediction of New Morbidity, Mortality, and Survival Without New Morbidity From Pediatric Intensive Care: A New Paradigm for Outcomes Assessment. Crit Care Med. 2015;43(8):1699-709.

33. Fiser DH, Long N, Roberson PK, Hefley G, Zolten K, Brodie-Fowler M. Relationship of pediatric overall performance category and pediatric cerebral performance category scores at pediatric intensive care unit discharge with outcome measures collected at hospital discharge and 1-and 6-month follow-up assessments. Critical care medicine. 2000;28(7):2616-20.

34. Fiser DH, Tilford JM, Roberson PK. Relationship of illness severity and length of stay to functional outcomes in the pediatric intensive care unit: a multi-institutional study. Critical care medicine. 2000;28(4):1173-9.

35. Fiser DH. Assessing the outcome of pediatric intensive care. The Journal of pediatrics. 1992;121(1):68-74.

36. Fuchs S, Lewis RJ. Tools for the measurement of outcome after minor head injury in children: summary from the Ambulatory Pediatric Association/EMSC Outcomes Research Conference. Academic emergency medicine. 2003;10(4):368-75.

37. Goodman R. Psychometric properties of the strengths and difficulties questionnaire. Journal of the American Academy of Child & Adolescent Psychiatry. 2001;40(11):1337-45.

38. Goodman R. The Strengths and Difficulties Questionnaire: A Research Note. Journal of Child Psychology and Psychiatry. 1997;38(5):581-6.

39. Yule W. Anxiety, depression and post-traumatic stress in childhood. . In: Sclare I, editor. Child psychology portfolio. Windsor, UK: NFER-Nelson; 1997. p. 35-8.

For peer review only

For peer review only - http://bmjopen.bmj.com/site/about/guidelines.xhtml

40. Colville G, Pierce C. Patterns of post-traumatic stress symptoms in families after paediatric intensive care. Intensive Care Med. 2012;38(9):1523-31.

41. Perrin S, Meiser-Stedman R, Smith P. The Children's Revised Impact of Event Scale (CRIES): Validity as a screening instrument for PTSD. Behavioural and Cognitive Psychotherapy. 2005;33(4):487-98.

42. Snyder CR, Hoza B, Pelham WE, Rapoff M, Ware L, Danovsky M, et al. The Development and Validation of the Children's Hope Scale. Journal of Pediatric Psychology. 1997;22(3):399-421.

43. Varni JW, Sherman SA, Burwinkle TM, Dickinson PE, Dixon P. The PedsQL Family Impact Module: preliminary reliability and validity. Health Qual Life Outcomes. 2004;2:55.

44. Marteau TM, Bekker H. The development of a six-item short-form of the state scale of the Spielberger State—Trait Anxiety Inventory (STAI). British Journal of Clinical Psychology. 1992;31(3):301-6.

45. Kroenke K, Spitzer RL, Williams JB, Löwe B. An ultra-brief screening scale for anxiety and depression: the PHQ–4. Psychosomatics. 2009;50(6):613-21.

46. Weathers FW, Litz BT, Keane TM, Palmieri PA, Marx BP, Schnurr PP. The PTSD checklist for DSM-5 (PCL-5). US: Department of Veteran Affairs: National Center for PTSD 2013. Available from: https://www.ptsd.va.gov/professional/assessment/adult-sr/ptsd-checklist.asp.

47. Blevins CA, Weathers FW, Davis MT, Witte TK, Domino JL. The posttraumatic stress disorder checklist for DSM-5 (PCL-5): Development and initial psychometric evaluation. Journal of traumatic stress. 2015;28(6):489-98.

48. Bovin MJ, Marx BP, Weathers FW, Gallagher MW, Rodriguez P, Schnurr PP, et al. Psychometric properties of the PTSD Checklist for Diagnostic and Statistical Manual of Mental Disorders–Fifth Edition (PCL-5) in veterans. Psychological Assessment. 2016;28(11):1379.

49. Joseph S, Becker S, Becker F, Regel S. Assessment of caring and its effects in young people: development of the Multidimensional Assessment of Caring Activities Checklist (MACA-YC18) and the Positive and Negative Outcomes of Caring Questionnaire (PANOC-YC20) for young carers. Child: care, health and development. 2009;35(4):510-20.

50. Joseph S, Becker F, Becker S. Manual for measures of caring activities and outcomes for children and young people. Nottingham: The University of Nottingham; 2009.

51. Khetani MA, Albrecht EC, Jarvis JM, Pogorzelski D, Cheng E, Choong K. Determinants of change in home participation among critically ill children. Developmental Medicine & Child Neurology. 2018;60(8):793-800.

52. Rennick J, Dougherty G, Chambers C, Stremler R, Childerhose J, Stack D, et al. Children's psychological and behavioral responses following pediatric intensive care unit hospitalization: the caring intensively study. BMC Pediatrics. 2014;14(1):276.

53. Morse JM. Determining sample size Qualitative Health Research. 2000;10:3-5.

For peer review only - http://bmjopen.bmj.com/site/about/guidelines.xhtml

54. Daniel J. Sampling Essentials: Practical Guidelines for Making Sampling Choices. Thousand Oaks, California2012. Available from: http://methods.sagepub.com/book/sampling-essentials.

55. Ward DJ, Furber C, Tierney S, Swallow V. Using Framework Analysis in nursing research: a worked example. J Adv Nurs. 2013;69(11):2423-31.

56. Ambuehl J, Karrer A, Meer A, Riedel T, Schibler A. Quality of life of survivors of paediatric intensive care. Swiss Med Wkly. 2007;137(21-22):312-6.

57. Buysse CM, Raat H, Hazelzet JA, Hop WC, Maliepaard M, Joosten KF. Surviving meningococcal septic shock: health consequences and quality of life in children and their parents up to 2 years after pediatric intensive care unit discharge. Crit Care Med. 2008;36(2):596-602.

58. Knoester H, Bronner MB, Bos AP, Grootenhuis MA. Quality of life in children three and nine months after discharge from a paediatric intensive care unit: a prospective cohort study. Health Qual Life Outcomes. 2008;6:21.

59. Colville GA, Pierce CM. Children's self-reported quality of life after intensive care treatment. Pediatr Crit Care Med. 2013;14(2):e85-92.

60. Namachivayam SP, Alexander J, Slater A, Millar J, Erickson S, Tibballs J, et al. Five-Year Survival of Children With Chronic Critical Illness in Australia and New Zealand. Crit Care Med. 2015;43(9):1978-85.

61. Polic B, Mestrovic J, Markic J, Mestrovic M, Capkun V, Utrobicic I, et al. Long-term quality of life of patients treated in paediatric intensive care unit. Eur J Pediatr. 2013;172(1):85-90.

62. Choong K, Al-Harbi S, Siu K, Wong K, Cheng J, Baird B, et al. Functional recovery following critical illness in children: the "wee-cover" pilot study. Pediatr Crit Care Med. 2015;16(4):310-8.

For peer review only

For peer review only - http://bmjopen.bmj.com/site/about/guidelines.xhtml

FIGURE LEGENDS

Figure 1: Post Intensive Care Syndrome in pediatrics (PICS-p) framework; Manning et al., Pediatr Crit Care Med 2018; 19:298-300.

Figure 2: Overview of linked work packages of the OCEANIC study

Figure 3: Identification and recruitment of participants for Work Package 2- Qualitative Study

For peer review only

For peer review only - http://bmjopen.bmj.com/site/about/guidelines.xhtml

Table 1: Data collection measures and time points in which data is collected for child PICU survivor, parent/legal guardian and sibling

**Section 1: Child-survivor measures**	**Version**	**Items/ Time Required**	**T_0: Baseline (retrospective)**	**T_1: PICU Discharge**	**Post-PICU discharge** T_2: 1 month	T_3: 3 months	T_4: 6 months	T_5: 12 months
**1. Pediatric Quality of Life Inventory (PedsQL)™ Infant Scales Version 4.0 – Acute (Aged: 1-23 months)**<br><br>**<u>OR</u>**<br><br>**2. PedsQL™ Generic Core Scales Version 4.0 - Acute (Aged: 2 years+)**	Infant 1-12 months<br>Infant 13-23 months<br><br>Toddlers<br>Young Child<br>Child<br>Teen	36 items / <7min<br>45 items / <10 min<br><br>21 items / <5 min<br>23 items / <5 min<br>23 items / <5 min<br>23 items / <5 min	X	X	X	X	X	X
**3. PedsQL™ Multi-dimensional Fatigue Scale Version 3.0 - Acute**		18 items/ 5 min	X	X	X	X	X	X
**4. PedsQL™ Pediatric Pain Questionnaire (PPQ)™**		1 item / <1 min		X	X	X	X	X
**5. Functional Status Scale (FSS)**		6 items / 5 min	X	X	X	X	X	X
**6. Pediatric Overall Performance Category (POPC) and Pediatric Cerebral Performance Category (PCPC)**		2 item / 5 min	X	X	X	X	X	X
**7. Strengths and Difficulties Questionnaire (SDQ)**		25 items / 4 min		X	X	X	X	X
**8. Child Impact of Events Scale (CRIES-8)**		8 items / 4 minutes				X	X	X
**9. Children's Hope Scale (CHS)**		6 item / 3 minutes		X	X	X	X	X
**Max. total number of measures:**<br>*(NB for WP2 (Qualitative study) a sample of child survivors will take part in one semi-structured interview lasting approximately 30-60mins at either 1-3 months or 6-9 months post-discharge)*			**4**	7	7	**8**	8	**8**

For peer review only - http://bmjopen.bmj.com/site/about/guidelines.xhtml

				Post-PICU discharge			
**Section 2: Parent/legal guardian measures**	**Items/ Time Required**	**T_0: Baseline (retrospective)**	**T_1: PICU Discharge**	T_2: 1 month	T_3: 3 months	T_4: 6 months	T_5: 12 months
**1. PedsQL™ Family Impact Module Version 2.0**	36 items / 5 min		X	X	X	X	X
**2. State-Trait Anxiety Inventory (STAI: Y-6 item)**	6 items / 2 min		X	X	X	X	X
**3. Patient Health Questionnaire-4 (PHQ-4)**	4 items / 2 min		X	X	X	X	X
**4. PTSD Checklist (PCL)-5**	17 items / 5 min				X	X	X
**Total number of measures:** *(NB for WP2 (Qualitative study) a sample of parents will take part in one semi-structured interview lasting approximately 30-60mins at either 3 months or 9 months post-discharge)*		**-**	**3**	**3**	**4**	**4**	**4**
**Section 3: Sibling measures**							
**1. PedsQL™ Version 4.0 Generic Core Scales**	23 items / 4 min		X	X	X	X	X
**2. Strengths and Difficulties Questionnaire (SDQ)**	25 items / 4 min		X	X	X	X	X
**3. Multidimensional Assessment of Caring Activities (MACA-YC18)**	18 item / 2-4 min		X	X	X	X	X
**4. Positive and Negative Outcomes of Caring (PANOC-YC20)**	20 item / 2-4 min		X	X	X	X	X
**5. Children's Hope Scale (CHS)**	6 item / 3 minutes		X	X	X	X	X
**Total number of measures:** *(NB for WP2 (Qualitative study) a sample of siblings will take part in one semi-structured interview lasting approximately 30-60mins at either 3 months or 9 months post-discharge)*		**-**	5	**5**	**5**	5	5

For peer review only - http://bmjopen.bmj.com/site/about/guidelines.xhtml

For peer review only

Table 2: Data collection measures, versions, and report format according to age and study participant (child PICU survivor, parent/legal guardian or sibling)

**Section 1: Child PICU Survivor**							
	**PICU Survivor Participant Age**						
**Measure / Version (Reported by)**	1-12 months	13-23 months	2-4 years	5-7 years	8-10 years	11-12 years	13-17 years
**1. Pediatric Quality of Life Inventory (PedsQL)™ <u>Infant Scales</u> Version 4.0 - Acute**							
• Infants 1-12 months (Parent Reported)	X						
• Infants 13-24 months (Parent Reported)		X					
**2. PedsQL™ <u>Generic Core Scales</u> Version 4.0 - Acute**							
• Toddlers (Parent Reported)			X				
• Young Child (Child or Parent Reported)				X			
• Child (Child or Parent Reported)					X	X	
• Teen (Child or Parent Reported)							X
**3. PedsQL™ <u>Multi-dimensional Fatigue Scale</u> Version 3.0 - Acute**							
• Toddlers (Parent Reported)			X				
• Young Child (Child or Parent Reported)				X			
• Child (Child or Parent Reported)					X	X	
• Teen (Child or Parent Reported)							X
**4. PedsQL™ <u>Pediatric Pain Questionnaire</u> (PPQ)™**							
• Young Child (Child or Parent Reported)				X			
• Child (Child or Parent Reported)					X	X	
• Teen (Child or Parent Reported)							X
**5. Functional Status Scale (FSS)** (Parent Reported)	X	X	X	X	X	X	X
**6. Pediatric Cerebral Performance Category (PCPC) and Pediatric Overall Performance Category (POPC)** (Parent Reported)	X	X	X	X	X	X	X
**7. Strengths and Difficulties Questionnaire (SDQ)**							
• 2-4 year olds (Parent Reported)			X				
• 4-17 year olds (Parent Reported)				X	X		
• 11-17 year olds (Child Reported)						X	X
**8. Child Impact of Events Scale (CRIES-8)** (Child Reported)					X	X	X
**9. Children's Hope Scale (CHS)** (Child Reported)					X	X	X

For peer review only - http://bmjopen.bmj.com/site/about/guidelines.xhtml

**Section 2: Parent/Legal guardian**		
**Measure / Version (Reported by)**		**Parent/Legal guardian**
**1. Pediatric Quality of Life Inventory (PedsQL)™ Family Impact Module Version 2.0- Acute**	(Parent Reported)	X
**2. State-Trait Anxiety Inventory (STAI: Y-6 item)**	(Parent Reported)	X
**3. Patient Health Questionnaire-4 (PHQ-4)**	(Parent Reported)	X
**4. PTSD Checklist (PCL)-5**	(Parent Reported)	X

**Section 3: Sibling**				
**Measure / Version (Reported by)**		**Sibling Participant Age**		
		8-10 years	11-12 years	13-17 years
**1. Pediatric Quality of Life Inventory (PedsQL)™ Generic Core Scales Version 4.0- Acute**				
• Child	(Child Reported)	X	X	
• Teen	(Child Reported)			X
**2. Strengths and Difficulties Questionnaire (SDQ)**				
• 4-17 year olds	(Parent Reported)	X		
• 11-17 year olds	(Child Reported)		X	X
3. **Multidimensional Assessment of Caring Activities (MACA-YC18)**	(Child Reported)	X	X	X
**4. Positive and Negative Outcomes of Caring (PANOC-YC20)**	(Child Reported)	X	X	X
5. **Children's Hope Scale (CHS)**	(Child Reported)	X	X	X

For peer review only - http://bmjopen.bmj.com/site/about/guidelines.xhtml

Table 3: Proposed sampling frame for PICU survivor participant recruitment

	Diagnosis				
**Age (years)**	**Cardiovascular (28.1%)**	**Neurological (10.7%)**	**Respiratory (29.2%)**	**Other* (32%)**	**Total**
**0 (55%)**	47	19	48	53	167
**1-5 (25.2%)**	21	8	23	25	77
**6-10 (9.7%)**	8	3	8	9	28
**≥11 (10.3%)**	8	3	8	10	28
**Total**	84	33	87	63	300

*including: Blood/lymphatic; Body wall and cavities; Endocrine/metabolic; Trauma; Oncology; Musculo-skeletal; Multisystem; Infection; Gastrointestinal

For peer review only

Figure 1: Post Intensive Care Syndrome in pediatrics (PICS-p) framework; Manning et al., Pediatr Crit Care Med 2018; 19:298-300.

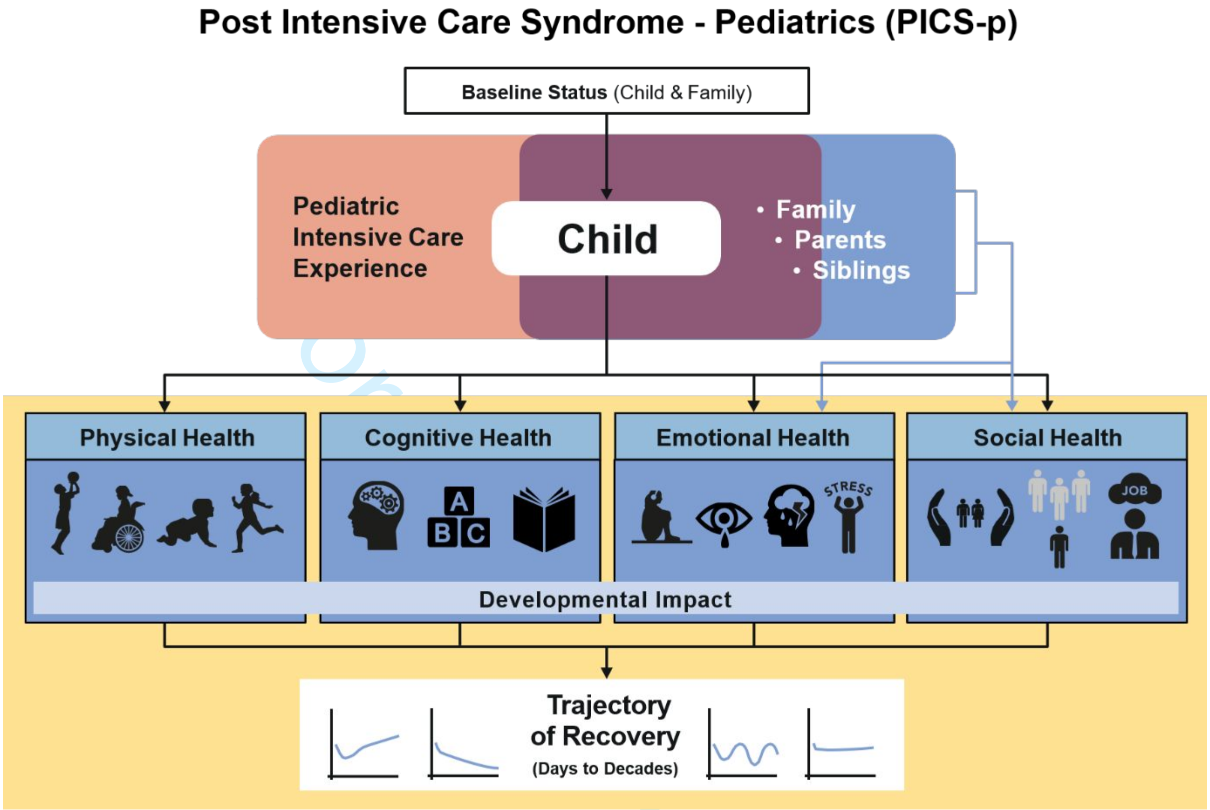

Figure 2: Overview of linked work packages of the OCEANIC study

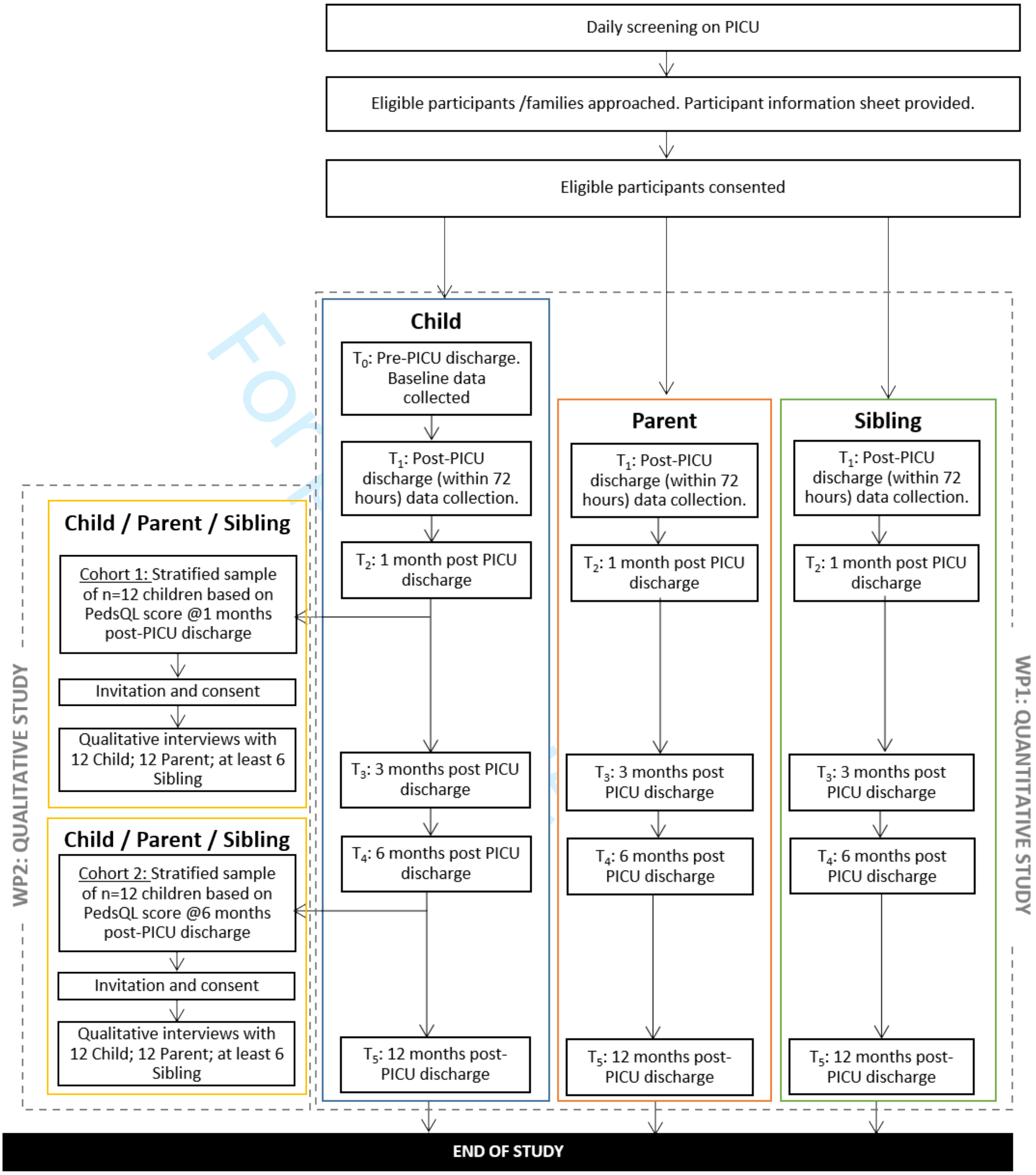

For peer review only

Figure 3: Identification and recruitment of participants for Work Package 2- Qualitative Study

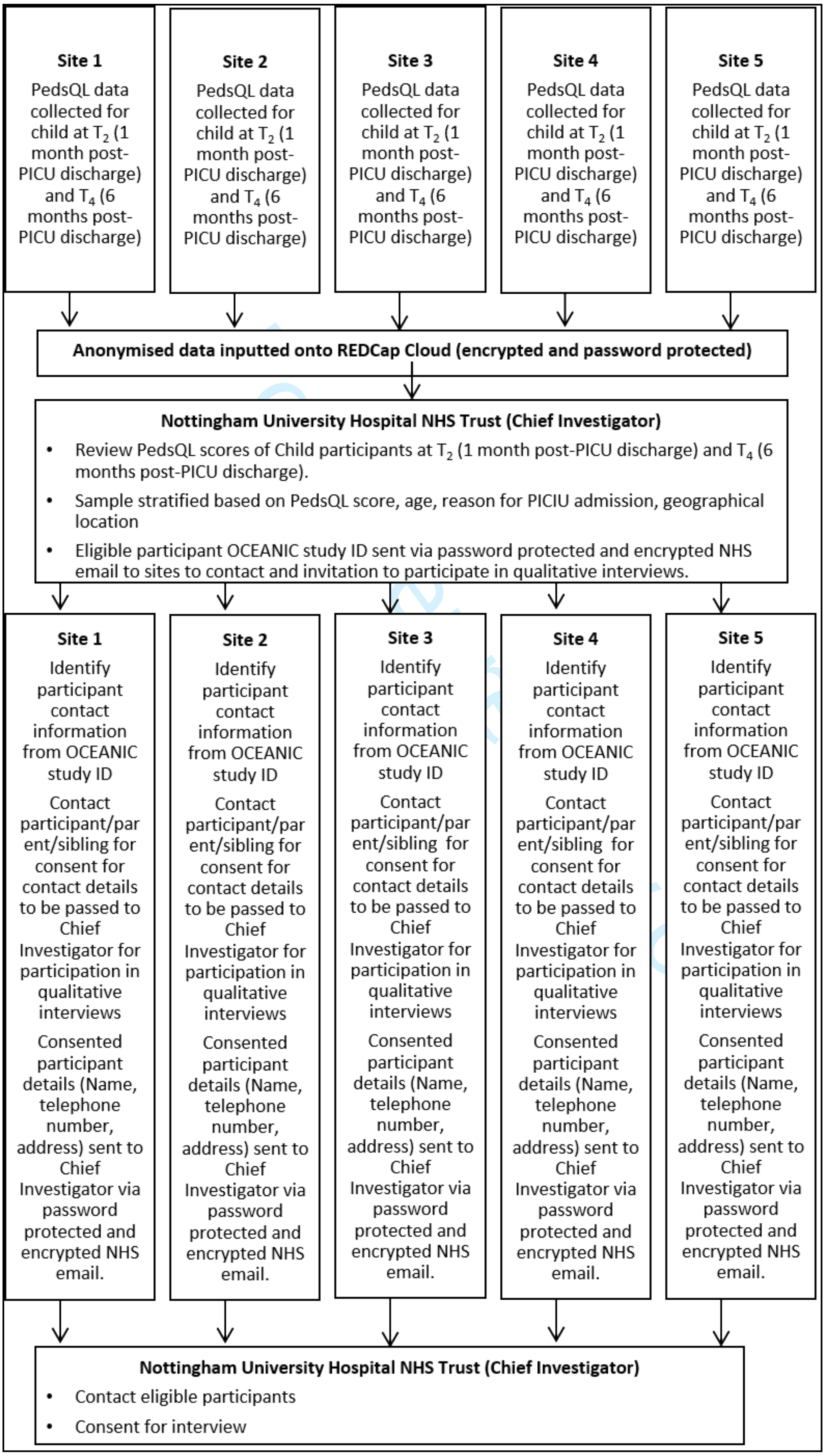

The OCEANIC study – Summary of measures used

Child related measures

PedsQL™ 4.0 (Pediatric Quality of Life Inventory) Generic Core Scales *(2-17 years)* and Infant Scales *(1-23 months)* – Acute Version measures HRQOL in children and adolescents aged 1 month to 17 years old. Both sets of instruments have good validity and reliability, have been widely used, [1-8] and can be completed in 5-7 minutes.[8-11] The instruments can discriminate between healthy children and those with a wide range of acute and chronic health conditions.

PedsQL™ Multi-dimensional Fatigue Scale *(2-17 years)* – Acute Version [12] is an 18-item scale that encompasses three domains: (1) General Fatigue, (2) Sleep/Rest Fatigue and (3) Cognitive Fatigue. The Multidimensional Fatigue Scale comprises parallel child self-report and parent proxy-report formats. Items for each of the forms are essentially identical, differing in developmentally appropriate language, or first or third person tense.

PedsQL™ Pediatric Pain Questionnaire *(5-17 years)* is a generic symptom-specific instrument to measure pain in patients with acute and chronic health conditions. We will use question #1 and #2, which asks participants capable of self-reporting to identify a point on a 100 mm line that best shows the worst pain the child experienced ‘now’ and ‘in the past week’. Anchors include “no hurting, no discomfort, or no pain” and “hurting a whole lot, very uncomfortable, severe pain”. A parent report version will be used for child participants that are unable to self-report.

Functional Status Scale (FSS) *(1 month-17 years)* is a valid and reliable assessment method to quantify functional status.[13 14] The FSS includes 6 domains: mental status, sensory functioning, communication, motor function, feeding, and respiratory. The FSS is amenable to studies of this nature due to ease of administration, granularity, and objectivity of assessment compared to other available methods and has been used in other outcome studies.[13 15]

Pediatric Cerebral Performance Category (PCPC) and the **Pediatric Overall Performance Category (POPC) *(1 month – 17 years)*** quantify short-term cognitive impairments and functional morbidity.[16 17] The POPC scale is dependent on the PCPC scale, as the PCPC status is included in POPC. Scores range from 1 to 6 for both scales with 1: good, 2: mild disability, and 6: brain death. Studies of patients with scores of 1–4 at PICU discharge, hospital discharge,

For peer review only

and one- and six-month follow-up show association with the Stanford Binet Intelligence Quotient, Bayley scales, and Vineland Adaptive Behaviour Scale. [16-19]

Strengths and Difficulties Questionnaire (SDQ) ***(2-17 years)*** [20 21] is a behavioural screening questionnaire used to evaluate emotional symptoms, conduct problems, hyperactivity/inattention, peer relationship problems, and prosocial behaviour. The SDQ quantifies low, medium, and high risk of emotional, behavioural, hyperactivity concentration disorders, or any disorder.

Child Revised Impact of Events Scale (CRIES-8) ***(7-17 years)*** is an eight-item screen for post-traumatic stress symptoms in children aged between 7 and 18 years, with established reliability and validity.[22] It has been used previously in the PICU population.[23] A cut-off score of 17 or greater has been found to classify correctly over 80% of children with a diagnosis of post-traumatic stress disorder.[24]

Children's Hope Scale (CHS) ***(8-17 years)*** [25] is a brief, six-item self-report measure of children's perceptions that their goals can be met. It has been validated for use in children and young people aged 8-17 years consisting of both healthy populations and children with a range of physical illnesses. Internal consistency estimates (alpha) for the samples ranged from 0.72 to 0.86. Test-retest reliability estimates (over a one-month interval) ranged from 0.71 to 0.73 [25].

Parent related measures

PedsQL™ Family Impact Module (FIM) Version 2.0 measures the impact of pediatric health conditions on family functioning [26]. It is completed by the parents and includes eight dimensions (physical functioning; emotional functioning; social functioning; cognitive functioning; communication; worry; daily activities; family relationships).

State-Trait Anxiety Inventory 6 (STAI-6) [27] is a self-reported questionnaire that assesses symptoms of anxiety. It is a short version of the Spielberger State Anxiety Scale (SSA), with a cut-off point at 1 SD above the mean to indicate clinically relevant symptoms[27].

Patient Health Questionnaire-4 (PHQ-4) [28] is a 4 item inventory rated on a 4 point Likert-type scale. Its purpose is to allow for very brief and accurate measurement of depression and anxiety. PHQ–4 scores are strongly associated with decrements in multiple domains of

For peer review only

functional impairment; the anxiety and depression subscales make unique overall contributions to the PHQ–4, both in terms of factorial and criterion validity; and perhaps most importantly: the results indicate that anxiety has a substantial independent effect on functioning, and even more so when comorbid with depression.

The Post Traumatic Stress Disorder (PTSD) Checklist for DSM-5 (PCL-5) is a 20-item self-report measure that assesses the presence and severity of PTSD symptoms [29]. Items on the PCL-5 correspond with DSM-5 criteria for PTSD. The PCL-5 can be used to quantify and monitor symptoms over time, to screen individuals for PTSD, and to assist in making a provisional or temporary diagnosis of PTSD. The PCL-5 is a psychometrically sound measure of DSM-5 PTSD. It is valid and reliable, useful in quantifying PTSD symptom severity, and sensitive to change over time [30 31].

Sibling related measures

The **PedsQL™ 4.0, SDQ** and **Children's Hope Scale** (as outlined in the Child related measures section above) will also be administered to sibling participants.

Multidimensional Assessment of Caring Activities (MACA-YC18) ***(8-17 years)*** [32] is an 18-item self-report measure that can be used to provide an index of the total amount of caring activity undertaken by the young person, as well as six subscale scores for domestic tasks, household management, personal care, emotional care, sibling care, and financial/practical care. The MACA-YC18 was designed as a short, easy to use, psychometric measure able to provide an index of the extent of caring activities that the young person is currently engaged in. Higher scores indicate greater levels of caring activity.[32 33]

Positive and Negative Outcomes of Caring (PANOC-YC20) ***(8-17 years)*** [32] a self-report measure that can be used to provide an index of positive and negative outcomes of caring. The PANOC-YC20 consists of two 10-item subscales: (1) positive responses, and (2) negative responses, which collectively assess the subjective cognitive and emotional impact of caring in young people with higher scores indicating greater positive and negative responses, respectively.

For peer review only

For peer review only

1. Pollack MM, Holubkov R, Funai T, et al. Pediatric intensive care outcomes: development of new morbidities during pediatric critical care. *Pediatr Crit Care Med* 2014;15(9):821-7. doi: 10.1097/PCC.0000000000000250 [published Online First: 2014/09/17]
2. Seid M, Limbers CA, Driscoll KA, et al. Reliability, validity, and responsiveness of the pediatric quality of life inventory (PedsQL) generic core scales and asthma symptoms scale in vulnerable children with asthma. *J Asthma* 2010;47(2):170-7. doi: 10.3109/02770900903533966 [published Online First: 2010/02/23]
3. Seid M, Varni JW, Gidwani P, et al. Problem-solving skills training for vulnerable families of children with persistent asthma: report of a randomized trial on health-related quality of life outcomes. *J Pediatr Psychol* 2010;35(10):1133-43. doi: 10.1093/jpepsy/jsp133 [published Online First: 2010/01/12]
4. Varni JW, Seid M, Knight TS, et al. The PedsQL 4.0 Generic Core Scales: sensitivity, responsiveness, and impact on clinical decision-making. *J Behav Med* 2002;25(2):175-93. doi: 10.1023/a:1014836921812 [published Online First: 2002/04/30]
5. Varni JW, Seid M, Smith Knight T, et al. The PedsQL in pediatric rheumatology: reliability, validity, and responsiveness of the Pediatric Quality of Life Inventory Generic Core Scales and Rheumatology Module. *Arthritis Rheum* 2002;46(3):714-25. doi: 10.1002/art.10095 [published Online First: 2002/03/29]
6. Mistry RD, Stevens MW, Gorelick MH. Health-related quality of life for pediatric emergency department febrile illnesses: an evaluation of the Pediatric Quality of Life Inventory 4.0 Generic Core Scales. *Health Qual Life Outcomes* 2009;7:5. doi: 10.1186/1477-7525-7-5 [published Online First: 2009/01/31]
7. Brandow AM, Brousseau DC, Pajewski NM, et al. Vaso-occlusive painful events in sickle cell disease: impact on child well-being. *Pediatr Blood Cancer* 2010;54(1):92-7. doi: 10.1002/pbc.22222 [published Online First: 2009/08/05]
8. Aspesberro F, Fesinmeyer MD, Zhou C, et al. Construct Validity and Responsiveness of the Pediatric Quality of Life Inventory 4.0 Generic Core Scales and Infant Scales in the PICU. *Pediatr Crit Care Med* 2016;17(6):e272-9. doi: 10.1097/PCC.0000000000000727 [published Online First: 2016/06/05]
9. Ong C, Lee JH, Leow MK, et al. Functional Outcomes and Physical Impairments in Pediatric Critical Care Survivors: A Scoping Review. *Pediatr Crit Care Med* 2016;17(5):e247-59. doi: 10.1097/PCC.0000000000000706 [published Online First: 2016/04/01]
10. Janssens L, Gorter JW, Ketelaar M, et al. Health-related quality-of-life measures for long-term follow-up in children after major trauma. *Quality of Life Research* 2008;17(5):701-13. doi: 10.1007/s11136-008-9339-0 [published Online First: 2008/04/26]
11. Eiser C, Morse R. The measurement of quality of life in children: past and future perspectives. *Journal of Developmental & Behavioral Pediatrics* 2001;22(4):248-56. [published Online First: 2001/09/04]
12. Varni JW, Burwinkle TM, Katz ER, et al. The PedsQL in pediatric cancer: reliability and validity of the Pediatric Quality of Life Inventory Generic Core Scales, Multidimensional Fatigue Scale, and Cancer Module. *Cancer* 2002;94(7):2090-106. doi: 10.1002/cncr.10428 [published Online First: 2002/04/05]
13. Aspesberro F, Mangione-Smith R, Zimmerman JJ. Health-related quality of life following pediatric critical illness. *Intensive Care Med* 2015;41(7):1235-46. doi: 10.1007/s00134-015-3780-7 [published Online First: 2015/04/09]
14. Pollack MM, Holubkov R, Glass P, et al. Functional Status Scale: new pediatric outcome measure. *Pediatrics* 2009;124(1):e18-28. doi: 10.1542/peds.2008-1987 [published Online First: 2009/07/01]

For peer review only

15. Pollack MM, Holubkov R, Funai T, et al. Simultaneous Prediction of New Morbidity, Mortality, and Survival Without New Morbidity From Pediatric Intensive Care: A New Paradigm for Outcomes Assessment. *Crit Care Med* 2015;43(8):1699-709. doi: 10.1097/CCM.0000000000001081 [published Online First: 2015/05/20]
16. Fiser DH, Long N, Roberson PK, et al. Relationship of pediatric overall performance category and pediatric cerebral performance category scores at pediatric intensive care unit discharge with outcome measures collected at hospital discharge and 1-and 6-month follow-up assessments. *Critical care medicine* 2000;28(7):2616-20.
17. Fiser DH, Tilford JM, Roberson PK. Relationship of illness severity and length of stay to functional outcomes in the pediatric intensive care unit: a multi-institutional study. *Critical care medicine* 2000;28(4):1173-79. [published Online First: 2000/05/16]
18. Fiser DH. Assessing the outcome of pediatric intensive care. *The Journal of pediatrics* 1992;121(1):68-74. [published Online First: 1992/07/01]
19. Fuchs S, Lewis RJ. Tools for the measurement of outcome after minor head injury in children: summary from the Ambulatory Pediatric Association/EMSC Outcomes Research Conference. *Academic emergency medicine* 2003;10(4):368-75. [published Online First: 2003/04/03]
20. Goodman R. Psychometric properties of the strengths and difficulties questionnaire. *Journal of the American Academy of Child & Adolescent Psychiatry* 2001;40(11):1337-45. doi: 10.1097/00004583-200111000-00015 [published Online First: 2001/11/09]
21. Goodman R. The Strengths and Difficulties Questionnaire: A Research Note. *Journal of Child Psychology and Psychiatry* 1997;38(5):581-86. doi: 10.1111/j.1469-7610.1997.tb01545.x [published Online First: 1997/07/01]
22. Yule W. Anxiety, depression and post-traumatic stress in childhood. . In: Sclare I, ed. Child psychology portfolio. Windsor, UK: NFER-Nelson 1997:35-38.
23. Colville G, Pierce C. Patterns of post-traumatic stress symptoms in families after paediatric intensive care. *Intensive Care Med* 2012;38(9):1523-31. doi: 10.1007/s00134-012-2612-2 [published Online First: 2012/06/19]
24. Perrin S, Meiser-Stedman R, Smith P. The Children's Revised Impact of Event Scale (CRIES): Validity as a screening instrument for PTSD. *Behavioural and Cognitive Psychotherapy* 2005;33(4):487-98.
25. Snyder CR, Hoza B, Pelham WE, et al. The Development and Validation of the Children's Hope Scale. *Journal of Pediatric Psychology* 1997;22(3):399-421. doi: 10.1093/jpepsy/22.3.399
26. Varni JW, Sherman SA, Burwinkle TM, et al. The PedsQL Family Impact Module: preliminary reliability and validity. *Health Qual Life Outcomes* 2004;2:55. doi: 10.1186/1477-7525-2-55 [published Online First: 2004/09/29]
27. Marteau TM, Bekker H. The development of a six-item short-form of the state scale of the Spielberger State—Trait Anxiety Inventory (STAI). *British Journal of Clinical Psychology* 1992;31(3):301-06. doi: 10.1111/j.2044-8260.1992.tb00997.x
28. Kroenke K, Spitzer RL, Williams JB, et al. An ultra-brief screening scale for anxiety and depression: the PHQ–4. *Psychosomatics* 2009;50(6):613-21.
29. Weathers FW, Litz BT, Keane TM, et al. The PTSD checklist for DSM-5 (PCL-5). US: Department of Veteran Affairs: National Center for PTSD 2013.
30. Blevins CA, Weathers FW, Davis MT, et al. The posttraumatic stress disorder checklist for DSM-5 (PCL-5): Development and initial psychometric evaluation. *Journal of traumatic stress* 2015;28(6):489-98. doi: 10.1002/jts.22059 [published Online First: 2015/11/26]

For peer review only

31. Bovin MJ, Marx BP, Weathers FW, et al. Psychometric properties of the PTSD Checklist for Diagnostic and Statistical Manual of Mental Disorders–Fifth Edition (PCL-5) in veterans. *Psychological Assessment* 2016;28(11):1379.
32. Joseph S, Becker F, Becker S. Manual for measures of caring activities and outcomes for children and young people. Nottingham: The University of Nottingham 2009.
33. Joseph S, Becker S, Becker F, et al. Assessment of caring and its effects in young people: development of the Multidimensional Assessment of Caring Activities Checklist (MACA-YC18) and the Positive and Negative Outcomes of Caring Questionnaire (PANOC-YC20) for young carers. *Child: care, health and development* 2009;35(4):510-20. doi: 10.1111/j.1365-2214.2009.00959.x [published Online First: 2009/03/27]

For peer review only

BMJ Open

A study protocol for a multi-centre longitudinal mixed methods study to explore the Outcomes of ChildrEn and fAmilies in the first year after paediatric Intensive Care: The OCEANIC Study

Journal:	*BMJ Open*
Manuscript ID	bmjopen-2020-038974.R1
Article Type:	Protocol
Date Submitted by the Author:	16-Apr-2020
Complete List of Authors:	Manning, Joseph; University of Nottingham, Children and Young People Health Research, School of Health Sciences; Nottingham University Hospitals NHS Trust, Nottingham Children's Hospital<br>Latour, Jos; University of Plymouth School of Nursing and Midwifery; Hunan Children's Hospital, Nursing Department<br>Curley, Martha ; University of Pennsylvania, Department of Family and Community Health, School of Nursing ; University of Pennsylvania Perelman School of Medicine, Anesthesia and Critical Care Medicine<br>Draper, Elizabeth; University of Leicester, Department of Health Sciences<br>Jilani, Tahseen; University of Nottingham, Advanced Data Analysis Centre (ADAC); University of Nottingham, Health Data Research UK<br>Quinlan, Philip; University of Nottingham, Advanced Data Analysis Centre (ADAC); University of Nottingham, Health Data Research UK<br>Watson, R; University of Washington, Division of Pediatric Critical Care Medicine, Department of Pediatrics; Seattle Children's Research Institute, Centre for Child Health, Behaviour, and Development<br>Rennick, Janet; McGill University Faculty of Medicine, Ingram School of Nursing; Research Institute of the McGill University Health Centre, Centre for Outcomes Research & Evaluation<br>Colville, Gillian; St Georges University Hospitals NHS Foundation Trust, Paediatric Psychology Service; University of London St George's, Population Health Research Institute<br>Pinto, Neethi; University of Chicago, Section of Pediatric Critical Care, Department of Pediatrics<br>Latif, Asam; University of Nottingham, School of Health Sciences<br>Popejoy, Emma; University of Nottingham, Children and Young People Health Research, School of Health Sciences; Nottingham University Hospitals NHS Trust, Nottingham Children's Hospital<br>Coad, Jane; University of Nottingham, Children and Young People Health Research, School of Health Sciences
**Primary Subject Heading**:	Intensive care
Secondary Subject Heading:	Paediatrics
Keywords:	Paediatric intensive & critical care < ANAESTHETICS, STATISTICS & RESEARCH METHODS, QUALITATIVE RESEARCH

SCHOLARONE™
Manuscripts

I, the Submitting Author has the right to grant and does grant on behalf of all authors of the Work (as defined in the below author licence), an exclusive licence and/or a non-exclusive licence for contributions from authors who are: i) UK Crown employees; ii) where BMJ has agreed a CC-BY licence shall apply, and/or iii) in accordance with the terms applicable for US Federal Government officers or employees acting as part of their official duties; on a worldwide, perpetual, irrevocable, royalty-free basis to BMJ Publishing Group Ltd ("BMJ") its licensees and where the relevant Journal is co-owned by BMJ to the co-owners of the Journal, to publish the Work in this journal and any other BMJ products and to exploit all rights, as set out in our licence.

The Submitting Author accepts and understands that any supply made under these terms is made by BMJ to the Submitting Author unless you are acting as an employee on behalf of your employer or a postgraduate student of an affiliated institution which is paying any applicable article publishing charge ("APC") for Open Access articles. Where the Submitting Author wishes to make the Work available on an Open Access basis (and intends to pay the relevant APC), the terms of reuse of such Open Access shall be governed by a Creative Commons licence – details of these licences and which Creative Commons licence will apply to this Work are set out in our licence referred to above.

Other than as permitted in any relevant BMJ Author's Self Archiving Policies, I confirm this Work has not been accepted for publication elsewhere, is not being considered for publication elsewhere and does not duplicate material already published. I confirm all authors consent to publication of this Work and authorise the granting of this licence.

For peer review only

A study protocol for a multi-centre longitudinal mixed methods study to explore the Outcomes of ChildrEn and fAmilies in the first year after paediatric Intensive Care: The OCEANIC Study

Authors and Affiliations

Joseph C. Manning, RN, PhD [1,2,10], Jos M. Latour, RN, PhD [3,4], Martha A.Q. Curley, RN, PhD[5,6,7], Elizabeth S. Draper, PhD[8], Tahseen Jilani, PhD[9,10], Philip R. Quinlan, PhD[9,10], R. Scott Watson, MD, MPH[11,12], Janet E. Rennick, RN, PhD[13,14,15], Gillian Colville, MPhil, CPsychol[16,17], Neethi Pinto, MD, MS[18], Asam Latif, MGPhC, PhD[2], Emma Popejoy, RN, PhD[1,2], Jane Coad, RN, PhD[2], *for the OCEANIC Study Investigators*

[1]Nottingham Children's Hospital, Nottingham University Hospitals NHS Trust, Nottingham, UK.

[2]Children and Young People Health Research, School of Health Sciences, Faculty of Medicine and Health Sciences, University of Nottingham, Nottingham, UK.

[3]School of Nursing and Midwifery, Faculty of Health, University of Plymouth, UK.

[4]Nursing Department, Hunan Children's Hospital, Changsha, People's Republic of China.

[5]Department of Family and Community Health, School of Nursing, University of Pennsylvania, Philadelphia, PA, USA

[6]Anesthesia and Critical Care Medicine, Perelman School of Medicine, University of Pennsylvania, Philadelphia, PA, USA

[7]The Research Institute, Children's Hospital of Philadelphia, Philadelphia, PA, USA

[8]Department of Health Sciences, College of Life Sciences, University of Leicester, UK.

[9]Advanced Data Analysis Centre, The University of Nottingham, Nottingham, UK.

[10]Health Data Research UK, The University of Nottingham, Nottingham, UK.

[11]Division of Pediatric Critical Care Medicine, Department of Pediatrics, University of Washington, Seattle, WA, USA.

For peer review only - http://bmjopen.bmj.com/site/about/guidelines.xhtml

[12]Centre for Child Health, Behaviour, and Development, Seattle Children's Research Institute, Seattle, WA, USA

[13]Ingram School of Nursing, Faculty of Medicine, McGill University, Montreal, Quebec, Canada

[14]The Montreal Children's Hospital, McGill University Health Centre, Montreal, Quebec, Canada

[15]Centre for Outcomes Research & Evaluation, Research Institute of the McGill University Health Centre, Montreal, Quebec, Canada

[16]Paediatric Psychology Service, St George's University Hospitals NHS Foundation Trust, London, UK.

[17]Population Health Research Institute, St George's University of London, London, UK.

[18]Section of Pediatric Critical Care, Department of Pediatrics, University of Chicago, Chicago, IL, USA.

Corresponding Author:

Dr Joseph C. Manning, Clinical Associate Professor / NIHR HEE ICA Clinical Lecturer, SC3084, C-floor, East Block, QMC Campus, Derby Road, Nottingham. NG7 2UH.
joseph.manning@nottingham.ac.uk.

Clinical Trials Registration Number: ISRCTN 28072812

Keywords: Pediatric Intensive Care Unit, Post Intensive Care Syndrome-pediatrics, Child, Parent, Sibling, Outcome

Running Header: The OCEANIC Study Protocol

Word Count: 4353 words

For peer review only - http://bmjopen.bmj.com/site/about/guidelines.xhtml

ABSTRACT

Introduction: Annually in the UK 20,000 children become very ill or injured and need specialist care within a Paediatric Intensive Care Unit (PICU). Most children survive. However, some children and their families may experience problems after they have left the PICU including physical, functional, and/or emotional problems. It is unknown which children and families experience such problems, when these occur or what causes them. The aim of this mixed-method longitudinal cohort study is to understand the physical, functional, emotional and social impact of children surviving PICU (aged: 1 month-17 years), their parents and siblings, during the first-year after a PICU admission.

Methods and analysis: A quantitative study involving 300 child survivors of PICU; 300 parents; and 150-300 siblings will collect data (using self-completion questionnaires) at baseline, PICU discharge, 1, 3, 6 and 12 months post-PICU discharge. Questionnaires will comprise of validated and reliable instruments. Demographic data, PICU admission and treatment data, health related quality of life, functional status, strengths and difficulties behaviour and post-traumatic stress symptoms will be collected from the child. Parent and sibling data will be collected on the impact of paediatric health conditions on the family's functioning capabilities, levels of anxiety and social impact of the child's PICU admission. Data will be analysed using descriptive and inferential statistics. Concurrently, an embedded qualitative study involving semi-structured interviews with 24 enrolled families at 3 months and 9 months post-PICU discharge will be undertaken. Framework analysis will be used to analyse the qualitative data.

Ethics and dissemination: The study has received ethical approval from the National Health Services Research Ethics Committee [Ref: 19/WM/0290] and full governance clearance. This will be the first UK study to comprehensively investigate physical, functional, emotional and social consequences of PICU survival in the first year post-discharge.

For peer review only

For peer review only - http://bmjopen.bmj.com/site/about/guidelines.xhtml

Article Summary

Strengths and limitations of this study

- The OCEANIC study will be the first multisite, comprehensive study conducted in the UK to investigate the physical, functional, emotional and social consequences of PICU survival in the first year post-discharge.
- Our longitudinal study design will allow us to look at changes over time in the same patient/family, providing insights into the temporal sequence of changes that may occur as a result of childhood critical illness/injury.
- The qualitative study (interviews with children, parents and siblings) will be analysed in conjunction with quantitative data allowing a fuller understanding of physical, functional, emotional and social consequences of being on PICU and any outstanding needs.
- The primary limitation of this study is loss to follow-up and missing data points that would challenge the internal validity of reported results from The OCEANIC study.

For peer review only

For peer review only - http://bmjopen.bmj.com/site/about/guidelines.xhtml

INTRODUCTION

In the United Kingdom (UK) annually, approximately 20,000 children (aged 0-18 years) experience a critical illness, requiring paediatric intensive care unit (PICU) treatment and care.[1] Despite increasing demand on paediatric critical care services, PICU survival has increased substantially over the past three decades, rendering mortality alone an insufficient metric for outcomes assessment post-PICU discharge.[2] Over 96% of children admitted to PICU survive.[1] However, the decline in mortality has been accompanied by a concomitant increase in morbidity.[3] Evidence is building which portrays a cohort of PICU survivors who are physically deconditioned, cognitively impaired, and emotionally distraught. The emotional and social health of the PICU survivor's parents and siblings may also be affected.[4, 5]

Two systematic reviews reported that approximately 25% of critically ill children exhibited negative psychological and behavioural responses within the first-year post-discharge.[6, 7] Similar themes were identified in a systematic review of qualitative studies examining the psychosocial impact of PICU hospitalization on children,[8] lending support to the importance in identifying children suffering from psychological sequelae. Given that psychological well-being is shaped by multiple factors, alterations in the child's sense of self and interpersonal relationships, as well as ongoing worries and fears about hospitalization, have the potential to affect recovery during the early post-discharge period, and during critical periods of growth and development. Health related quality of life (HRQOL) studies identify deterioration in the emotional well-being of 20-30% of children up to 1-year post-PICU discharge, [6, 7] suggesting a sustained effect.

The impact of a child's critical illness on family members may be profound as they, too, can experience psychosocial sequelae.[5, 9] Family members' responses may, in turn, influence the outcomes of child survivors following paediatric critical illness. Furthermore there is evidence that critical illness impacts a family's social functioning in relation to re-integration with peers; the child and family's social capital; and the economic impact of unemployment on families when a care-giver has to relinquish work responsibilities to care for a child.[10] However, the interplay between the child, their parent and siblings' outcomes, caregiver roles, and family needs, and how these change over time, are largely absent in the literature.

Globally [11-13] and in the UK [14, 15] researchers, clinicians, and patients and their families have recognised understanding and supporting adult survivors of intensive care is both a

For peer review only - http://bmjopen.bmj.com/site/about/guidelines.xhtml

research and clinical priority. Patient and public consultation conducted with the PICU community (including children, their families, service providers and commissioners) confirms that understanding and optimising the outcomes of children and their families is also a research priority for childhood survivors of PICU [16]

METHODS AND ANALYSIS

Study purpose and objectives

The purpose of the OCEANIC study is to explore child PICU survivors' health outcomes and family impact over one-year post-PICU discharge. In order to identify morbidities, when they occur, and whether there are factors that could be modified to improve the health and well-being of PICU survivors and their families.

OCEANIC has four specific objectives:

1. To describe the physical, cognitive, emotional, and social health outcomes and trajectory of recovery in children post-PICU discharge.
2. To determine the baseline and PICU factors associated with impaired outcomes.
3. To explore the longitudinal emotional and social health outcomes of parents and siblings.
4. To ascertain the care and support needs of children and their parents and siblings.

Theoretical Framework

Based upon a state-of-the-science review of post-discharge outcomes in paediatric critical care [17], a conceptual framework describing the constellation of potential physical, cognitive, emotional, and social health effects that may be uniquely experienced by children and families who survive paediatric critical illness has been proposed (**Error! Reference source not found.**)[18]. This framework incorporates the importance of pre-existing health status, sociodemographic data, physiologic maturation, and psychosocial development on the trajectory of health recovery over a child's lifetime. Additionally, the framework recognizes that the interdependence of the child and family is central to understanding the long-term multidimensional sequelae of paediatric critical illness. This framework provides a roadmap

For peer review only - http://bmjopen.bmj.com/site/about/guidelines.xhtml

for understanding longitudinal outcomes; the proposed study will organize data collection using this framework.

This embedded mixed-methods study involves two linked work-packages (overview presented in Figure 2). The first work-package will be a quantitative study involving 300 child survivors of critical illness; 300 parents; and 150-300 siblings. The second work-package will be a qualitative interview study of two cohorts of 12 families, at 3 and 9 months post PICU discharge. Mixing will occur through the sampling and selection of participants for the embedded qualitative study from those enrolled in the quantitative study, as well as in the framework analysis.

Quantitative study

Data regarding the PICU admission of each child participant will be downloaded from the Paediatric Intensive Care Audit Network (PICANet) database, a secure and confidential high quality clinical database of paediatric intensive care activity in the UK and Ireland. Data extracted will include: demographic and socioeconomic data; pre-PICU health status; and acute illness data (PICU admission and discharge diagnoses; co-morbidities; operations and invasive procedures performed; type of admission (planned/unplanned); PICU and hospital length of stay, duration of mechanical ventilation, high frequency oscillatory ventilation, extracorporeal membrane oxygenation, renal replacement therapy, and vasopressor/inotropic support; sedative medications and days of exposure). Outcome data will also be collected from each child (or proxy), their parent, and sibling (if appropriate) prospectively over the first-year post-PICU discharge.

Study measures

Currently there are no standardised or agreed set of outcome measures for research with the PICU patient population. Therefore, the outcome measures used in this study were selected for their validity, reliability, ease of use, availability in electronic versions and previous use with the population under investigation. Furthermore, the focus and selection of these measures was informed by the Post Intensive Care Syndrome in pediatrics (PICS-p) framework, contemporary literature, and consultation with patients, public, and PICU clinicians. In line with feedback from patient and public involvement (PPI) consultations, outcomes will be

For peer review only - http://bmjopen.bmj.com/site/about/guidelines.xhtml

collected at six time-points: Baseline status (pre-PICU discharge); at PICU discharge; 1, 3, 6 and 12 months post-PICU discharge. The outcomes measured and time points are outlined in Table 1.

Data collection measures, versions, and report format according to age and study participant (child PICU survivor, parent/legal guardian or sibling) are reported in Table 2. A brief overview of the measures is provided in *Supplementary File 1*.

Child related measures include:

- PedsQL™ 4.0 (Pediatric Quality of Life Inventory) Generic Core Scales *(2-17 years)* and Infant Scales *(1-23 months)* – Acute Version [3, 19-28]
- PedsQL™ Multi-dimensional Fatigue Scale *(2-17 years)* – Acute Version [29]
- PedsQL™ Pediatric Pain Questionnaire *(5-17 years)*
- Functional Status Scale (FSS) *(1 month-17 years)* [30-32]
- Pediatric Cerebral Performance Category (PCPC) and the Pediatric Overall Performance Category (POPC) *(1 month – 17 years)* [33-36]
- Strengths and Difficulties Questionnaire (SDQ) *(2-17 years)* [37, 38]
- Child Revised Impact of Events Scale (CRIES-8) *(7-17 years)* [39-41]
- Children's Hope Scale (CHS) *(8-17 years)* [42]

Parent related measures

- PedsQL™ Family Impact Module (FIM) Version 2.0 [43]
- State-Trait Anxiety Inventory 6 (STAI-6) [44]
- Patient Health Questionnaire-4 (PHQ-4) [45]
- The Post Traumatic Stress Disorder (PTSD) Checklist for DSM-5 (PCL-5)[46-48].

Sibling related measures

- PedsQL™ 4.0 (Pediatric Quality of Life Inventory) Generic Core Scales *(2-17 years)* [3, 19-28]
- Children's Hope Scale (CHS) *(8-17 years)* [42]
- Multidimensional Assessment of Caring Activities (MACA-YC18) *(8-17 years)* [49, 50]
- Positive and Negative Outcomes of Caring (PANOC-YC20) *(8-17 years)* [50]

For peer review only

For peer review only - http://bmjopen.bmj.com/site/about/guidelines.xhtml

Qualitative study

The second work-package will be a qualitative study involving semi-structured interviews with 24 families, split between 3 and 9 months post-PICU discharge. As advocated in the child health literature, a pragmatic and participant-centred approach (based on choice, participation, and flexibility) to collecting qualitative data will be employed. Interviews will be conducted with children, parents/legal guardians, and siblings either collectively or separately. Interviews will take place at the participants' preferred time and method (e.g. face-to-face, telephone). The use of multiple sources of data will provide contextualised, converging and emerging lines of inquiry.

Sample and recruitment

Setting

Participants will be recruited from at least five PICUs across England chosen to include variation in unit size, case mix, geographical location, and patient demographic.

Eligibility criteria

Participants for this study include: (1) PICU child survivors, (2) parents/legal guardians and (3) siblings:

1. PICU child survivor: (a) Aged 1 month (and ≥44 weeks corrected gestational age) to 17 years at the point of PICU admission; (b) will be discharged from the PICU in next 48 hours; (c) PICU total length of stay (LOS) ≥ 72 hours at point of discharge in which the patient received PICU therapies for organ dysfunction; (d) At least one parent/legal guardian (≥18 years of age or considered emancipated) living with the potential subject.

2. Parent: (a) parent or legal guardian; (b) cohabits with the child.

3. Siblings: (a) aged ≥ 8 years (at baseline); (b) is a sibling of the children PICU survivor; (c) cohabits with the child PICU survivor for at least 50% of the time; (d) can independently self-report.

Sample

For peer review only - http://bmjopen.bmj.com/site/about/guidelines.xhtml

Sample size

Quantitative study: We anticipate enrolling 300 children (and their families) from five PICUs in equal proportions (60 per centre) over a 6-month period. Based on previous PICU studies [51, 52], we conservatively estimate a 20% attrition rate over one year. Thus, we anticipate having one-year outcomes for 240 patients at the end of the study. With 240 participants, we will have high power to detect small/moderate correlations between early PedsQL™ measurements (to assess the trajectory of recovery) and other baseline and PICU factors with one-year PedsQL™ summary scores. Using a two-sided 0.05 level test, we have 80% power to detect correlations of 0.18 or larger in magnitude. With 240 participants, we will also have high power to detect moderate differences when comparing two groups using a t-test (e.g., comparison of PedsQL™ summary scores by gender or diagnosis category). In addition, many of the analyses will involve multiple linear regression modelling to adjust for baseline factors or confounding variables. With 240 participants, there is high power for the assessment of modest covariate effects with linear regression. Thus, we anticipate having high power for assessing correlations or linear regression effects as well as for comparing groups with our expected one-year sample size.

Qualitative study: A stratified sample of up to 24 families (which may include the child, parent and sibling, with a maximum of 72 participants in total) will be enrolled into the qualitative interviews. This sample size will capture diverse perspectives around support needs and is expected to achieve data saturation in the qualitative analysis [53].

Sampling technique

Quantitative study sampling technique: A consecutive sampling strategy will be employed [54]. Each site will screen daily over a 12-month period and invite all eligible children to participate in the study. Data from screening logs, including refusal to participate and admission numbers at each site, will be collected and used to contextualise the reporting of the analysis. In order to recruit a sample that is representative of the PICU populous, a sampling frame based on age and diagnosis reported from PICANet data [1] will be used. This frame will be used to guide the recruitment of participants recruited into the study and is outlined in Table 3.

For peer review only - http://bmjopen.bmj.com/site/about/guidelines.xhtml

Qualitative study sampling technique: Two cohorts of 12 families (including the child, parent and a sibling) will be selected using a stratified sampling approach based on the child's PedsQL™ score at 1 month post-PICU discharge and 6 months post-PICU discharge. Stratification using previously reported norms for PedsQL™ as well as variation in relation to geographical locality, PICU presenting condition, age and ethnicity will be sought.

Study procedures

Quantitative study

Over a six-month period, each site will screen daily the children admitted to PICU and invite all eligible children to participate in the study. Site investigators (or their designated nominee) who are part of the PICU clinical care team will determine eligibility.

In line with feedback from PPI work in the development of this study, each participant (aged ≥ 5 years) will be provided with a single £15 gift voucher as a token of appreciation for participating in the study. Vouchers will be provided to all participants on the completion of the study data collection period (T6- 12 month's post-PICU discharge).

Qualitative study

For the qualitative study, participants will be identified from PedsQL™ scores of the child participant at 1 month post-PICU discharge and 6 months post-PICU discharge. The identification and recruitment process is summarised in Figure 3 and will follow a systematic process:

1. Child participant PedsQL™ scores will be collected and submitted by sites onto REDCap Cloud.

2. The Chief Investigator will review the scores and stratify the sample based whether the PedsQL™ score is within 1, 2 or >2 standard deviations from the published norms, selecting at least 4 children for each group at 1 month post-PICU discharge and 6 months post-PICU discharge. To maximise diversity in families (child, parent and sibling) interviewed, where possible participants will be selected based on geographical locality, PICU presenting condition, age and ethnicity.

For peer review only - http://bmjopen.bmj.com/site/about/guidelines.xhtml

3. The study ID of potential participants will be sent to sites, who will then contact the family directly, requesting consent to receive contact from the Chief Investigator/study researcher.

4. The Chief Investigator/study researcher will contact families that have agreed to being contacted, to consent for qualitative interviews and to arrange suitable date, time and location.

Analyses

Quantitative study data analysis

Descriptive statistics will be presented for demographic information, and past and current medical history. All child, parent, and sibling-related measures will be calculated, including means, standard deviations, medians, and interquartile ranges for continuous variables and frequency counts and percentages for categorical variables. Data will be examined for normality, outliers, and systematic missing data. Transformations will be undertaken as needed.

Analyses related to specific objectives include the following:

Objective 1: To describe the physical, cognitive, emotional, and social health outcomes and trajectory of recovery in children post-PICU discharge. The primary aim is to explore child PICU survivors' health outcomes and trajectory of recovery over the first year post-PICU discharge. PICU survivors' health outcomes will be compared with published population means from the general and chronically ill populations using t-tests or Mann-Whitney test as appropriate. For the longitudinal data, correlations will be assessed between time points using Spearman correlations and a linear mixed regression model with random subject effects will be used to analyse trajectories over time. In case of lack of normality, the non-parametric longitudinal approach (nparLD) will be implemented.

Objective 2: To determine the baseline and PICU factors associated with impaired outcomes. To identify factors associated with impaired health outcomes among PICU survivors, correlation analyses followed by Principle Component Analysis (PCA) will be applied to identify covariates for the regression modelling. For categorised recovery over one-year post-PICU discharge, mixed effect logistic regression will be applied. Variables will be entered using backward stepwise approach to control for collinearity. Model performance will be

For peer review only - http://bmjopen.bmj.com/site/about/guidelines.xhtml

assessed using sensitivity, specificity, positive predicted value, negative predicted value and AUCROC values. Bootstrapping through K-fold approach will be applied to ensure better modelling.

Objective 3: To explore the longitudinal emotional and social health outcomes of parents and siblings. Parent and sibling emotional- and social health outcomes will be compared to published means using t-tests or Mann-Whitney test as appropriate. PICU survivor and sibling PedsQL™ summary scores and SDQ scores will also be compared using paired t-tests or Wilcoxon Signed Rank test.

Graphical analyses will be performed to display the trajectories of health outcomes over time in our populations of critically ill children. Multiple linear and logistic regression methods will be used to explore the effects of primary diagnosis (e.g., respiratory, cardiovascular), PICU length of stay category, and site, to predict outcomes. We will explore whether adjustment for sex, race/ethnicity, or site affects study inferences through the use of mixed effects and generalized estimating equations models. Finally, we will also explore the use of classification and regression trees with recursive partitioning, principal component analysis, factor analysis, and machine learning methods to help describe subgroups of patients with similar trajectories of outcome.

Qualitative study data analysis

Audio recorded interview data will be transcribed verbatim with all participant identifiable information removed. Transcription will be conducted by a service approved by Nottingham University Hospitals NHS Trust Research and Innovation Department. Confidentiality agreements will be completed. Transcripts will be imported into NVivo 12, for sorting, coding, and categorising of the data.

Qualitative data will be analysed using the adapted five-stage Framework Analysis process to achieve *Objective 4*; identification of the care and support needs of children, their parents and siblings. The five stages of Framework Analysis comprise (1) familiarisation with the data through reading full transcripts; (2) development of a theoretical framework through identification of recurring and important themes; (3) indexing and pilot charting; (4) summarising data in an analytical framework; and (5) synthesising data by mapping and interpreting [55]. Stages 1-4 will be conducted separately for respondent type (children,

For peer review only - http://bmjopen.bmj.com/site/about/guidelines.xhtml

parents, or siblings) to enable specific care and support needs to be identified and summarised. Stage 5 will then allow for data to be compared and contrasted across the respondent groups (child, parent, sibling), child's PedsQL™ score (<1, 2, or >2 standard deviations from published norms), and time-points (1-3 months or 6-9 months post-PICU discharge).

Patient & Public Involvement

Underpinned by best principles of INVOLVE, children, young people (CYP) and families have been integral to the development of this study. In 2017, the Chief Investigator and Co-Investigator (Professor Latour) organised the UK's first symposium on aftercare and rehabilitation following PICU and engaged with over 60 PICU clinicians, an ex-PICU patient, and family members. Feedback identified that: a prospective longitudinal cohort study to further understand the outcomes for CYP and their families post-PICU was needed; and the collection of data at multiple time-points over the first year would have value for CYP and their families, health professionals, and research to direct the development of future interventions.

Further PPI has been undertaken with 11 parents (seven mothers and four fathers), four siblings (aged 9-13 years) and three CYP PICU survivors (aged 11-17 years) from the East and West Midlands. Participants' varied in ethnicity and family composition, and reasons for admissions to different PICUs. The proposed study was regarded as addressing an important topic. Respondents main concerns included: the potential to trigger negative reactions from participation; the collection of information pertaining to the pre-ICU state; and the difficulty of considering their own emotional wellbeing when their focus is on their child's survival. Suggestions to address these included: certificates and vouchers to thank participants, flexibility in the method of data collection, linking up with existing support services to build reminders, and removing reference to scores within the survey/s. Making the purpose of the research more visible through study website and social media would help parents' make decisions about participating and keeping updated with the study.

As part of this study we will continue to have meaningful advice and input from PPI. An advisory group has been assembled consisting of a young person that has been critically ill, parents and carers of children that have experienced critical illness/injury, and a sibling of a critical illness survivor. It is proposed that this group will have at least six-monthly meetings to ensure they have continued and active involvement in: the management of the research;

For peer review only - http://bmjopen.bmj.com/site/about/guidelines.xhtml

developing participant information resources; contributing to the study report; and dissemination of research findings.

ETHICS AND DISSEMINATION

Ethics

This research includes recruitment of seriously ill children on a PICU and a parent and sibling. It concerns a challenging topic requiring great skill and sensitivity in data collection. The study is being carried out by an experienced research team with clinical and research expertise in children and young people who are seriously ill. Research staff will have also received one-to-one protocol training with the CI. We will ensure the first approach is from a member of the child's usual care team, and is sensitive to the situation and status of the child.

PPI is central to this project and in ensuring that it remains grounded in the experiences of patients. The associated participant facing materials will be carefully developed (with age specific information sheets and consent/assent forms) and these will be reviewed by a PPI panel. The information sheets clearly state that discussing the experience of serious illness may be distressing, and we will ask participants to consider carefully how they feel about this prospect before deciding to take part.

Consent/assent

Eligible participants will be given at least 24 hours to consider whether they wish to participate in the study. It will be made clear to the parents that they will be free to withdraw their consent for their own and/or their child's participation in the study at any time without this having any impact on their child's care. The majority of children will be sedated and on a ventilator at recruitment, therefore will be unable to provide informed consent/assent.

For those children unable to provide consent/assent at the time of enrolment into the study, consent will be obtained from their parent/legal guardian. Efforts will be made to then consent/assent the child once they are able to (e.g. have the cognitive capacity) by the site teams. In the unlikely event that a child does not wish to participate (and the parent has consented for the child), the child's wishes will be upheld and the parent/sibling will be withdrawn from the study.

For peer review only - http://bmjopen.bmj.com/site/about/guidelines.xhtml

Interviews

We recognise that the discussing/recollecting a potentially difficult experience (the PICU admission) and any ongoing health and care needs may be upsetting for survivors/parents/families [10]. Therefore, all interviews will be conducted by the Chief Investigator or the OCEANIC Research Fellow, who both have previous experience of conducting interviews with children and families on sensitive issues. Interviews will be semi-structured over 30-60 minutes with appropriate breaks if necessary. Interviews will allow participants to explore any issues in-depth, which in itself may provide opportunity for issues, feelings and emotions to be discussed. This will be facilitated by creative/child centred data collection techniques that are sensitive to exploring potentially emotive events, in a constructive manner. Families will be given the choice whether they would like to have the interview separately (child, parent and sibling) or collectively.

It will be made clear to participants at the outset that the interview can be stopped at any time should they wish. Furthermore, if the child participant, their parent/legal guardian, or sibling becomes visibly upset during the interview, the investigator will:

1. Invite the parent/legal guardian (if present) to console the child/sibling, (if not already doing so)

2. Offer to temporarily stop or terminate the visit,

3. Respect the decision made by the participant to stop/carry on the interview.

All visits with children (<16 year olds) will be conducted with the parent/legal guardian present. In cases where it is not possible for parents to be present or the child specifically requests for them not to be present a second investigator from the study team will be present. All the study investigators have an enhanced Disclosure and Barring Service check. All investigators conducting the qualitative interviews are registered with Nursing and Midwifery Council (UK, first level) and are therefore bound by codes of professional conduct and have a professional obligation to share information with other agencies (i.e. social services), if an interview participant discloses information that relates to safeguarding or child protection.

For peer review only - http://bmjopen.bmj.com/site/about/guidelines.xhtml

Ethical review

The West Midlands – The Black Country NHS Research Ethics Committee has reviewed the study protocol and provided favourable opinion [Ref: 19/WM/0290]. The Health Research Authority has also approved the protocol [IRAS: 269642]. This study has been externally peer reviewed and awarded funding through a competitive process through the NIHR [ICA-CL-2018-04-ST2-009]. The study has been registered in International Standard Randomised Controlled Trials Number (ISRCTN) 28072812.

Dissemination

Despite advances to the evidence base, a comprehensive understanding of PICU morbidity among survivors after PICU-discharge remains limited. Historically, studies have focused on specific populations and/or diseases (such as prematurity, congenital heart disease, long-stay patients) rather than on issues experienced by the post-PICU discharge population as a whole.[31, 56-62] Moreover, these studies to date have examined variable outcomes (such as functional status, health-related qualify of life, psychological well-being, adaptive behaviours) at a single time point, [31, 56-62] with few studies considering the patient's pre-PICU status. Collectively, this heterogeneity in scope severely limits understanding of morbidities experienced by children who survive critical illness, and their trajectories.[26]

Whilst there is a definite need to understand the long-term outcome trajectories of children and families, the scope and purpose of this research is to address this critical gap by being the first study to provide a comprehensive and contemporary understanding of the outcomes of children and families in the first-year post-PICU admission. This will allow for health deficits across a spectrum of domains to be identified. It will provide a better understanding of those at risk of morbidity post-PICU admission, when this manifests, its natural history and any factors that could be modified to improve outcomes. Novel and contemporary insights into the outcomes of children and their family will be established through the study findings, which has been recognised as global priority area for PICU research. Moreover, this study will enhance understanding of the health outcomes of under researched groups within the PICU populous including those very young children (<2 years), as well as those with communication/developmental impairments. Collectively, characterization of the longitudinal recovery of children, their parents and siblings post-PICU discharge will allow interventions to be identified to prevent or mitigate morbidity and therefore have the potential to optimise

For peer review only - http://bmjopen.bmj.com/site/about/guidelines.xhtml

the outcomes and lives of children and their families. Findings will impact on the delivery and configuration of current services, as well as having the potential to inform the development of new models of care that improve the quality of services for patients and families.

The dissemination strategy will be multi-faceted to ensure findings are reported in a timely and relevant manner to key stakeholders that include patients and the public, health care professionals, commissioners and policy makers, and academics. Findings will be reported within a funder report (accessible through the NIHR Academy website), professional journals, and in high quality peer-reviewed, open-access journals. In addition, members of the PPI advisory group will assist in composing a summary which will be distributed to national parent support groups and charities. Key findings will also be posted on institutional websites and social media.

For peer review only

For peer review only - http://bmjopen.bmj.com/site/about/guidelines.xhtml

DATA STATEMENT

The datasets generated during the current study are not currently publicly available due to the study being ongoing. However, data will be available from the corresponding author on reasonable request once the study is complete. Furthermore, it is proposed that all data generated or analysed during the study will be included in published article (and their supplementary information files).

ACKNOWLEDGEMENTS

The OCEANIC study was adapted from the PICS-p Study protocol developed by MAQC, RSW, GC, JR, NP and JCM.

We would like to acknowledge Dr Julie Menzies (Nurse Researcher, Birmingham Children's Hospital) in supporting the Patient and Public Involvement activities undertaken as part of the development of the OCEANIC study protocol. Furthermore we would like to acknowledge Francois Arvin-Berod (Data Manager, Research and Innovation, Nottingham University Hospitals NHS Trust) who supported the development of the OCEANIC data capture and management through the REDCap Cloud.

AUTHOR CONTRIBUTIONS

JCM is the chief investigator for the OCEANIC study. JCM, JML, MAQC, ESD, TJ, PRQ, RSW, JER, GC, NP, and JC made a substantial contribution to the conceptualization and design of the study. JCM, AL and EP drafted the first version of the manuscript. All authors critically revised the manuscript for important intellectual content, gave approval of the final version to be published, and agreed to be accountable for all aspects of the work.

CONFLICT OF INTEREST STATEMENT

The authors have no conflicts of interest to disclose.

FUNDING

JCM is recipient of a National Institute for Health Research ICA Clinical Lectureship [ICA-CL-2018-04-ST2-009] which is funding this research study.

For peer review only - http://bmjopen.bmj.com/site/about/guidelines.xhtml

REFERENCES

1. PICANet. Annual Report: Summary Report. Leeds: Universities of Leeds and Leicester; 2017.

2. Namachivayam P, Shann F, Shekerdemian L, Taylor A, van Sloten I, Delzoppo C, et al. Three decades of pediatric intensive care: Who was admitted, what happened in intensive care, and what happened afterward. Pediatr Crit Care Med. 2010;11(5):549-55.

3. Pollack MM, Holubkov R, Funai T, Clark A, Berger JT, Meert K, et al. Pediatric intensive care outcomes: development of new morbidities during pediatric critical care. Pediatr Crit Care Med. 2014;15(9):821-7.

4. Shudy M, de Almeida ML, Ly S, Landon C, Groft S, Jenkins TL, et al. Impact of pediatric critical illness and injury on families: a systematic literature review. Pediatrics. 2006;118(Supplement 3):S203-S18.

5. Abela KM, Wardell D, Rozmus C, LoBiondo-Wood G. Impact of pediatric critical illness and injury on families: An updated systematic review. Journal of Pediatric Nursing. 2020;51:21-31.

6. Rennick JE, Rashotte J. Psychological outcomes in children following pediatric intensive care unit hospitalization: a systematic review of the research. J Child Health Care. 2009;13(2):128-49.

7. Davydow DS, Richardson LP, Zatzick DF, Katon WJ. Psychiatric morbidity in pediatric critical illness survivors: a comprehensive review of the literature. Arch Pediatr Adolesc Med. 2010;164(4):377-85.

8. Manning JC, Hemingway P, Redsell SA. Long-term psychosocial impact reported by childhood critical illness survivors: a systematic review. Nursing in critical care. 2014;19(3):145-56.

9. Nelson LP, Gold JI. Posttraumatic stress disorder in children and their parents following admission to the pediatric intensive care unit: A review. Pediatric Critical Care Medicine. 2012;13(3):338-47.

10. Manning JC, Hemingway P, Redsell SA. Stories of survival: Children's narratives of psychosocial well-being following paediatric critical illness or injury. J Child Health Care. 2017;21(3):236-52.

11. Needham DM, Davidson J, Cohen H, Hopkins RO, Weinert C, Wunsch H, et al. Improving long-term outcomes after discharge from intensive care unit: report from a stakeholders' conference. Crit Care Med. 2012;40(2):502-9.

12. Spragg RG, Bernard GR, Checkley W, Curtis JR, Gajic O, Guyatt G, et al. Beyond mortality: future clinical research in acute lung injury. American journal of respiratory and critical care medicine. 2010;181(10):1121-7.

For peer review only - http://bmjopen.bmj.com/site/about/guidelines.xhtml

13. Turnbull AE, Rabiee A, Davis WE, Nasser MF, Venna VR, Lolitha R, et al. Outcome measurement in ICU survivorship research from 1970-2013: a scoping review of 425 publications. Critical care medicine. 2016;44(7):1267.

14. Reay H, Arulkumaran N, Brett SJ. Priorities for future intensive care research in the UK: results of a James Lind alliance priority setting partnership. Journal of the Intensive Care Society. 2014;15(4):288-96.

15. National Institute for Health and Care Excellence. Rehabilitation after critical illness in adults. Quality Standard [QS158]. London NICE; 2017.

16. Manning JC, Hemingway P, Redsell SA. Survived so what? Identifying priorities for research with children and families post-paediatric intensive care unit. Nursing in Critical Care. 2017;23(2):68-74.

17. Watson RS, Choong K, Colville G, Crow S, Dervan LA, Hopkins RO, et al. Life after Critical Illness in Children—Toward an Understanding of Pediatric Post-intensive Care Syndrome. The Journal of Pediatrics. 2018;198:16-24.

18. Manning JC, Pinto NP, Rennick JE, Colville G, Curley MAQ. Conceptualizing Post Intensive Care Syndrome in Children-The PICS-p Framework. Pediatr Crit Care Med. 2018;19(4):298-300.

19. Seid M, Limbers CA, Driscoll KA, Opipari-Arrigan LA, Gelhard LR, Varni JW. Reliability, validity, and responsiveness of the pediatric quality of life inventory (PedsQL) generic core scales and asthma symptoms scale in vulnerable children with asthma. J Asthma. 2010;47(2):170-7.

20. Seid M, Varni JW, Gidwani P, Gelhard LR, Slymen DJ. Problem-solving skills training for vulnerable families of children with persistent asthma: report of a randomized trial on health-related quality of life outcomes. J Pediatr Psychol. 2010;35(10):1133-43.

21. Varni JW, Seid M, Knight TS, Uzark K, Szer IS. The PedsQL 4.0 Generic Core Scales: sensitivity, responsiveness, and impact on clinical decision-making. J Behav Med. 2002;25(2):175-93.

22. Varni JW, Seid M, Smith Knight T, Burwinkle T, Brown J, Szer IS. The PedsQL in pediatric rheumatology: reliability, validity, and responsiveness of the Pediatric Quality of Life Inventory Generic Core Scales and Rheumatology Module. Arthritis Rheum. 2002;46(3):714-25.

23. Mistry RD, Stevens MW, Gorelick MH. Health-related quality of life for pediatric emergency department febrile illnesses: an evaluation of the Pediatric Quality of Life Inventory 4.0 Generic Core Scales. Health Qual Life Outcomes. 2009;7:5.

24. Brandow AM, Brousseau DC, Pajewski NM, Panepinto JA. Vaso-occlusive painful events in sickle cell disease: impact on child well-being. Pediatr Blood Cancer. 2010;54(1):92-7.

25. Aspesberro F, Fesinmeyer MD, Zhou C, Zimmerman JJ, Mangione-Smith R. Construct Validity and Responsiveness of the Pediatric Quality of Life Inventory 4.0 Generic Core Scales and Infant Scales in the PICU. Pediatr Crit Care Med. 2016;17(6):e272-9.

For peer review only - http://bmjopen.bmj.com/site/about/guidelines.xhtml

26. Ong C, Lee JH, Leow MK, Puthucheary ZA. Functional Outcomes and Physical Impairments in Pediatric Critical Care Survivors: A Scoping Review. Pediatr Crit Care Med. 2016;17(5):e247-59.

27. Janssens L, Gorter JW, Ketelaar M, Kramer WL, Holtslag HR. Health-related quality-of-life measures for long-term follow-up in children after major trauma. Quality of Life Research. 2008;17(5):701-13.

28. Eiser C, Morse R. The measurement of quality of life in children: past and future perspectives. Journal of Developmental & Behavioral Pediatrics. 2001;22(4):248-56.

29. Varni JW, Burwinkle TM, Katz ER, Meeske K, Dickinson P. The PedsQL in pediatric cancer: reliability and validity of the Pediatric Quality of Life Inventory Generic Core Scales, Multidimensional Fatigue Scale, and Cancer Module. Cancer. 2002;94(7):2090-106.

30. Aspesberro F, Mangione-Smith R, Zimmerman JJ. Health-related quality of life following pediatric critical illness. Intensive Care Med. 2015;41(7):1235-46.

31. Pollack MM, Holubkov R, Glass P, Dean JM, Meert KL, Zimmerman J, et al. Functional Status Scale: new pediatric outcome measure. Pediatrics. 2009;124(1):e18-28.

32. Pollack MM, Holubkov R, Funai T, Berger JT, Clark AE, Meert K, et al. Simultaneous Prediction of New Morbidity, Mortality, and Survival Without New Morbidity From Pediatric Intensive Care: A New Paradigm for Outcomes Assessment. Crit Care Med. 2015;43(8):1699-709.

33. Fiser DH, Long N, Roberson PK, Hefley G, Zolten K, Brodie-Fowler M. Relationship of pediatric overall performance category and pediatric cerebral performance category scores at pediatric intensive care unit discharge with outcome measures collected at hospital discharge and 1-and 6-month follow-up assessments. Critical care medicine. 2000;28(7):2616-20.

34. Fiser DH, Tilford JM, Roberson PK. Relationship of illness severity and length of stay to functional outcomes in the pediatric intensive care unit: a multi-institutional study. Critical care medicine. 2000;28(4):1173-9.

35. Fiser DH. Assessing the outcome of pediatric intensive care. The Journal of pediatrics. 1992;121(1):68-74.

36. Fuchs S, Lewis RJ. Tools for the measurement of outcome after minor head injury in children: summary from the Ambulatory Pediatric Association/EMSC Outcomes Research Conference. Academic emergency medicine. 2003;10(4):368-75.

37. Goodman R. Psychometric properties of the strengths and difficulties questionnaire. Journal of the American Academy of Child & Adolescent Psychiatry. 2001;40(11):1337-45.

38. Goodman R. The Strengths and Difficulties Questionnaire: A Research Note. Journal of Child Psychology and Psychiatry. 1997;38(5):581-6.

39. Yule W. Anxiety, depression and post-traumatic stress in childhood. . In: Sclare I, editor. Child psychology portfolio. Windsor, UK: NFER-Nelson; 1997. p. 35-8.

For peer review only

40. Colville G, Pierce C. Patterns of post-traumatic stress symptoms in families after paediatric intensive care. Intensive Care Med. 2012;38(9):1523-31.

41. Perrin S, Meiser-Stedman R, Smith P. The Children's Revised Impact of Event Scale (CRIES): Validity as a screening instrument for PTSD. Behavioural and Cognitive Psychotherapy. 2005;33(4):487-98.

42. Snyder CR, Hoza B, Pelham WE, Rapoff M, Ware L, Danovsky M, et al. The Development and Validation of the Children's Hope Scale. Journal of Pediatric Psychology. 1997;22(3):399-421.

43. Varni JW, Sherman SA, Burwinkle TM, Dickinson PE, Dixon P. The PedsQL Family Impact Module: preliminary reliability and validity. Health Qual Life Outcomes. 2004;2:55.

44. Marteau TM, Bekker H. The development of a six-item short-form of the state scale of the Spielberger State—Trait Anxiety Inventory (STAI). British Journal of Clinical Psychology. 1992;31(3):301-6.

45. Kroenke K, Spitzer RL, Williams JB, Löwe B. An ultra-brief screening scale for anxiety and depression: the PHQ–4. Psychosomatics. 2009;50(6):613-21.

46. Weathers FW, Litz BT, Keane TM, Palmieri PA, Marx BP, Schnurr PP. The PTSD checklist for DSM-5 (PCL-5). US: Department of Veteran Affairs: National Center for PTSD 2013. Available from: https://www.ptsd.va.gov/professional/assessment/adult-sr/ptsd-checklist.asp.

47. Blevins CA, Weathers FW, Davis MT, Witte TK, Domino JL. The posttraumatic stress disorder checklist for DSM-5 (PCL-5): Development and initial psychometric evaluation. Journal of traumatic stress. 2015;28(6):489-98.

48. Bovin MJ, Marx BP, Weathers FW, Gallagher MW, Rodriguez P, Schnurr PP, et al. Psychometric properties of the PTSD Checklist for Diagnostic and Statistical Manual of Mental Disorders–Fifth Edition (PCL-5) in veterans. Psychological Assessment. 2016;28(11):1379.

49. Joseph S, Becker S, Becker F, Regel S. Assessment of caring and its effects in young people: development of the Multidimensional Assessment of Caring Activities Checklist (MACA-YC18) and the Positive and Negative Outcomes of Caring Questionnaire (PANOC-YC20) for young carers. Child: care, health and development. 2009;35(4):510-20.

50. Joseph S, Becker F, Becker S. Manual for measures of caring activities and outcomes for children and young people. Nottingham: The University of Nottingham; 2009.

51. Khetani MA, Albrecht EC, Jarvis JM, Pogorzelski D, Cheng E, Choong K. Determinants of change in home participation among critically ill children. Developmental Medicine & Child Neurology. 2018;60(8):793-800.

52. Rennick J, Dougherty G, Chambers C, Stremler R, Childerhose J, Stack D, et al. Children's psychological and behavioral responses following pediatric intensive care unit hospitalization: the caring intensively study. BMC Pediatrics. 2014;14(1):276.

53. Morse JM. Determining sample size Qualitative Health Research. 2000;10:3-5.

For peer review only - http://bmjopen.bmj.com/site/about/guidelines.xhtml

54. Daniel J. Sampling Essentials: Practical Guidelines for Making Sampling Choices. Thousand Oaks, California2012. Available from: http://methods.sagepub.com/book/sampling-essentials.

55. Ward DJ, Furber C, Tierney S, Swallow V. Using Framework Analysis in nursing research: a worked example. J Adv Nurs. 2013;69(11):2423-31.

56. Ambuehl J, Karrer A, Meer A, Riedel T, Schibler A. Quality of life of survivors of paediatric intensive care. Swiss Med Wkly. 2007;137(21-22):312-6.

57. Buysse CM, Raat H, Hazelzet JA, Hop WC, Maliepaard M, Joosten KF. Surviving meningococcal septic shock: health consequences and quality of life in children and their parents up to 2 years after pediatric intensive care unit discharge. Crit Care Med. 2008;36(2):596-602.

58. Knoester H, Bronner MB, Bos AP, Grootenhuis MA. Quality of life in children three and nine months after discharge from a paediatric intensive care unit: a prospective cohort study. Health Qual Life Outcomes. 2008;6:21.

59. Colville GA, Pierce CM. Children's self-reported quality of life after intensive care treatment. Pediatr Crit Care Med. 2013;14(2):e85-92.

60. Namachivayam SP, Alexander J, Slater A, Millar J, Erickson S, Tibballs J, et al. Five-Year Survival of Children With Chronic Critical Illness in Australia and New Zealand. Crit Care Med. 2015;43(9):1978-85.

61. Polic B, Mestrovic J, Markic J, Mestrovic M, Capkun V, Utrobicic I, et al. Long-term quality of life of patients treated in paediatric intensive care unit. Eur J Pediatr. 2013;172(1):85-90.

62. Choong K, Al-Harbi S, Siu K, Wong K, Cheng J, Baird B, et al. Functional recovery following critical illness in children: the "wee-cover" pilot study. Pediatr Crit Care Med. 2015;16(4):310-8.

For peer review only

FIGURE LEGENDS

Figure 1: Post Intensive Care Syndrome in pediatrics (PICS-p) framework; Manning et al., Pediatr Crit Care Med 2018; 19:298-300.

Figure 2: Overview of linked work packages of the OCEANIC study

Figure 3: Identification and recruitment of participants for Work Package 2- Qualitative Study

For peer review only

For peer review only - http://bmjopen.bmj.com/site/about/guidelines.xhtml

Table 1: Data collection measures and time points in which data is collected for child PICU survivor, parent/legal guardian and sibling

**Section 1: Child-survivor measures**	**Version**	**Items/ Time Required**	**T_0: Baseline (retrospective)**	**T_1: PICU Discharge**	**Post-PICU discharge** T_2: 1 month	T_3: 3 months	T_4: 6 months	T_5: 12 months
**1. Pediatric Quality of Life Inventory (PedsQL)™ Infant Scales Version 4.0 – Acute (Aged: 1-23 months)**<br>**OR**<br>**2. PedsQL™ Generic Core Scales Version 4.0 - Acute (Aged: 2 years+)**	Infant 1-12 months<br>Infant 13-23 months<br>Toddlers<br>Young Child<br>Child<br>Teen	36 items / <7min<br>45 items / <10 min<br>21 items / <5 min<br>23 items / <5 min<br>23 items / <5 min<br>23 items / <5 min	X	X	X	X	X	X
**3. PedsQL™ Multi-dimensional Fatigue Scale Version 3.0 - Acute**		18 items/ 5 min	X	X	X	X	X	X
**4. PedsQL™ Pediatric Pain Questionnaire (PPQ)™**		1 item / <1 min		X	X	X	X	X
**5. Functional Status Scale (FSS)**		6 items / 5 min	X	X	X	X	X	X
**6. Pediatric Overall Performance Category (POPC) and Pediatric Cerebral Performance Category (PCPC)**		2 item / 5 min	X	X	X	X	X	X
**7. Strengths and Difficulties Questionnaire (SDQ)**		25 items / 4 min		X	X	X	X	X
**8. Child Impact of Events Scale (CRIES-8)**		8 items / 4 minutes				X	X	X
**9. Children's Hope Scale (CHS)**		6 item / 3 minutes		X	X	X	X	X
**Max. total number of measures:**<br>*(NB for WP2 (Qualitative study) a sample of child survivors will take part in one semi-structured interview lasting approximately 30-60mins at either 1-3 months or 6-9 months post-discharge)*			**4**	7	7	**8**	8	**8**

For peer review only - http://bmjopen.bmj.com/site/about/guidelines.xhtml

For peer review only

				Post-PICU discharge			
Section 2: Parent/legal guardian measures	Items/ Time Required	T_0: Baseline (retrospective)	T_1: PICU Discharge	T_2: 1 month	T_3: 3 months	T_4: 6 months	T_5: 12 months
1. PedsQL™ Family Impact Module Version 2.0	36 items / 5 min		X	X	X	X	X
2. State-Trait Anxiety Inventory (STAI: Y-6 item)	6 items / 2 min		X	X	X	X	X
3. Patient Health Questionnaire-4 (PHQ-4)	4 items / 2 min		X	X	X	X	X
4. PTSD Checklist (PCL)-5	17 items / 5 min				X	X	X
**Total number of measures:** *(NB for WP2 (Qualitative study) a sample of parents will take part in one semi-structured interview lasting approximately 30-60mins at either 3 months or 9 months post-discharge)*		**-**	**3**	**3**	**4**	**4**	**4**
**Section 3: Sibling measures**							
1. PedsQL™ Version 4.0 Generic Core Scales	23 items / 4 min		X	X	X	X	X
2. Strengths and Difficulties Questionnaire (SDQ)	25 items / 4 min		X	X	X	X	X
3. Multidimensional Assessment of Caring Activities (MACA-YC18)	18 item / 2-4 min		X	X	X	X	X
4. Positive and Negative Outcomes of Caring (PANOC-YC20)	20 item / 2-4 min		X	X	X	X	X
5. Children's Hope Scale (CHS)	6 item / 3 minutes		X	X	X	X	X
**Total number of measures:** *(NB for WP2 (Qualitative study) a sample of siblings will take part in one semi-structured interview lasting approximately 30-60mins at either 3 months or 9 months post-discharge)*		**-**	5	**5**	**5**	5	5

For peer review only - http://bmjopen.bmj.com/site/about/guidelines.xhtml

Table 2: Data collection measures, versions, and report format according to age and study participant (child PICU survivor, parent/legal guardian or sibling)

**Section 1: Child PICU Survivor**							
	**PICU Survivor Participant Age**						
**Measure / Version (Reported by)**	1-12 months	13-23 months	2-4 years	5-7 years	8-10 years	11-12 years	13-17 years
**1. Pediatric Quality of Life Inventory (PedsQL)™ Infant Scales Version 4.0 - Acute**							
• Infants 1-12 months (Parent Reported)	X						
• Infants 13-24 months (Parent Reported)		X					
**2. PedsQL™ Generic Core Scales Version 4.0 - Acute**							
• Toddlers (Parent Reported)			X				
• Young Child (Child or Parent Reported)				X			
• Child (Child or Parent Reported)					X	X	
• Teen (Child or Parent Reported)							X
**3. PedsQL™ Multi-dimensional Fatigue Scale Version 3.0 - Acute**							
• Toddlers (Parent Reported)			X				
• Young Child (Child or Parent Reported)				X			
• Child (Child or Parent Reported)					X	X	
• Teen (Child or Parent Reported)							X
**4. PedsQL™ Pediatric Pain Questionnaire (PPQ)™**							
• Young Child (Child or Parent Reported)				X			
• Child (Child or Parent Reported)					X	X	
• Teen (Child or Parent Reported)							X
**5. Functional Status Scale (FSS)** (Parent Reported)	X	X	X	X	X	X	X
**6. Pediatric Cerebral Performance Category (PCPC) and Pediatric Overall Performance Category (POPC)** (Parent Reported)	X	X	X	X	X	X	X
**7. Strengths and Difficulties Questionnaire (SDQ)**							
• 2-4 year olds (Parent Reported)			X				
• 4-17 year olds (Parent Reported)				X	X		
• 11-17 year olds (Child Reported)						X	X
**8. Child Impact of Events Scale (CRIES-8)** (Child Reported)					X	X	X
**9. Children's Hope Scale (CHS)** (Child Reported)					X	X	X

For peer review only - http://bmjopen.bmj.com/site/about/guidelines.xhtml

Section 2: Parent/Legal guardian		
**Measure / Version (Reported by)**		**Parent/Legal guardian**
**1. Pediatric Quality of Life Inventory (PedsQL)™ Family Impact Module Version 2.0- Acute**	(Parent Reported)	X
**2. State-Trait Anxiety Inventory (STAI: Y-6 item)**	(Parent Reported)	X
**3. Patient Health Questionnaire-4 (PHQ-4)**	(Parent Reported)	X
**4. PTSD Checklist (PCL)-5**	(Parent Reported)	X

Section 3: Sibling				
**Measure / Version (Reported by)**		**Sibling Participant Age**		
		8-10 years	11-12 years	13-17 years
**1. Pediatric Quality of Life Inventory (PedsQL)™ Generic Core Scales Version 4.0- Acute**				
• Child	(Child Reported)	X	X	
• Teen	(Child Reported)			X
**2. Strengths and Difficulties Questionnaire (SDQ)**				
• 4-17 year olds	(Parent Reported)	X		
• 11-17 year olds	(Child Reported)		X	X
3. **Multidimensional Assessment of Caring Activities (MACA-YC18)**	(Child Reported)	X	X	X
**4. Positive and Negative Outcomes of Caring (PANOC-YC20)**	(Child Reported)	X	X	X
5. **Children's Hope Scale (CHS)**	(Child Reported)	X	X	X

For peer review only

For peer review only - http://bmjopen.bmj.com/site/about/guidelines.xhtml

Table 3: Proposed sampling frame for PICU survivor participant recruitment

	**Diagnosis**				
**Age (years)**	**Cardiovascular (28.1%)**	**Neurological (10.7%)**	**Respiratory (29.2%)**	**Other* (32%)**	**Total**
**0 (55%)**	47	19	48	53	167
**1-5 (25.2%)**	21	8	23	25	77
**6-10 (9.7%)**	8	3	8	9	28
**≥11 (10.3%)**	8	3	8	10	28
**Total**	84	33	87	63	300

*including: Blood/lymphatic; Body wall and cavities; Endocrine/metabolic; Trauma; Oncology; Musculo-skeletal; Multisystem; Infection; Gastrointestinal

For peer review only - http://bmjopen.bmj.com/site/about/guidelines.xhtml

Figure 1: Post Intensive Care Syndrome in pediatrics (PICS-p) framework; Manning et al., Pediatr Crit Care Med 2018; 19:298-300.

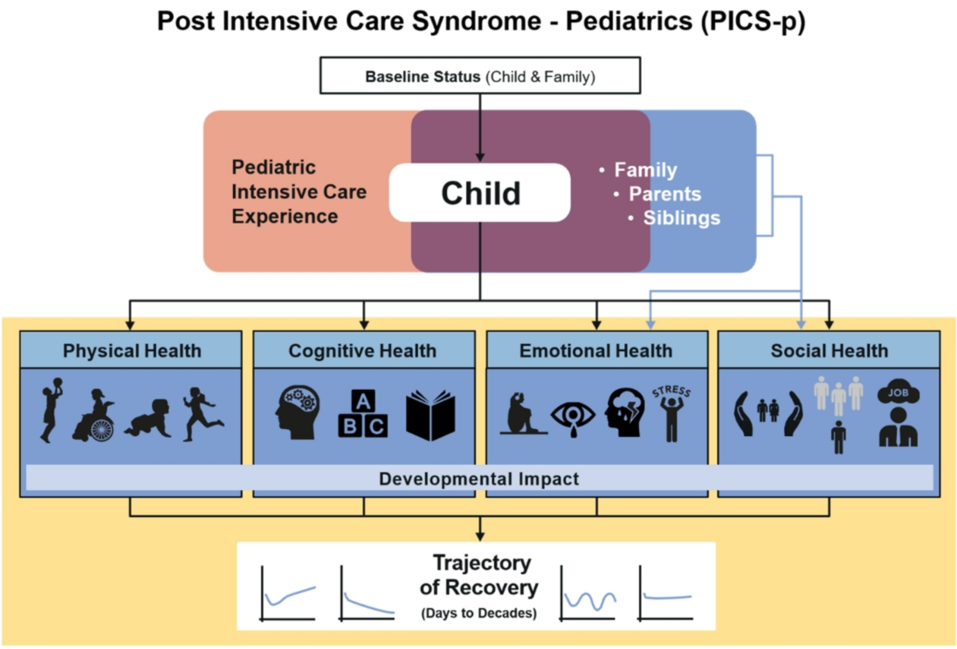

Post Intensive Care Syndrome in pediatrics (PICS-p) framework; Manning et al., Pediatr Crit Care Med 2018; 19:298-300.

166x141mm (300 x 300 DPI)

Figure 2: Overview of linked work packages of the OCEANIC study

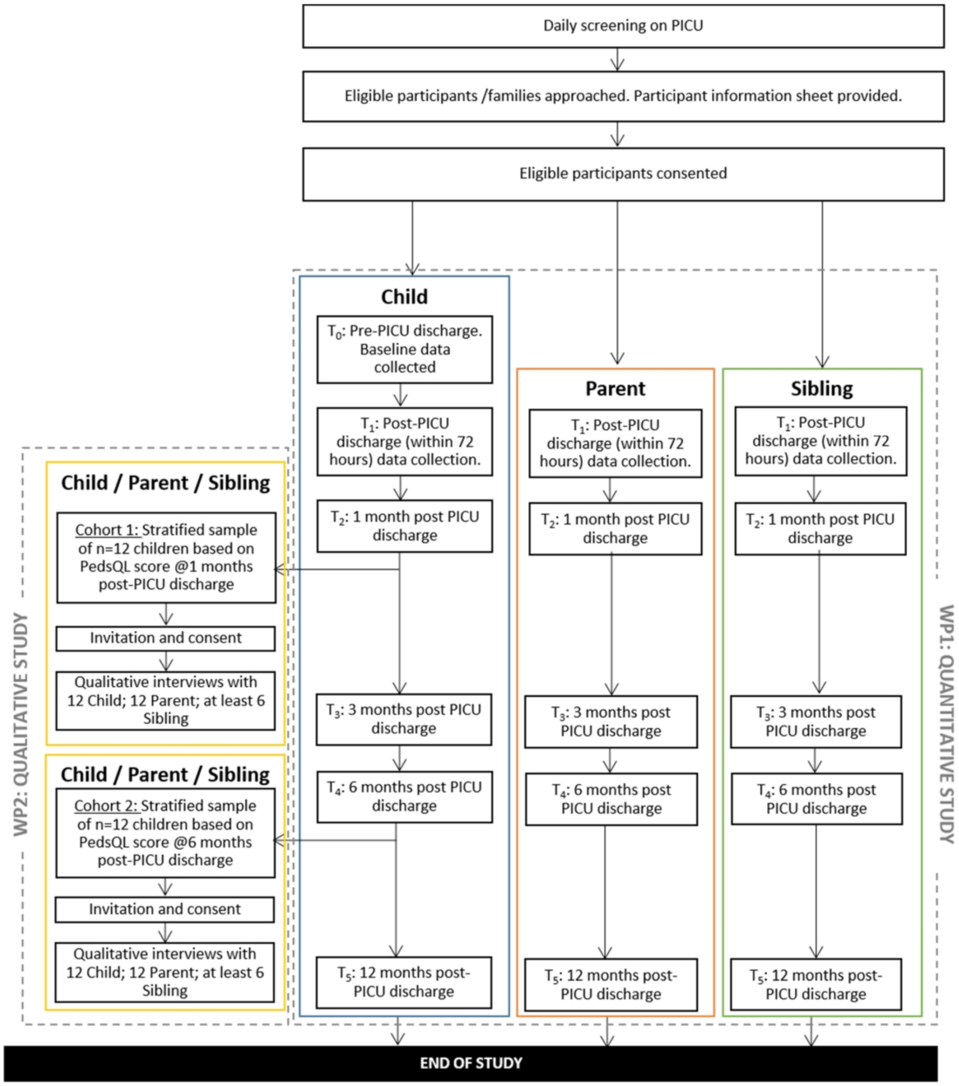

Overview of linked work packages of the OCEANIC study

175x204mm (300 x 300 DPI)

Figure 3: Identification and recruitment of participants for Work Package 2- Qualitative Study

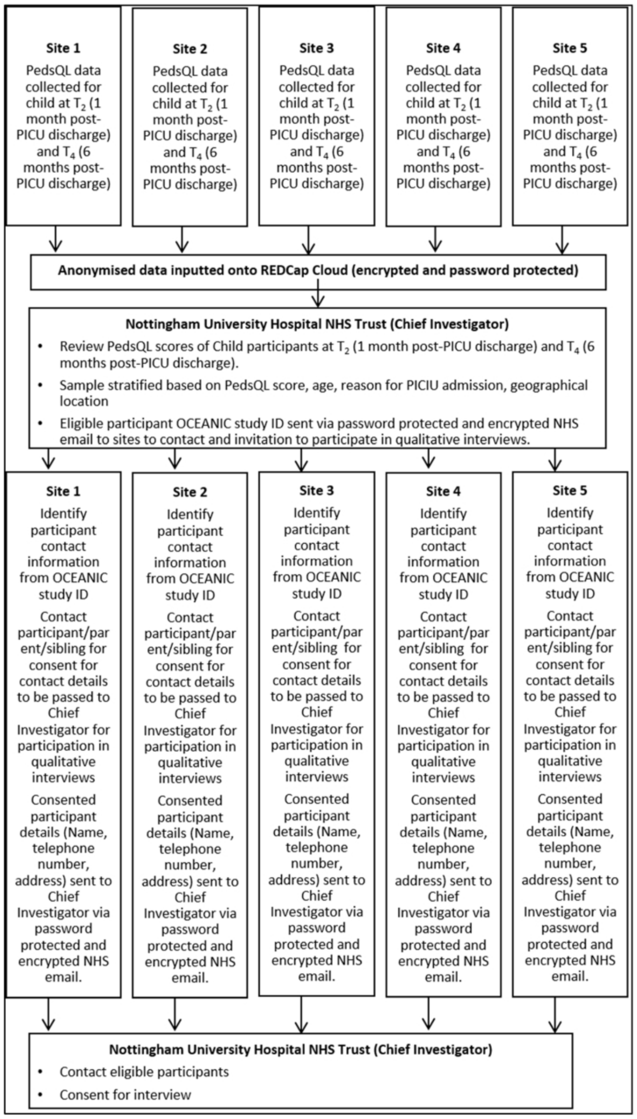

Identification and recruitment of participants for Work Package 2- Qualitative Study

173x246mm (300 x 300 DPI)

The OCEANIC study – Summary of measures used

Child related measures

PedsQL™ 4.0 (Pediatric Quality of Life Inventory) Generic Core Scales *(2-17 years)* and Infant Scales *(1-23 months)* – Acute Version measures HRQOL in children and adolescents aged 1 month to 17 years old. Both sets of instruments have good validity and reliability, have been widely used, [1-8] and can be completed in 5-7 minutes.[8-11] The instruments can discriminate between healthy children and those with a wide range of acute and chronic health conditions.

PedsQL™ Multi-dimensional Fatigue Scale *(2-17 years)* – Acute Version [12] is an 18-item scale that encompasses three domains: (1) General Fatigue, (2) Sleep/Rest Fatigue and (3) Cognitive Fatigue. The Multidimensional Fatigue Scale comprises parallel child self-report and parent proxy-report formats. Items for each of the forms are essentially identical, differing in developmentally appropriate language, or first or third person tense.

PedsQL™ Pediatric Pain Questionnaire *(5-17 years)* is a generic symptom-specific instrument to measure pain in patients with acute and chronic health conditions. We will use question #1 and #2, which asks participants capable of self-reporting to identify a point on a 100 mm line that best shows the worst pain the child experienced 'now' and 'in the past week'. Anchors include "no hurting, no discomfort, or no pain" and "hurting a whole lot, very uncomfortable, severe pain". A parent report version will be used for child participants that are unable to self-report.

Functional Status Scale (FSS) *(1 month-17 years)* is a valid and reliable assessment method to quantify functional status.[13 14] The FSS includes 6 domains: mental status, sensory functioning, communication, motor function, feeding, and respiratory. The FSS is amenable to studies of this nature due to ease of administration, granularity, and objectivity of assessment compared to other available methods and has been used in other outcome studies.[13 15]

Pediatric Cerebral Performance Category (PCPC) and the **Pediatric Overall Performance Category (POPC) *(1 month – 17 years)*** quantify short-term cognitive impairments and functional morbidity.[16 17] The POPC scale is dependent on the PCPC scale, as the PCPC status is included in POPC. Scores range from 1 to 6 for both scales with 1: good, 2: mild disability, and 6: brain death. Studies of patients with scores of 1–4 at PICU discharge, hospital discharge,

For peer review only

and one- and six-month follow-up show association with the Stanford Binet Intelligence Quotient, Bayley scales, and Vineland Adaptive Behaviour Scale. [16-19]

Strengths and Difficulties Questionnaire (SDQ) ***(2-17 years)*** [20 21] is a behavioural screening questionnaire used to evaluate emotional symptoms, conduct problems, hyperactivity/inattention, peer relationship problems, and prosocial behaviour. The SDQ quantifies low, medium, and high risk of emotional, behavioural, hyperactivity concentration disorders, or any disorder.

Child Revised Impact of Events Scale (CRIES-8) ***(7-17 years)*** is an eight-item screen for post-traumatic stress symptoms in children aged between 7 and 18 years, with established reliability and validity.[22] It has been used previously in the PICU population.[23] A cut-off score of 17 or greater has been found to classify correctly over 80% of children with a diagnosis of post-traumatic stress disorder.[24]

Children's Hope Scale (CHS) ***(8-17 years)*** [25] is a brief, six-item self-report measure of children's perceptions that their goals can be met. It has been validated for use in children and young people aged 8-17 years consisting of both healthy populations and children with a range of physical illnesses. Internal consistency estimates (alpha) for the samples ranged from 0.72 to 0.86. Test-retest reliability estimates (over a one-month interval) ranged from 0.71 to 0.73 [25].

Parent related measures

PedsQL™ Family Impact Module (FIM) Version 2.0 measures the impact of pediatric health conditions on family functioning [26]. It is completed by the parents and includes eight dimensions (physical functioning; emotional functioning; social functioning; cognitive functioning; communication; worry; daily activities; family relationships).

State-Trait Anxiety Inventory 6 (STAI-6) [27] is a self-reported questionnaire that assesses symptoms of anxiety. It is a short version of the Spielberger State Anxiety Scale (SSA), with a cut-off point at 1 SD above the mean to indicate clinically relevant symptoms[27].

Patient Health Questionnaire-4 (PHQ-4) [28] is a 4 item inventory rated on a 4 point Likert-type scale. Its purpose is to allow for very brief and accurate measurement of depression and anxiety. PHQ–4 scores are strongly associated with decrements in multiple domains of

For peer review only

functional impairment; the anxiety and depression subscales make unique overall contributions to the PHQ–4, both in terms of factorial and criterion validity; and perhaps most importantly: the results indicate that anxiety has a substantial independent effect on functioning, and even more so when comorbid with depression.

The Post Traumatic Stress Disorder (PTSD) Checklist for DSM-5 (PCL-5) is a 20-item self-report measure that assesses the presence and severity of PTSD symptoms [29]. Items on the PCL-5 correspond with DSM-5 criteria for PTSD. The PCL-5 can be used to quantify and monitor symptoms over time, to screen individuals for PTSD, and to assist in making a provisional or temporary diagnosis of PTSD. The PCL-5 is a psychometrically sound measure of DSM-5 PTSD. It is valid and reliable, useful in quantifying PTSD symptom severity, and sensitive to change over time [30 31].

Sibling related measures

The **PedsQL™ 4.0, SDQ** and **Children's Hope Scale** (as outlined in the Child related measures section above) will also be administered to sibling participants.

Multidimensional Assessment of Caring Activities (MACA-YC18) ***(8-17 years)*** [32] is an 18-item self-report measure that can be used to provide an index of the total amount of caring activity undertaken by the young person, as well as six subscale scores for domestic tasks, household management, personal care, emotional care, sibling care, and financial/practical care. The MACA-YC18 was designed as a short, easy to use, psychometric measure able to provide an index of the extent of caring activities that the young person is currently engaged in. Higher scores indicate greater levels of caring activity.[32 33]

Positive and Negative Outcomes of Caring (PANOC-YC20) ***(8-17 years)*** [32] a self-report measure that can be used to provide an index of positive and negative outcomes of caring. The PANOC-YC20 consists of two 10-item subscales: (1) positive responses, and (2) negative responses, which collectively assess the subjective cognitive and emotional impact of caring in young people with higher scores indicating greater positive and negative responses, respectively.

For peer review only

For peer review only

1. Pollack MM, Holubkov R, Funai T, et al. Pediatric intensive care outcomes: development of new morbidities during pediatric critical care. *Pediatr Crit Care Med* 2014;15(9):821-7. doi: 10.1097/PCC.0000000000000250 [published Online First: 2014/09/17]
2. Seid M, Limbers CA, Driscoll KA, et al. Reliability, validity, and responsiveness of the pediatric quality of life inventory (PedsQL) generic core scales and asthma symptoms scale in vulnerable children with asthma. *J Asthma* 2010;47(2):170-7. doi: 10.3109/02770900903533966 [published Online First: 2010/02/23]
3. Seid M, Varni JW, Gidwani P, et al. Problem-solving skills training for vulnerable families of children with persistent asthma: report of a randomized trial on health-related quality of life outcomes. *J Pediatr Psychol* 2010;35(10):1133-43. doi: 10.1093/jpepsy/jsp133 [published Online First: 2010/01/12]
4. Varni JW, Seid M, Knight TS, et al. The PedsQL 4.0 Generic Core Scales: sensitivity, responsiveness, and impact on clinical decision-making. *J Behav Med* 2002;25(2):175-93. doi: 10.1023/a:1014836921812 [published Online First: 2002/04/30]
5. Varni JW, Seid M, Smith Knight T, et al. The PedsQL in pediatric rheumatology: reliability, validity, and responsiveness of the Pediatric Quality of Life Inventory Generic Core Scales and Rheumatology Module. *Arthritis Rheum* 2002;46(3):714-25. doi: 10.1002/art.10095 [published Online First: 2002/03/29]
6. Mistry RD, Stevens MW, Gorelick MH. Health-related quality of life for pediatric emergency department febrile illnesses: an evaluation of the Pediatric Quality of Life Inventory 4.0 Generic Core Scales. *Health Qual Life Outcomes* 2009;7:5. doi: 10.1186/1477-7525-7-5 [published Online First: 2009/01/31]
7. Brandow AM, Brousseau DC, Pajewski NM, et al. Vaso-occlusive painful events in sickle cell disease: impact on child well-being. *Pediatr Blood Cancer* 2010;54(1):92-7. doi: 10.1002/pbc.22222 [published Online First: 2009/08/05]
8. Aspesberro F, Fesinmeyer MD, Zhou C, et al. Construct Validity and Responsiveness of the Pediatric Quality of Life Inventory 4.0 Generic Core Scales and Infant Scales in the PICU. *Pediatr Crit Care Med* 2016;17(6):e272-9. doi: 10.1097/PCC.0000000000000727 [published Online First: 2016/06/05]
9. Ong C, Lee JH, Leow MK, et al. Functional Outcomes and Physical Impairments in Pediatric Critical Care Survivors: A Scoping Review. *Pediatr Crit Care Med* 2016;17(5):e247-59. doi: 10.1097/PCC.0000000000000706 [published Online First: 2016/04/01]
10. Janssens L, Gorter JW, Ketelaar M, et al. Health-related quality-of-life measures for long-term follow-up in children after major trauma. *Quality of Life Research* 2008;17(5):701-13. doi: 10.1007/s11136-008-9339-0 [published Online First: 2008/04/26]
11. Eiser C, Morse R. The measurement of quality of life in children: past and future perspectives. *Journal of Developmental & Behavioral Pediatrics* 2001;22(4):248-56. [published Online First: 2001/09/04]
12. Varni JW, Burwinkle TM, Katz ER, et al. The PedsQL in pediatric cancer: reliability and validity of the Pediatric Quality of Life Inventory Generic Core Scales, Multidimensional Fatigue Scale, and Cancer Module. *Cancer* 2002;94(7):2090-106. doi: 10.1002/cncr.10428 [published Online First: 2002/04/05]
13. Aspesberro F, Mangione-Smith R, Zimmerman JJ. Health-related quality of life following pediatric critical illness. *Intensive Care Med* 2015;41(7):1235-46. doi: 10.1007/s00134-015-3780-7 [published Online First: 2015/04/09]
14. Pollack MM, Holubkov R, Glass P, et al. Functional Status Scale: new pediatric outcome measure. *Pediatrics* 2009;124(1):e18-28. doi: 10.1542/peds.2008-1987 [published Online First: 2009/07/01]

For peer review only

15. Pollack MM, Holubkov R, Funai T, et al. Simultaneous Prediction of New Morbidity, Mortality, and Survival Without New Morbidity From Pediatric Intensive Care: A New Paradigm for Outcomes Assessment. *Crit Care Med* 2015;43(8):1699-709. doi: 10.1097/CCM.0000000000001081 [published Online First: 2015/05/20]
16. Fiser DH, Long N, Roberson PK, et al. Relationship of pediatric overall performance category and pediatric cerebral performance category scores at pediatric intensive care unit discharge with outcome measures collected at hospital discharge and 1-and 6-month follow-up assessments. *Critical care medicine* 2000;28(7):2616-20.
17. Fiser DH, Tilford JM, Roberson PK. Relationship of illness severity and length of stay to functional outcomes in the pediatric intensive care unit: a multi-institutional study. *Critical care medicine* 2000;28(4):1173-79. [published Online First: 2000/05/16]
18. Fiser DH. Assessing the outcome of pediatric intensive care. *The Journal of pediatrics* 1992;121(1):68-74. [published Online First: 1992/07/01]
19. Fuchs S, Lewis RJ. Tools for the measurement of outcome after minor head injury in children: summary from the Ambulatory Pediatric Association/EMSC Outcomes Research Conference. *Academic emergency medicine* 2003;10(4):368-75. [published Online First: 2003/04/03]
20. Goodman R. Psychometric properties of the strengths and difficulties questionnaire. *Journal of the American Academy of Child & Adolescent Psychiatry* 2001;40(11):1337-45. doi: 10.1097/00004583-200111000-00015 [published Online First: 2001/11/09]
21. Goodman R. The Strengths and Difficulties Questionnaire: A Research Note. *Journal of Child Psychology and Psychiatry* 1997;38(5):581-86. doi: 10.1111/j.1469-7610.1997.tb01545.x [published Online First: 1997/07/01]
22. Yule W. Anxiety, depression and post-traumatic stress in childhood. . In: Sclare I, ed. Child psychology portfolio. Windsor, UK: NFER-Nelson 1997:35-38.
23. Colville G, Pierce C. Patterns of post-traumatic stress symptoms in families after paediatric intensive care. *Intensive Care Med* 2012;38(9):1523-31. doi: 10.1007/s00134-012-2612-2 [published Online First: 2012/06/19]
24. Perrin S, Meiser-Stedman R, Smith P. The Children's Revised Impact of Event Scale (CRIES): Validity as a screening instrument for PTSD. *Behavioural and Cognitive Psychotherapy* 2005;33(4):487-98.
25. Snyder CR, Hoza B, Pelham WE, et al. The Development and Validation of the Children's Hope Scale. *Journal of Pediatric Psychology* 1997;22(3):399-421. doi: 10.1093/jpepsy/22.3.399
26. Varni JW, Sherman SA, Burwinkle TM, et al. The PedsQL Family Impact Module: preliminary reliability and validity. *Health Qual Life Outcomes* 2004;2:55. doi: 10.1186/1477-7525-2-55 [published Online First: 2004/09/29]
27. Marteau TM, Bekker H. The development of a six-item short-form of the state scale of the Spielberger State—Trait Anxiety Inventory (STAI). *British Journal of Clinical Psychology* 1992;31(3):301-06. doi: 10.1111/j.2044-8260.1992.tb00997.x
28. Kroenke K, Spitzer RL, Williams JB, et al. An ultra-brief screening scale for anxiety and depression: the PHQ–4. *Psychosomatics* 2009;50(6):613-21.
29. Weathers FW, Litz BT, Keane TM, et al. The PTSD checklist for DSM-5 (PCL-5). US: Department of Veteran Affairs: National Center for PTSD 2013.
30. Blevins CA, Weathers FW, Davis MT, et al. The posttraumatic stress disorder checklist for DSM-5 (PCL-5): Development and initial psychometric evaluation. *Journal of traumatic stress* 2015;28(6):489-98. doi: 10.1002/jts.22059 [published Online First: 2015/11/26]

For peer review only

31. Bovin MJ, Marx BP, Weathers FW, et al. Psychometric properties of the PTSD Checklist for Diagnostic and Statistical Manual of Mental Disorders–Fifth Edition (PCL-5) in veterans. *Psychological Assessment* 2016;28(11):1379.
32. Joseph S, Becker F, Becker S. Manual for measures of caring activities and outcomes for children and young people. Nottingham: The University of Nottingham 2009.
33. Joseph S, Becker S, Becker F, et al. Assessment of caring and its effects in young people: development of the Multidimensional Assessment of Caring Activities Checklist (MACA-YC18) and the Positive and Negative Outcomes of Caring Questionnaire (PANOC-YC20) for young carers. *Child: care, health and development* 2009;35(4):510-20. doi: 10.1111/j.1365-2214.2009.00959.x [published Online First: 2009/03/27]

For peer review only
