# Supplementary material for: Study protocol for a multicentre longitudinal mixed methods study to explore the Outcomes of ChildrEn and fAmilies in the first year after paediatric Intensive Care: the OCEANIC study
Source: BMJ Open. 2020 May 17;10(5):e038974. doi: 10.1136/bmjopen-2020-038974 (PMC7239532; doi:10.1136/bmjopen-2020-038974)
Supplement: Reviewer comments [file bmjopen-2020-038974.reviewer_comments.pdf]

PEER REVIEW HISTORY

BMJ Open publishes all reviews undertaken for accepted manuscripts. Reviewers are asked to complete a checklist review form (**http://bmjopen.bmj.com/site/about/resources/checklist.pdf**) and are provided with free text boxes to elaborate on their assessment. These free text comments are reproduced below.

ARTICLE DETAILS

**TITLE (PROVISIONAL)**	A study protocol for a multi-centre longitudinal mixed methods study to explore the Outcomes of ChildrEn and fAmilies in the first year after paediatric Intensive Care: The OCEANIC Study
**AUTHORS**	Manning, Joseph; Latour, Jos; Curley, Martha; Draper, Elizabeth; Jilani, Tahseen; Quinlan, Philip; Watson, R; Rennick, Janet; Colville, Gillian; Pinto, Neethi; Latif, Asam; Popejoy, Emma; Coad, Jane

This article was not externally reviewed at BMJ Open. Protocols that have been independently assessed prior to submission to BMJ Open are usually fast-tracked to publication on the grounds that further substantial changes will not be possible. This independent assessment will usually be external, independent review for both a substantial grant award from a non-commercial or government funder and ethics approval.
